# Stable, Efficient, and Scalable Two‐Dimensional Perovskites for Next‐Generation Solar Cells

**DOI:** 10.1002/smsc.70393

**Published:** 2026-09-07

**Authors:** Kaushik Bhat, Sangita Das, Anurag Roy, Partha Pratim Das

**Affiliations:** ^1^ Manipal Institute of Technology Manipal Academy of Higher Education Manipal India; ^2^ Department of Biochemistry School of Chemical Sciences St Joseph's University Bengaluru Karnataka India; ^3^ Environment and Sustainability Institute University of Exeter Penryn Cornwall UK

**Keywords:** 2D perovskite, charge transport, crystallization control, environmental stability, large‐scale solar cells, photovoltaic performance

## Abstract

Metal halide perovskites have rapidly evolved from a niche class of semiconductors to a leading platform for advanced photovoltaic technologies. Within this family of materials, two‐dimensional (2D) perovskites have emerged as structurally engineered derivatives that offer enhanced environmental stability relative to their three‐dimensional counterparts. However, reduced dimensionality also introduces a distinct optoelectronic regime governed by confinement effects and structural anisotropy, which complicates the direct translation of established design strategies. This review presents a unified perspective on the design and optimization strategies of 2D perovskite solar cells, including how spacer chemistry, inorganic slab thickness, and crystallization kinetics jointly influence transport anisotropy, film formation, and optical properties with implications for stability and device performance. Finally, the scalable integration pathways, including blade coating, printing techniques, machine learning‐guided fabrication, and vapor deposition techniques for 2D perovskite materials for solar cell fabrication, are considered.

## Graphical abstract

Two‐dimensional perovskites are presented as a versatile platform for photovoltaic research, combining enhanced stability with competitive efficiencies. This review analyses 2D perovskite structure–property relationships linking crystallographic orientation and phase distribution and implications in stability and performance. Emerging opportunities in scalable processing are presented to outline viable pathways toward commercially relevant 2D perovskite photovoltaics.

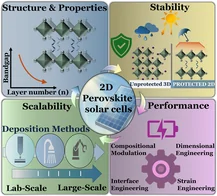

## 1 Introduction

Metal halide perovskite solar cells (PSCs) have rapidly advanced to certified power conversion efficiencies (PCE) above 26% in less than two decades of development, which is now competitive with that of silicon photovoltaic cells, owing to their desirable properties and quick progress in material design and device engineering [1, 2, 3]. The three‐dimensional (3D) ABX_3_ structure, where A is a monovalent cation such as methylammonium (MA^+^), formamidinium (FA^+^) or cesium (Cs^+^), B is a divalent metal cation such as lead (Pb_2_^+^) or tin (Sn_2_^+^), and X is a halide anion (Cl^−^, Br^−^, I^−^), combines long carrier diffusion lengths, strong light absorption and benign defect properties [4, 5]. Despite their remarkable efficiency, their intrinsic instability under humidity, thermal stress, and ultraviolet illumination remains a major barrier to commercialization [6, 7, 8, 9, 10, 11].

**FIGURE 1:**
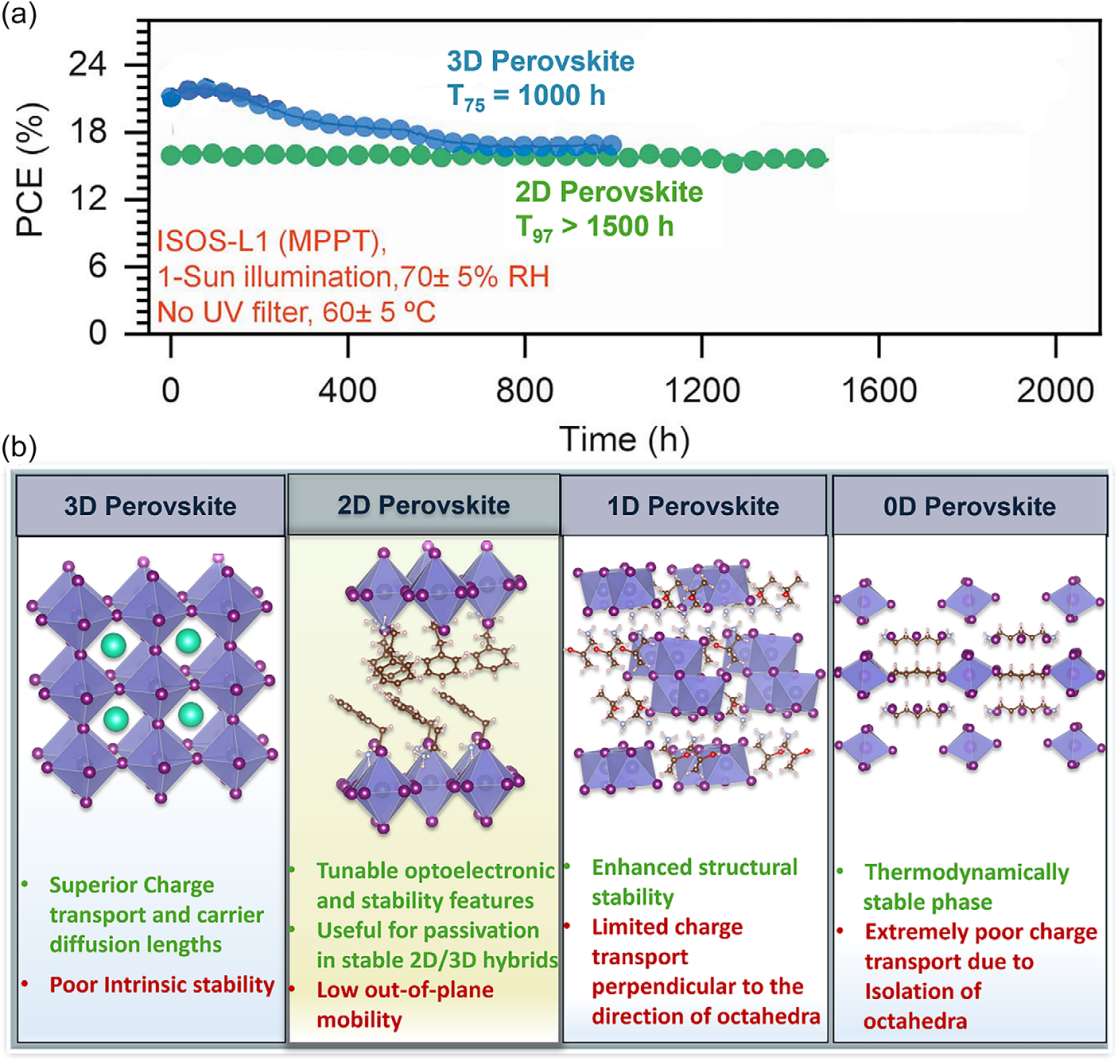
(a) ISOS‐L‐1 measurements at maximum power point (MPP) tracking for encapsulated perovskite solar cells, comparing layered (2D) and bulk 3D perovskite compositions. Reproduced with permission [12]. Copyright 2022, The American Association for the Advancement of Science. (b) Comparison of dimensionality‐dependant properties in perovskite materials.

In this context, the adoption of low‐dimensional perovskites, such as two‐dimensional (2D) perovskites, has been considered for advanced operational stability (see Figure 1a) [12, 13, 14]. 2D metal‐halide perovskites are formed by introducing organic spacers into 3D frameworks and breaking the Goldschmidt tolerance factor (which provide a guideline for ideal cubic phase and its degree of distortion from the cubic phase) [15]. The higher stability coupled with tunable energy bands due to their layered design, suppressed ion migration, and defect passivation for 3D perovskites enables its great potential for use in photovoltaic devices (Figure 1b) [16]. The superior stability primarily stems from the hydrophobic properties of the spacer layer and its highly oriented structure with dense packing, which serves as an effective shield against environmental stressors like moisture and oxygen, thereby limiting internal ion migration [17, 18, 19].

The very dimensional reduction that improves stability also reshapes the fundamental photophysics and transport characteristics that can impede device performance [20, 21, 22]. In particular, the intercalation of an organic spacer into the perovskite lattice induces quantum and dielectric confinement effects, which increase exciton binding energy and create potential barriers to carrier migration. Moreover, 2D films formed from mixed spacer or 3D precursor solutions naturally form complex phases that can either facilitate beneficial energy funneling or if uncontrolled, may generate higher recombination active interfaces that block conductivity [23, 24]. Despite this, recent advancements in interface engineering, hydrazide‐enabled orbital coupling within structures, and solvent‐environment control have led to the development of 2D PSCs with efficiencies exceeding beyond 22% [25, 26, 27]. The phase distribution and controlled vertical alignment of the layered perovskite domains are the primary microstructural domains that facilitate enhanced out‐of‐plane charge extraction, thereby achieving high efficiencies [28, 29, 30, 31, 32]. And those of phenethylammonium (PEA)‐based 2D PSCs could be prepared in ambient conditions and exhibited better stability than methylammonium lead iodide (MAPbI_3_)‐based PSCs [33].

The question of scalability adds an additional constraint, often overlooked, on what constitutes a successful 2D perovskite‐based device design. Many of the impressive 2D‐based PSCs demonstrations rely on laboratory‐scale techniques such as spin coating and glovebox processing that do not translate directly to large‐area production processes [34, 35]. Nevertheless, there is growing evidence that layered perovskites are compatible with scalable fabrication routes [36, 37, 38, 39, 40]. Further, from a device‐engineering standpoint, layered 2D perovskite materials have been deployed across multiple architectures, including planar, inverted, and mesoporous structures, where they function not as a single material but as an instrument or as a resource for dimensional engineering [41, 42, 43, 44, 45, 46, 47, 48]. As such, they can be mostly utilized as an interfacial modifier on 3D absorbers as ultrathin surface layers, which reduce nonradiative recombination and often yield substantial gains in open‐circuit voltage (*V*_oc_).

Against this background, the current review has four interconnected objectives. First, we dissect fundamental descriptors of 2D phases that primarily responsible for stability and subsequent performance. Secondly, we assess long‐term stability and operational lifetimes in terms of material aspects. Third, we evaluate understanding of the performance metrics of PSCs and their improvement strategies. Finally, we critically evaluate the present and prospects of scalable fabrication methods for 2D perovskite‐based devices and reliability to determine which approaches are most likely to narrow the gap between lab‐scale prototypes and commercially viable solar cell devices.

## 2 Fundamental Descriptors of 2D Perovskites

### 2.1 Chemical Structure and Dimensionality

**FIGURE 2:**
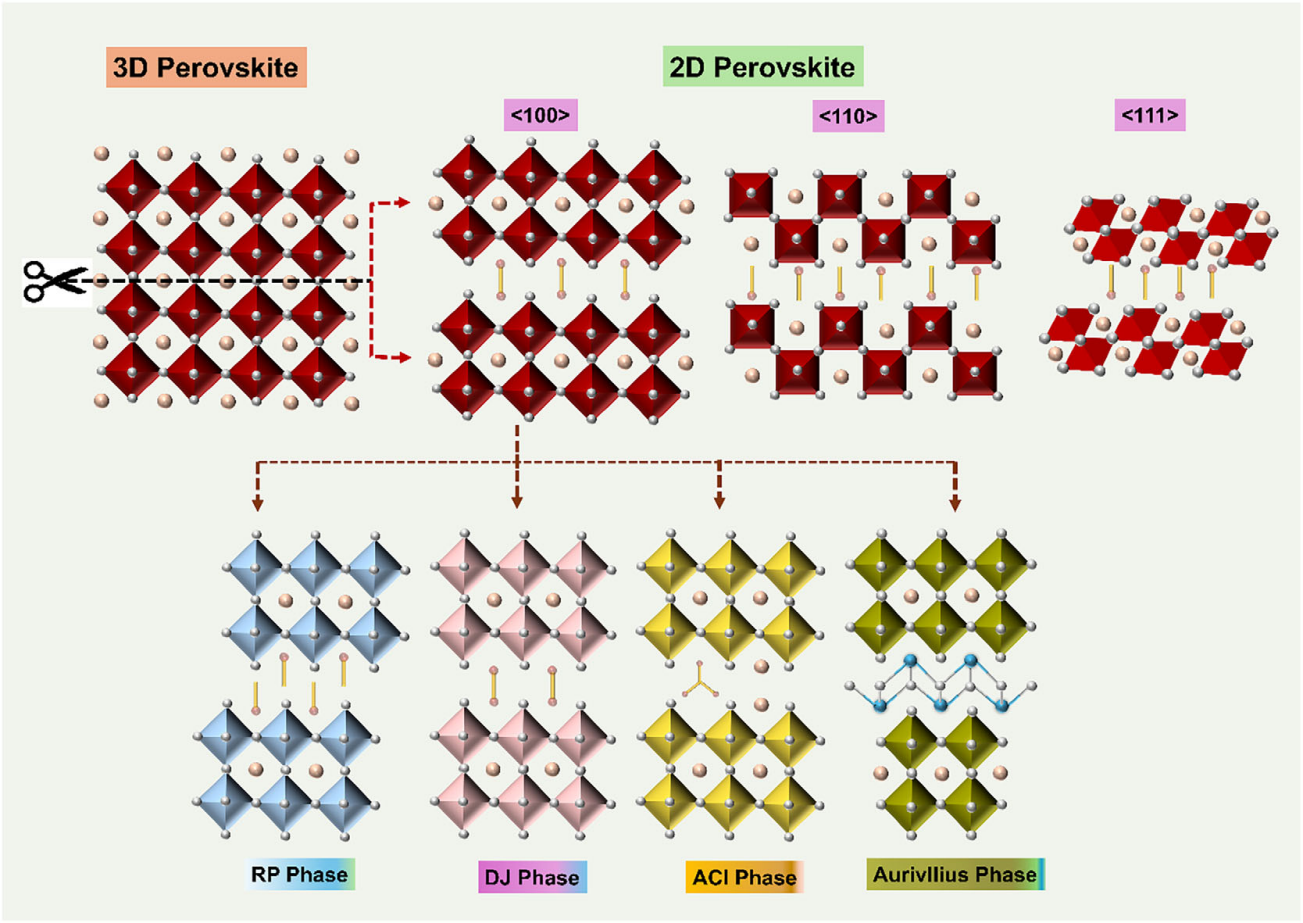
Comparison of 3D, quasi‐2D perovskite structures (*n* = 2) derived from slicing a cubic perovskite lattice along different crystallographic planes ((100), (110), and (111)) and representative structural families of 2D perovskites from (100) plane.

Layered 2D halide perovskites can be rationalized as reduced‐dimensional derivatives of 3D ABX_3_ framework, in which inorganic slabs of corner‐sharing BX_6_ octahedra are periodically separated by bulky organic spacer cations [20, 49]. They are described by a general formula (A^1^)_*m*_(A)_*n*−1_B_*n*_X_3*n*+1_, where A can be divalent (*m* = 1) or monovalent (*m* = 2) cations forming a bilayer or monolayer connecting the inorganic A_*n*−1_B_*n*_X_3*n*+1_ 2D sheets, where *n* represents the layer thickness of metal halide sheets that can be adjusted by tuning precursor composition, that is, indicates the number of octahedral layers [50]. When *n* = 1 phase, the perovskite is termed as pure 2D, and quasi‐2D perovskite when *n* > 1. Because 2D layered perovskites are obtained by slicing a 3D perovskite along specific crystallographic planes (Figure 2), their structures are generally grouped into <100>, <110>, and <111> oriented families [20, 51]. Among these, <100>‐oriented 2D perovskites have become the dominant structural motif in photovoltaics. <100> oriented 2D layers enable the incorporation of various spacer cations between 2D perovskites. On the other hand, <110> and <111>‐oriented layers offer intriguing dielectric and excitonic properties but typically present greater difficulties in forming low‐defect thin films suitable for scalable solar cells.

Within the <100> family, the chemical identity and binding mode of the interlayer spacer A^1^ define the principal subclasses, namely Ruddlesden–Popper (RP) phases, the Dion–Jacobson phases, the alternating cations in the interlayer (ACI) phase, and Aurivillius phases. The Ruddlesden–Popper (RP) family have the general formula R_2_A_*n*−1_B_*n*_X_3*n*+1_, where BX_6_ octahedra assemble into perovskite sheets of thickness *n*, A is a small monovalent cations, R is a long‐chain or aromatic spacer, and X is a halide anion [18, 52]. The interlayer cations (R) for the interlayer of the RP phase together with the inorganic structure, forming a quantum well structure (QW) structure. Between the interlayers, there are weak van der Waals forces between the two layers of interlayer. On the outer side of the interlayer, halide ions are connected to the interlayer cations through hydrogen bonding and electrostatic interactions, which are facilitated by the terminal ammonium groups. When observed along stacking axis, the inorganic layers show an offset of (1/2, 1/2) within the plane [53].

Beyond RP type phases, Dion–Jacobson (DJ) phases replace pair of monofunctional spacers with a divalent organic cation, yielding a general formula DA_*n*−1_B_*n*_X_3*n*+1_, where adjacent inorganic slabs are directly bridged rather than separated by van der Waals gaps [32]. This bridging shortens the interlayer distance, enhances interlayer electron coupling, and often improves environmental and thermal robustness. Here the inorganic layers adopt an eclipsed stacking configuration with zero in‐plane displacement, meaning one layer is perfectly aligned over the another, exhibiting an offset of (0, 0) within the inorganic layer plane [54]. DJ phases are often considered structurally advantageous compared to RP phases due to bridging of adjacent slabs by diammonium spacers, which fortifies crystal framework. However, this structural advantage does not inherently indicate DJ systems exhibit superior device performance or stability under all conditions. RP phases can still outperform DJ in specific regimes, such as thick‐film stability tests and certain heterojunction architectures, where thermochemical robustness, interfacial recombination control, or strain tolerance become more decisive. For instance, calorimetric analysis reveals that RP perovskites demonstrate negative enthalpies of formation, conferring superior thermochemical stability compared to DJ materials, which exhibit positive formation enthalpies and consequently exhibit air durability lower than their RP counterparts (6 days versus 120 days) [55]. Additionally, first‐principles calculations for 2D/3D heterojunction devices reveal that RP‐based perovskite heterojunctions have significantly reduced lower interfacial charge recombination compared to DJ‐based architectures, indicating that structural rigidity in DJ‐systems amplifies interfacial charge exchange and recombination losses rather than minimizing them [56]. Thus, although, DJ phases often provide stronger interlayer coupling and superior intrinsic stability, this advantage is conditional rather than universal.

**FIGURE 3:**
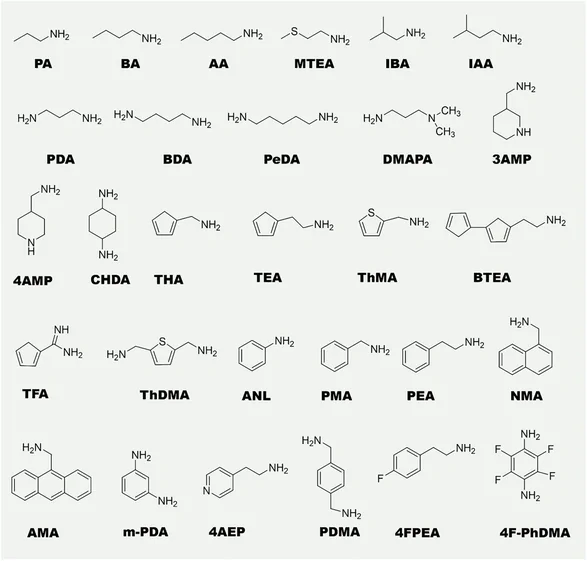
Commonly used organic spacers in 2D perovskites.

Aurivillius are another type of 2D perovskite having general formula A_2_^1^X_2_A_*n*−1_B_*n*_X_3*n*+1_. In this structure, excessive X‐site anions are incorporated into the intercalation of adjacent perovskite‐like slabs of the RP phase perovskites [57]. These perovskites are comparatively less explored for photovoltaic applications. ACI perovskites feature alternating interlayer cations within the spacer region and follow the general formula A^1^A_*n*_B_*n*_X_3*n*+1_. Structurally, ACI systems exhibit asymmetric displacement, and the layers combine stacking of RP and DJ phases, showing (0, 1/2) displacement [28]. The alternating cation environment can introduce internal dipolar fields and modulate interlayer electronic interactions. Recent studies have proposed that light‐induced out‐of‐plane lattice contraction in DJ and ACI phases may enhance Iodine–Iodine (I–I) interactions across the organic barrier, thereby improving carrier mobility and photovoltaic response [58]. However, as both mono and diammonium cations are present within the perovskite structure, which either improves or worsens the stability compared to a pure DJ perovskite, depending on the cation used, which was ascribed to film morphology, defect passivation, and crystallinity improvement. These subtle structural distinctions, including bilayer and single layer organic spacers as presented in Figure 3, stacking, and magnitude of layer shift, directly influence interlayer distance, octahedral tilting, and connectivity of electronic wavefunctions across the organic barriers, thereby governing carrier transport and overall photovoltaic performance and stability.

### 2.2 Electronic Structure and Excitonic Confinements

The fundamental distinction mechanism between 3D and 2D perovskite systems is found in the characteristics of photogenerated carriers and their transport behavior. In 3D bulk perovskites, the extended polar lattice provides strong dielectric screening, leading to the formation of weakly bound Wannier excitons with binding energies ranging from 10 to 35 meV [59, 60]. This low binding energy facilitates significant dissociation of excitons into free charge carriers at room temperature, leading to a transport regime dominated by carrier‐mediated transport. In this region, electrons and holes diffuse largely independently through the material while remaining spatially correlated due to residual coulombic attraction. Despite having modest diffusion coefficients of 0.01–0.05 cm^2 ^s^−1^, 3D perovskites show remarkably long diffusion lengths, extending from hundreds of nanometers to micrometers. This exceptional behavior is attributed to defect‐tolerant electronic structure that minimizes trap‐assisted recombination and the spatial separation of electron and hole polarons that delays their recombination [61, 62]. In sharp contrast, 2D perovskites, which are characterized by strong quantum confinement effects, produce excitons with significantly higher binding energies, ranging from approximately 50 meV in thicker layers (higher *n*) to approximately 500 meV in the thinnest structures (*n* = 1) [63, 64]. This strong confinement results in excitons becoming localized within single perovskite layers, creating an exciton‐dominated regime even though free carriers coexist due to efficient dissociation mechanisms.

**FIGURE 4:**
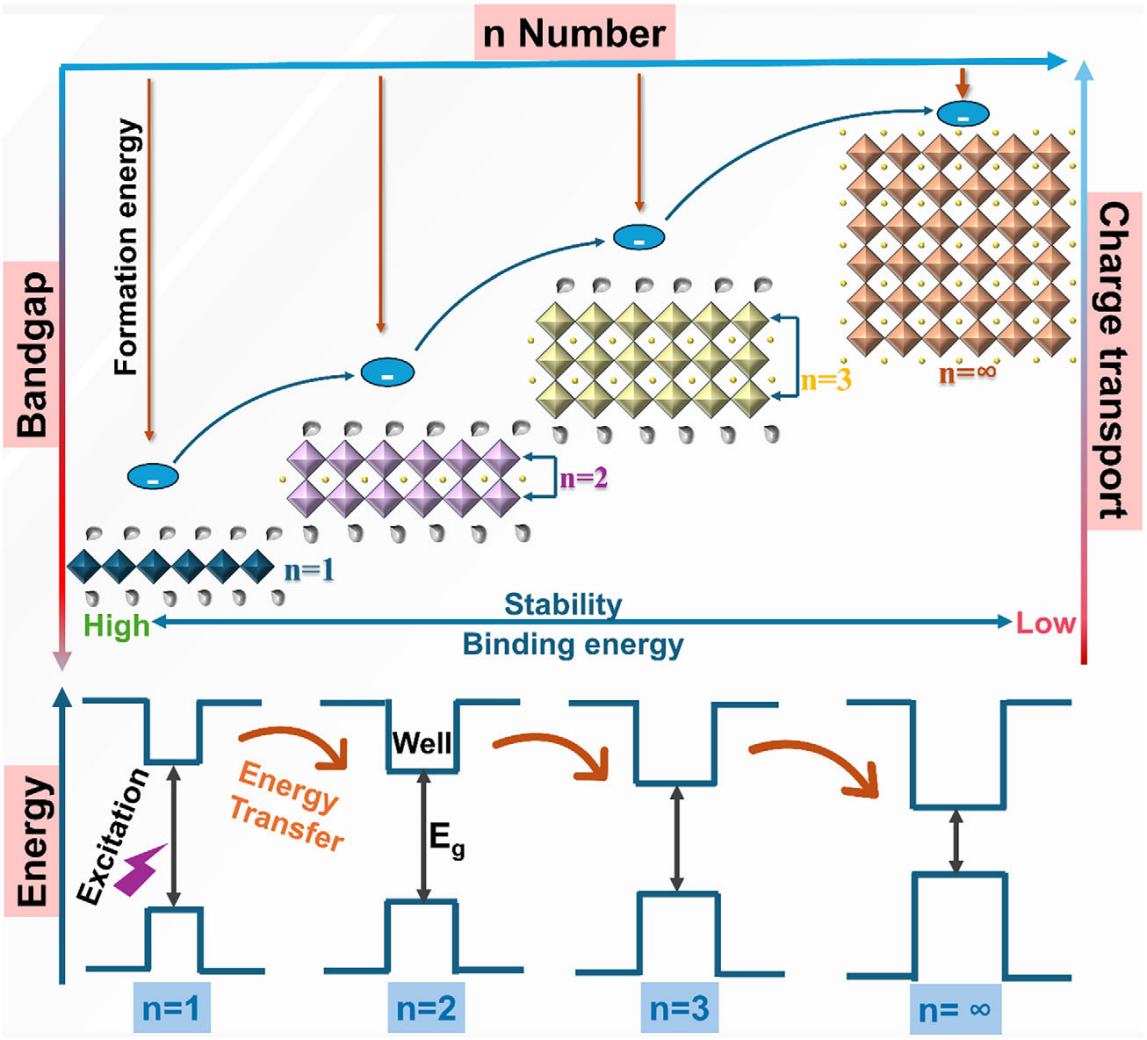
Schematic illustration of the variation in bandgap, formation energy, and exciton binding energy with dimensional evolution for different values of *n* with transfer of charge from small‐*n* to large‐*n* phases.

An important characteristic property of 2D perovskite is the strong anisotropy in charge‐carrier transport due to their structural asymmetry, while the anisotropy in exciton transport is comparatively less pronounced. In the in‐plane directions within the inorganic perovskite layers, excitons typically propagate through band‐like diffusion of their center of mass, interrupted by scattering with phonons and defects, similar to 3D perovskites described by a diffusion equation

(1) $$\frac{\partial n}{\partial t}=D\nabla^{2}n-\frac{n}{\tau }$$

where *D* is the in‐plane exciton diffusivity, *n* is the exciton density distribution, and *τ* is the exciton lifetime [65]. Typically, the exciton diffusion length is defined as $LD=\sqrt{D\tau }$ for one‐dimensional transport, although occasionally this expression is written with an additional factor $\sqrt{2}$ on the right‐hand side. The value of *D* is highly sensitive to lattice rigidity and vibrational spectra, which are influenced by the organic spacer composition. For instance, 2D perovskites with aromatic ligands such as phenethylammonium (PEA) demonstrate more rigid lattices due to π–π interactions and increased structural symmetry, resulting in in‐plane exciton diffusivities around 0.2 cm^2 ^s^−1^ and diffusion lengths of approximately 200 nm. Whereas softer alkylammonium spacers such as butylammonium (BA) introduce stronger lattice fluctuations and enhanced exciton‐phonon scattering, reducing and in‐plane diffusivities and diffusion lengths by roughly an order of magnitude under similar measurement conditions [65]. This suggests that exciton transport in 2D perovskites is not inherently poor, and it can be tuned through spacer chemistry and crystal packing geometry (see Section 4.1.1 for details). In addition, the transport of charge carriers in the out‐of‐plane direction is greatly hindered by the presence of electronically insulating organic spacers and disrupted octahedral connectivity. However, exciton transport can still occur via dipole‐mediated mechanism such as Förster resonance energy transfer, where energy is exchanged between adjacent quantum wells via long‐range dipole–dipole interactions, rather than through the physical movement of free electrons and holes [66]. Consequently, exciton movement in 2D perovskites exhibits considerably less anisotropy compared to charge carriers, whose mobilities are several orders of magnitude lower out‐of‐plane than in‐plane direction. Figure 4 provides the conceptual overview of the electronic structure and carrier dynamics in 2D perovskites as the layer number (*n*) increases. The transition from low‐*n* to high‐*n* compositions progressively reduces quantum confinement, thereby improving charge transport although inherent stability is compromised.

The efficient operation of solar cell devices, which collect light and convert it into electronic current, requires long exciton diffusion lengths [67]. This allows excitons to reach specific interfaces, where they subsequently dissociate into individual charge carriers that are then extracted from the device. Materials characterized by short diffusion lengths adversely affect device performance. They not only reduce the efficiency of exciton transport to device interfaces but also necessitate the use of absorbing layers that are insufficiently thick to capture all incoming light. When the diffusion length equals or surpasses the absorption depth, suitably thick absorbing layers may be used to catch the majority of the available light, with an additional increase in diffusion length yielding marginal improvements in the device performance. Light absorption by perovskite layers typically reaches saturation at perovskite thickness ranging from several hundred nanometers to a few micrometers [67, 68, 69]. These distances are equivalent to charge carrier diffusion lengths in 3D perovskites and quasi‐2D perovskites with high *n* values but often longer than diffusion lengths seen in 2D perovskites. This mismatch between required optical thickness and achievable exciton diffusion length is an intrinsic loss mechanism in low *n* quasi‐2D and pure 2D absorbers, preventing them from providing complete solar spectrum absorption. It explains why 3D perovskites, with their intrinsically long carrier diffusion lengths and free carrier‐dominated transport regime, may use relatively simple device structures and still achieve laboratory efficiencies beyond 26%, whereas 2D absorbers have not yet matched this performance.

**FIGURE 5:**
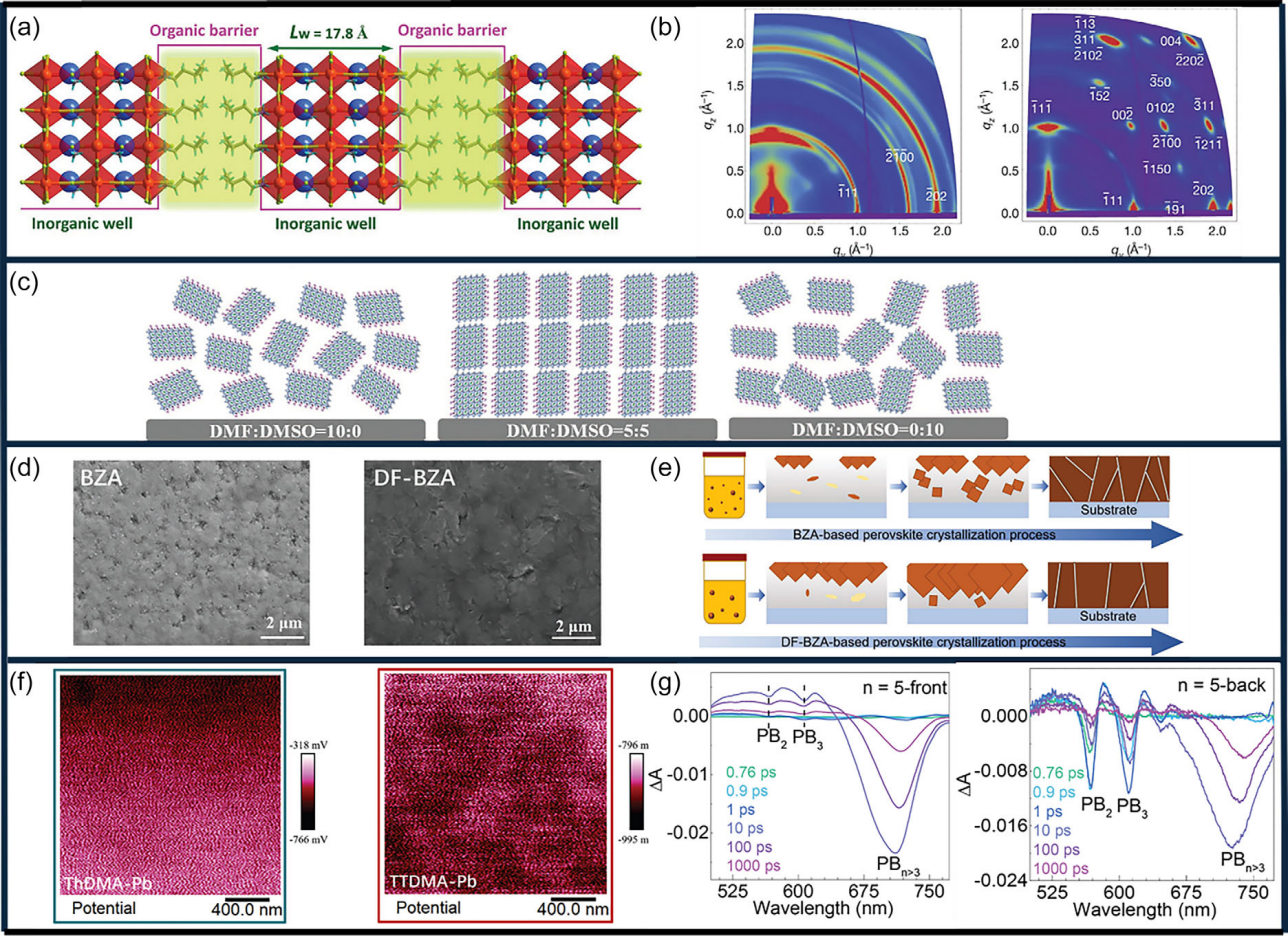
(a) Diagram of quantum‐well structure of 2D perovskites. Reproduced with permission [72]. Copyright 2020, Wiley‐VCH. (b) Representative GIWAXS patterns illustrating the evolution from polycrystalline to preferentially oriented (near‐single‐crystalline) films under different processing conditions. Reproduced with permission [73]. Copyright 2016, Springer Nature Limited. (c) Effect of solvent environment on 2D perovskite growth. Reproduced with permission [74]. Copyright 2019, Wiley‐VCH. (d) SEM images showing changes in grain size, surface morphology and roughness upon spacer modification and (e) schematic diagram of crystallization process and film formation of the corresponding perovskite. Reproduced with permission [75]. Copyright 2024, Wiley‐VCH. (f) Surface potential mapping of 2D perovskite films with different spacer chemistries. Reproduced with permission [76]. Copyright 2021, Wiley‐VCH. (g) Transient absorption spectroscopy at different delay times of 2D perovskite films from front excitation and back excitation with pronounced absorption photobleaching signals. Reproduced with permission [77]. Copyright 2017, American Chemical Society.

Beyond the intrinsic excitonic and transport anisotropies discussed above, the quantum well‐architecture and band alignment in 2D perovskites introduce an additional layer of complexity in how photogenerated carriers and excitations relax and migrate. The inorganic layer B_*n*_X_3*n*+1_ primarily dictates the fundamental electronic properties such as bandgap, carrier mobility, and optical absorption. While the organic layer doesn’t directly influence the band gap characteristics. Nevertheless, they influence B_*n*_X_3*n*+1_ structural features, including size, shape, charge distribution, and functional group positioning [70, 71]. The organic spacers are mostly electrically insulating and possess wide‐bandgap nature. Hence, the conduction band (CB) minimum of the perovskite slabs (inorganic layers) lies energetically below the lowest unoccupied molecular orbital organic spacer layers while the valence band (VB) maximum lies above their highest occupied molecular orbitals (HOMO) leading to type I band alignment. As a result, both electrons and holes are energetically confined or trapped within the inorganic layers (see Figure 5a) which thereby act as “well” and the wide‐bandgap (WBG) organic layer act as “barrier” [72].

The effective confinement is not purely electronic but is strongly amplified by dielectric contrast due to the disparity in dielectric constants between the organic spacer and inorganic components (relative permittivity *ε*_r_ organic ≈2–3 and *ε*_r_ inorganic ≈6–8) [78]. A lower dielectric constant of organic barriers (*ε*_b_) compared to that of inorganic wells (*ε*_w_) means the organic layer cannot effectively shield charges from excitons, which intensifies the Coulomb interaction, leading to increased binding energy [79]. Consequently, with the combined quantum and dielectric confinement effects, the efficient out‐of‐plane conduction is restricted unless layers are favorably oriented. Here, the quantum confinement length is defined by the thickness of the inorganic slab, which scales linearly with layer number (*n*‐value), while quantum barrier thickness (interlayer spacer) is governed by the length of the spacers and steric effect. In 2D perovskites, binding energy of an exciton is considerably high. These high binding energies can lead to stable excitons at normal temperature. The excitons in 2D perovskites are predominantly of the Wannier type, characterized by high confinement and smaller Bohr radii [63].

Beyond this conventional scenario, alternative configurations can be achieved by designing the organic spacer with specific molecular and compositional properties. In systems where the organic layer frontier orbitals overlap energetically with the band edges of inorganic slabs, a type‐II heterojunction may form that favor electrons and holes localization in different layers. Such alignment can facilitate interlayer charge transfer and introduce new low‐energy optical transitions resulting from inorganic–organic charge–transfer excitations [80]. Consequently, type‐II aligned 2D perovskites can display redshifted absorption onsets, narrow the band gaps, and possibly even an extension of absorption into the near‐infrared area. However, most of the 2D perovskites generally follow type I QW structures where energy transfer occurs between QWs, funneling carriers into the lowest bandgap domains. This process promotes carrier accumulation in these low‐bandgap regions, which ultimately impair charge movement and yield sub‐optimal efficiency outputs. To mitigate these limitations, engineering 2D perovskite films with a homogeneous energy landscape or phase‐pure structures is essential. For example, Yuan and coworkers employed (aminomethyl)piperidinium spacer cations to modulate crystallization kinetics, yielding an ordered QW distribution and uniform energy landscape. This strategy markedly suppresses recombination losses and quasi‐Fermi level splitting, enhancing carrier lifetimes and photovoltaic performance [81].

Besides these, density functional theory (DFT) analysis of crystal structure has revealed that lattice distortion and defect energetics also significantly influence the electronic structure of 2D perovskites. DFT calculations of defect formation energies show that iodine vacancies both in‐plane (*V*_I‐in_) and out‐of‐plane (*V*_I‐out_) have much lower formation energies in DJ crystals than in RP crystals [70]. In general, the formation energy is expressed as

(2) $$E_{f}=E_{\text{defect}}-E_{\text{pristine}}+\sum_{i}n_{i}\left(\mu_{i}+E_{i}\right)$$

where $E_{f}$ is the defect formation energy, $E_{\text{defect}}$ is the total energy of the defected structure, $E_{\text{pristine}}$ is the pristine lattice energy, and the last term accounts for the chemical potential of constituent species involved in the defect. Crystal structure analysis shows octahedral lattice distortion increases as the interlayer spacing decreases. This trend directly parallels the experimentally observed emission intensity, with more distorted DJ lattices prefer a higher density of iodine vacancy‐induced nonradiative recombination centers along the inorganic framework rather than within the organic spacer. Therefore, spacer and cation combinations that moderate octahedral distortion without compromising stability and targeted passivation of iodine‐vacancy sites can be helpful for minimizing the intrinsic defect‐mediated losses and maximizing the photovoltaic performance.

### 2.3 Crystallization Dynamics

For efficient charge separation and ultrafast charge transfer, vertically aligned crystal orientation is essential. Vertical orientation has been shown to promote directional carrier transport and reduce recombination effectively, thereby enhancing efficiency (Figure 5b) [73]. Therefore, a deep mechanistic understanding of crystallization dynamics and film formation is necessary for the fabrication of stable and efficient 2D PSCs. The crystallization of 2D perovskites is influenced by choice of solvent, annealing temperature, and the selectivity of the spacer cations.

Solvent choice modulates crystallization growth kinetics by altering precursor coordination and solvent–solute interactions. In one study, dimethylacetamide (DMAC), which has low polarity and a moderate boiling point, was shown to weakly coordinate with lead and ammonium salts and may easily escape during solution processing, thereby accelerating the crystallization rate of 2D Ruddlesden–Popper perovskite (RPP) [82]. Such perovskites found to have increased charge carrier lifetime with minimal trap density. A related study extensively investigated role of ink formulation, morphology, and coating temperature on the texture, quantum well orientation, and subsequent implications on the performance of the solar cell device [83]. Basically, ink formulation was altered with respect to selectivity of solvents, dimethyl sulfoxide (DMSO) and dimethyl formamide (DMF). Films deposited from DMF alone at room temperature undergo rapid solvent evaporation that leads to uncontrolled nucleation and disordered quantum‐well assembly. The addition of DMSO promotes the formation of PbI_2_‐DMSO intermediates via Lewis acid–base interactions, which increase the nucleation barriers and slow down ion intercalation, thus influencing the crystallization dynamics. When combined with moderate substrate annealing, these intermediates are selectively destabilized, enabling the progressive intercalation of organic and inorganic components and more coordinated growth of quantum wells. In a research, when mixed solvent of DMF and DMSO to obtain high‐oriented 2D perovskite by employing the intermediate‐phase‐controlled crystal growth strategy, as shown in Figure 5c [74]. This results in much faster transport, nearly 50 times greater and longer carrier lifetime, and lower defect density compared to pure DMF. In another study, a quasi‐2D BA_2_MA_3_Pb_4_I_13_ was prepared by dissolving the precursors in DMF and spin‐coating at room temperature [30]. The fabricated films had considerable pinholes and were nonuniform due to rapid crystallization. However, when in the same process, when DMSO was used instead, the films were more continuous due to decreased crystallization rate. Additionally, an alkali cation was doped to obtain the desired vertical orientation. Such continuous films benefit from efficient charge conduction with minimal defects. In a separate investigation on blade‐coated 2D DJ‐based perovskite films, adding 1‐methyl‐2‐pyrrolidinone (NMP) to the DMF:DMSO mixed solvent system slowed crystallization because of strong coordination with the precursors and its low saturated vapor pressure [84]. This produced perovskite films with significantly improved crystallinity and a preferred vertical orientation. Therefore, the solvent choice must be deliberate, balancing coordination strength, volatility, and miscibility with antisolvents. Polar aprotic solvents are preferred as primary solvents because they provide sufficient precursor solubility and form stable intermediates that delay nucleation. These solvents should be combined with low‐boiling moderately polar antisolvents that can be rapidly displaced during film deposition. This controlled removal allows the main solvent to evaporate gradually to support extended grain growth and promote desired vertical orientation.

Beyond solvent engineering, spacer cation properties such as size, shape, charge, and functional group must be tuned to control the crystallization kinetics. This is especially the case when the spacers are aromatic with higher dielectric constants. Excessive incorporation of long‐chain ammonium cations can impede charge transport and overly stabilize low‐*n* phases, so the thickness (*n*‐value) and spacer content must be carefully selected and tuned [17]. However, when a small fraction of long‐chain spacers is incorporated into high‐*n* quasi‐2D perovskite films, they tend to withhold 3D‐like optical properties without disturbing the perovskite connectivity. Rather, they preferentially segregate to grain boundaries and interfacial defect sites, wherein it associates with undercoordinated Pb_2_^+^ and halide species. This localization likely delays nucleation onset, minimizes nucleation density, and contributes to the growth of larger grains. Further, fluorinated spacer engineering can modulate nucleation and growth pathways (Figure 5d,e), for improved film uniformity and reduced defect density [75]. SEM analysis reveals that Benzylamine (BZA)‐based quasi‐2D RPP films exhibit small grains with a high density of pinholes, indicating poor surface coverage, whereas difluoro benzylamine (DF‐BZA)‐derived films form denser and more uniform morphologies with enlarged grains. Further, grazing‐incidence wide‐angle X‐ray scattering (GIWAXS) analysis shows that films with conjugated films exhibit sharper and more distinct Bragg peaks accompanied by reduced in‐plane features with preferred vertical alignment. The improved ordering is ascribed to stronger intermolecular interactions of the spacer, which promote controlled crystal growth. Kelvin probe force microscopy measurements (Figure 5f) show a more uniform and negatively charged surface potential in these films compared to conventional spacers, indicating electron accumulation and improved electronic homogeneity [76].

### 2.4 Phase Distribution

The 2D perovskites formed by solution processing are typically not single phase but instead consist of multiple quantum well distribution (*n* = 1, 2, …, large *n*/3D like). From a thermodynamic standpoint, the Gibbs free‐energy differences between the adjacent high‐*n* phases are small, which leads to more uncertainty, while low‐*n* phases possess negative Δ*G*, which makes nucleation intrinsically (thermodynamically) favorable [85]. Therefore, 2D perovskites usually exhibit multiple quantum wells (MQWs). On the contrast, from a kinetic perspective, stoichiometric precursor formulations contain size‐distributed colloidal particles. The larger‐size colloidal particles settle at the bottom because of gravity, while smaller particles distribute at the top. This colloidal nature provides heterogeneous nucleation that seeds different n‐phases across the films [86].

Confocal photoluminescence (PL) mapping reveals that small‐*n* phases are enriched near the substrate, large‐*n* or 3D‐like domains dominate near the surface, and a mixed region resides in between [87]. The graphical evidence demonstrates the ordered arrangement of different *n*‐value 2D perovskite films instead of a disordered mixture. This kind of phase distribution thus produces an energy funnel that assists in charge extraction and transport in inverted PSCs. Transient absorption spectroscopy data (Figure 5g) provide complementary validation for such a distribution [77, 88]. Here, in the transient absorption measurements, the most critical signals are ground state bleaching, which are negative signals in the spectra that act as fingerprints for different layers or phases based on their thickness (*n* value). By comparing light exciting the front and backside of the film, TA data shows small n‐phases (thin layers) prefer to stay at the bottom, and that of large‐*n* tend to form near the surface. As the number of layers grows, the energetic alignment of the band edges changes, resulting in an increase in the VB and CB as *n* drops from ∞ to 1.

The improvization of mixed‐phase MQW 2D perovskites can be primarily achieved through thermal management, precursor or compositional engineering, and solvent coordination. Relative to the MQW perovskites, phase‐pure 2D perovskites, which are quasi‐2D perovskites that maintain a fixed inorganic layer (*n*) across all the perovskite slabs, exhibit lower energy loss [18]. In addition, they also offer greater long‐term stability thanks to the uniform protection provided by the spacer layers to the inorganic slabs. Therefore, the realization of such 2D phase‐pure films can be beneficial in simultaneously targeting efficiency with stability.

### 2.5 Optical Properties and Photovoltaic Implications

**FIGURE 6:**
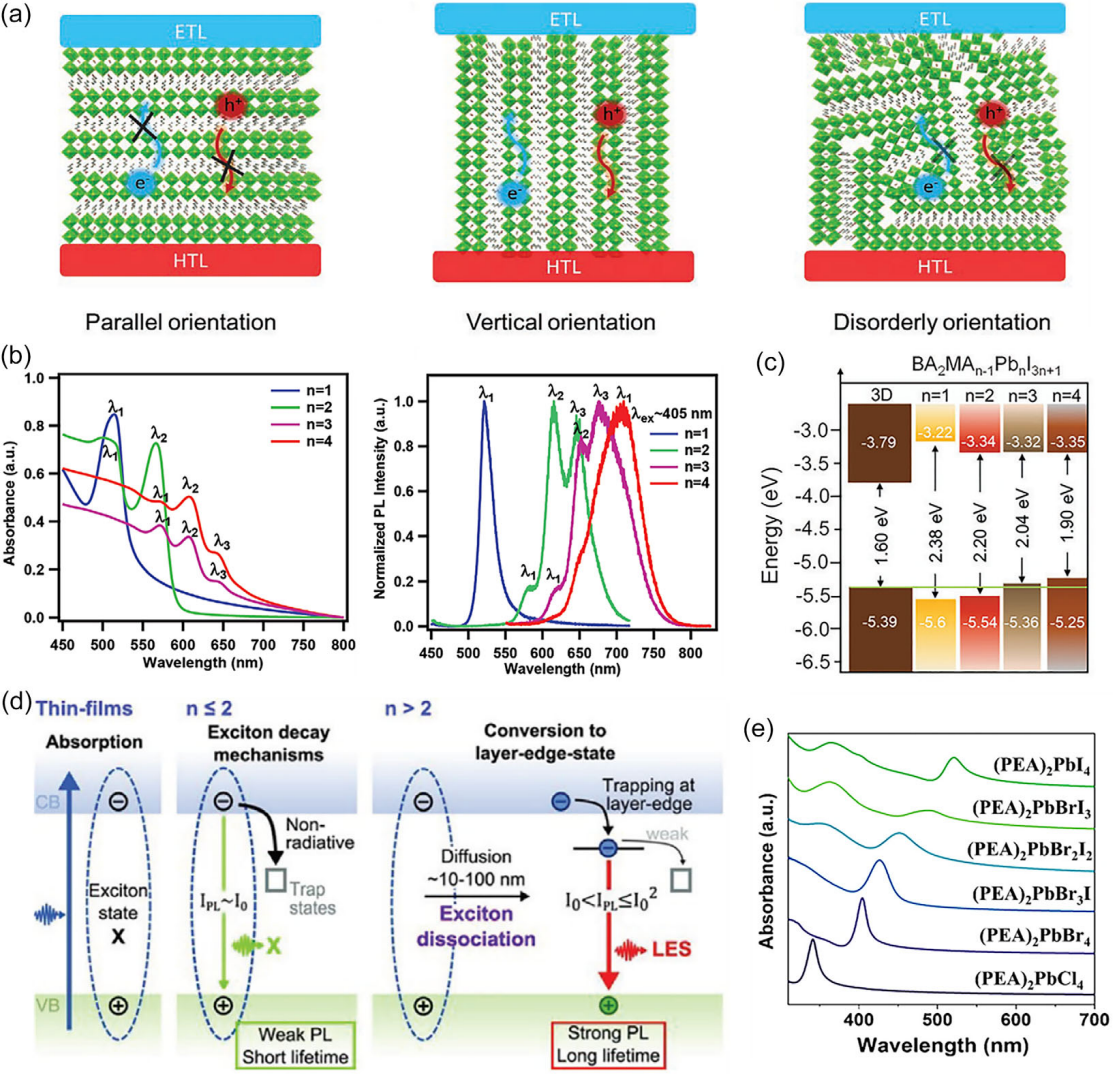
(a) Schematic illustration of orientation of crystal of 2D PSCs. Reproduced with permission [89]. Copyright 2021, Wiley‐VCH. (b) Optical absorption spectrum and photoluminescence spectrum of (BA)_2_(MA)_*n*−1_Pb_*n*_I_3*n*+1_ thin films with change in *n* values (*n* = 1–4). Reproduced from [90] under the terms of CC 4.0 license. (c) Energy level alignment of different *n* values of 2D perovskites. Reproduced with permission [12]. Copyright 2024, The American Association for the Advancement of Science. (d) Summary of main photoemission mechanism in perovskite films. Reproduced with permission [91]. Copyright 2022, The American Association of the Advancement of Science. (e) UV–visible absorption of 2D perovskite films with different halide compositions [92]. Copyright 2017, Wiley‐VCH.

As schematically illustrated in Figure 6a, the degree of preferential orientation and phase distribution impacts the transport mechanisms in 2D materials [89]. In mixed‐phase systems, coexistence of multiple orientations can therefore worsen transport anisotropy and recombination losses. Controlling both phase distribution and crystallographic orientation is crucial for translating favorable optical properties and efficient photovoltaic performance. In layered 2D perovskites, the quantum‐well thickness (*n* value) and the halide composition strongly influence the bandgap and the positions of the band edges (see Figure 6b,c), and consequently impact energy level alignment and the light‐absorption spectrum. As *n* decreases and inorganic slabs become thinner, quantum confinement intensifies, raising both the conduction‐band minimum and valence‐band maximum to higher energies and widening the bandgap [49]. When *n* grows and the slabs become thicker, the confinement energy falls, the sub‐band spacing contracts, and the bandgap narrows, bringing the band edges closer to those of corresponding 3D counterparts [12, 90]. Further, the high exciton binding energies may favor exciton dissociation (Figure 6d) [91]. Lower bandgap, higher‐n regions are favorable for photovoltaics, but they also bring greater structural and energetic disorder, which can broaden the Urbach tail and boost nonradiative recombination. Therefore, approaches that merely “mix many n” to improve film formation should be carefully assessed using optical metrics like the sharpness of the absorption edge and the amount of sub‐bandgap absorption for the long‐term performance rather than efficiency alone.

Beyond *n*, halide substitution, including mixed‐halide ratios, provides an additional degree of freedom for tuning the bandgap and optical response of 2D perovskites. Replacing iodide with bromide generally widens the bandgap and blue shifts both absorption and emission (see Figure 6e). Whereas iodide‐rich compositions narrow the bandgap and redshift the absorption edge [92].

## 3 Long‐Term Environmental Stability

The long‐term stability of 2D PSCs is due to a combination of the inherent resistance within the layered structure to environmental stressors and additional protection provided by device design modifications. Four main categories of stability considerations include moisture and oxygen resistance, thermal durability, photostability, and encapsulation.

### 3.1 Moisture and Oxygen Resistance

**FIGURE 7:**
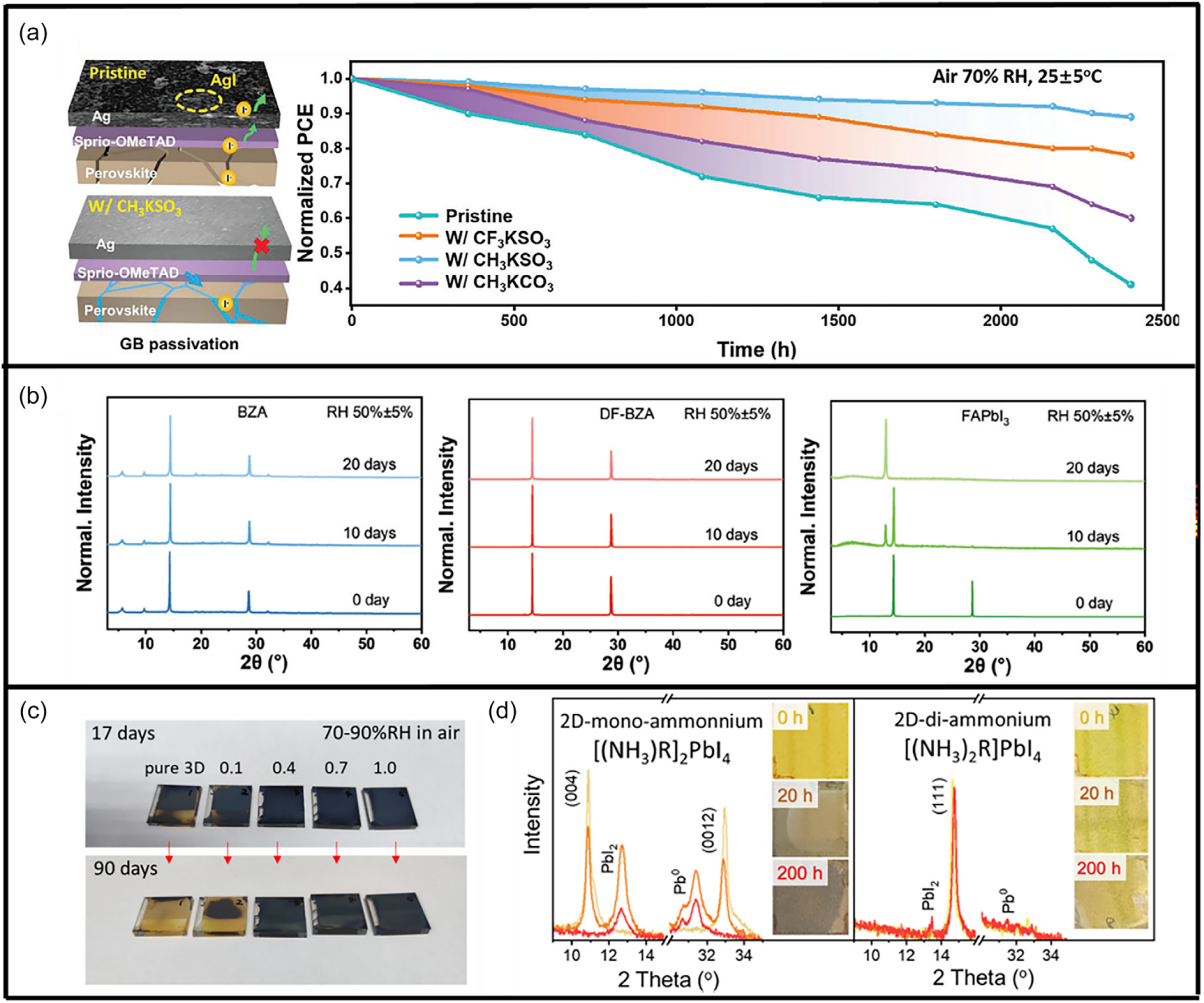
(a) Grain boundary passivation and stability in (GA)MA_3_Pb_3_I_10_ based 2D PSCs. Reproduced with permission [98]. Copyright 2024, Wiley‐VCH. (b) XRD patterns of FAPbI_3_, (BZA)_2_FA_3_Pb_4_I_13_ and (DF‐BZA)_2_FA_3_Pb_4_I_13_ films perovskite films stored under ambient exposure. Reproduced with permission [75]. Copyright 2024, Wiley‐VCH. (c) Optical images showing morphological and visual changes of perovskite films under BDAI2 spacer treatment. Reproduced with permission [99]. Copyright 2022, Elsevier. (d) XRD patterns of PEA_2_PbI_4_ and BDAPbI_4_ perovskite films under elevated temperature and continuous illumination, indicating structural evolution during aging. Reproduced from [100] under the terms of CC 4.0 license.

Perovskites in two dimensions display hydrophobic organic spacer layers, which prevent water and oxygen from entering. When PSCs are exposed to humidity, the main degradation involves interaction of water molecules with surface imperfections, especially at the grain boundaries and interfaces [93]. 2D perovskites show a more complicated degradation mechanism that is dependent on the inorganic layers number (*n*‐value), in contrast to 3D perovskites, which undergo fast hydration through formation of monohydrate and dihydrate phases. Larger‐*n* phases in 2D perovskites directly degrade to more stable *n* = 1 phase when exposed to moisture, causing disproportionation effect which initially slows deterioration but eventually causes structural collapse [94, 95]. Strong hydrogen bonds with water molecules cause organic ammonium cations to dissolve, forming volatile compounds that break off the crystal lattice. This process is further accelerated by halide vacancies, which act as nucleation sites for water intercalation and promote the disintegration of the Pb‐X octahedral framework [96, 97]. Since hydrophobic spacers are one of the significant factors contributing for the structural stability, spacer engineering is the key to enhance the stability. Introduction of potassium salts to (GA)MA_3_Pb_3_I_10_ facilitated charge transportation with grain boundaries being passivated [98]. This suppressed migration of ions and prohibited the inlet of oxygen and water leading to enhanced stability for 2400 h even at high humidity conditions (See Figure 7a). Fluorinated spacer cations further repel moisture through low surface energy and strong C‐F polarity [75]. They also facilitate better morphology and charge carrier lifetime, alongside greater moisture and thermal stability. Besides, such modification strengthens the dipole‐induced stabilization within the organic–inorganic hybrid lattice, leading to increased structural rigidity and suppression of ion migration (Figure 7b).

In Ruddlesden–Popper (RP) phases, bulky monovalent cations like phenylethylammonium (PEA^+^) form MQWs because of their bulky nature. The van der Waals gaps between layers prevent moisture from penetrating into the metal‐halide octahedral network, which enhances environmental resilience. A systematic investigation of (PEA)_2_(MA)_*n*−1_Pb_*n*_I_3*n*+1_ single crystals and thin films for *n* values ranging from 2 to 4 at high humidity levels (∼85% RH) was conducted using laser‐scanned PL imaging microscopy and time‐correlated single‐photon counting techniques. PL mapping shows that larger *n* domains directly convert into the stable *n* = 1 phase and PbI_2_ without undergoing any intermediate 3D phases, allowing them to retain their structural integrity when exposed to moisture [94]. In contrast, DJ phases employ divalent spacer cations like 1,4–butanediammonium (BDA^2+^), which directly link adjacent inorganic slabs. This results in stronger interlayer interactions and improved charge transport properties. A recent study addresses the persistent challenge of moisture‐induced degradation in PSCs by leveraging localized Dion–Jacobson (DJ) 2D‐heterostructures [99]. In this strategy, DJ‐based 2D perovskite is selectively placed at the grain boundaries of a 3D perovskite matrix, and a tailored heterointerface is formed that functions as an extremely effective hydrophobic barrier at the locations most vulnerable to moisture intrusion. Unlike conventional capping or randomly interspersed 2D–3D composites, this architecture does not obstruct charge transport, since charge carriers can still effectively move through the exposed 3D perovskite domains. This guarantees photovoltaic performance won’t suffer to promote environmental stability. Critically, operational stability testing under MPP conditions demonstrates the effectiveness of this strategy. The resulting device retained 86% of their initial PCE after 1300 h of continuous operation in an environment with 70% relative humidity (Figure 7c). The stability results from the combined effects of the tailored charge and defect topography at the interface and the DJ‐phase barrier, which stops water molecules from penetrating. Further advancement in increasing stability might require synergy between molecular design, interface engineering, and scalable encapsulation.

### 3.2 Thermal Durability

Thermal degradation in 2D perovskites follows a multistep process that begins with the decomposition of organic spacer molecules at high temperatures. Thermogravimetric analysis reveals that organic spacers typically begin to decompose at high temperatures, with aromatic spaces demonstrating higher thermal stability than aliphatic alternatives due to resonance stabilization. A key benefit of 2D layered perovskites over their bulk 3D counterparts lies in their ability to suppress temperature‐induced structural transitions that adversely affect photovoltaic performance [100, 101]. Despite this, majority of thermal stability tests have been conducted in low humidity or in N_2_ atmosphere.

Organic spacers allow for increased compliance along the interlayer axis by decoupling adjacent inorganic slabs while preserving the rigidity of Pb‐X frameworks in‐plane in hybrid lead halide perovskites [102]. The intrinsic phase stability of quasi‐2D perovskites under thermal stress significantly improved by the addition of the aromatic ammonium spacers and their fluorinated derivatives. These spacers attach advantageous octahedral tilt patterns linked to the photoactive orthorhombic or tetragonal phases and stiffen the inorganic framework through interactions, hydrogen bonding, and lattice distortion limitations [103]. Besides, the organic spacers comprising functional groups can form strong molecular interaction. Introducing sulfur–sulfur (S–S) interactions in RP phase strengthened interlayer binding via van der Waals forces and hydrogen bonding between ammonium bilayers yielding smooth pin holes [104]. These films can remain stable above 85 °C.

The anisotropic crystal lattice of 2D perovskites uses differential flexibility along in‐plane and out‐of‐plane orientations to limit heat expansion. This anisotropy reduces thermally induced strain and crack propagation within the perovskite absorber layer, producing lower coefficients of thermal expansion compared to isotropic 3D perovskites [105]. Sutano et al. used in situ GIWAXS and PL to study how the thermal evolution of 2D/3D interfaces in PSCs changes when they have 2D layers terminated with thiopene and phenylethylammonium (PEA) [42]. The *n* = 1 phase of the 2D perovskite transforms gradually into a quasi‐2D mixed phase over a prolonged period at 50 °C, without compromising the structural integrity of its underlying 3D bulk. In contrast, unprotected 3D films show a reduction of >15% in diffraction intensity under identical conditions, suggesting thermal‐induced degradation. The research findings underline the critical function of the 2D capping layer in decreasing thermally induced phase breakdown and sustaining device performance. Building on the interlayer engineering, a recent study involving the DJ series employed 1,4‐cyclohexanedimethanammonium (CDMA) cations that induce a slight displacement between inorganic slabs that tightens interlayer spacing without rigid stacking [14]. Unlike typical DJ phases with fixed alignments, this flexibility reduces the lattice strain and enhances mutual slab orientation, promoting efficient interlayer charge transport and resisting thermal distortion. To further optimize their thermal performance without sacrificing optical or electrical features, future developments in spacer engineering and lattice modulations to enhance structural rigidity without hindering transport are essential.

### 3.3 Photostability

Under continuous illumination, perovskite materials are exposed to various stressors that can have a substantial impact on their structural stability and performance. Instabilities triggered by light can be attributed to a complex interaction between various processes, including phase segregation, splitting of excitons, and photochemical reactions, especially in environments with high oxygen levels [106, 107].

Despite these inherent difficulties, substantial progress has been achieved in obtaining long‐term operational stability in 2D PSCs. BDAI_2_‐treated DJ phases suppress ion migration and photodegradation, resulting in nearly no loss after 800 h of continuous thermal aging at 60 °C, as opposed to considerable degradation in untreated RP devices [19]. PBN‐treated 2D PSCs retain approximately 88% of their initial PCE after 1000 h of continuous AM 1.5 illumination at 60 °C, in contrast to >40% loss in 3D devices within 200 h under similar conditions [108]. Maximum power point (MPP) stability measures a solar cell's sustained efficiency under continuous illumination and electrical bias, directly reflecting its real‐world performance. 2D perovskites, consisting of layered organic–inorganic structures, inherently restrict degradation routes in their 3D analogs, resulting in notably long operational lifetimes. Besides, the structural stability of different organic spacers is also a critical factor for practical applications. Notably, mono‐ and di‐ammonium spacer cations possessing similar acid dissociation constant (pKa) values, perovskites incorporating di‐ammonium spacers are markedly resistant to photo‐thermal degradation (Figure 7d) [100].

In 3D perovskites, halide ions, particularly iodide, can migrate under illumination and electric field stress, causing phase segregation and trap hydrophobic properties, interface degradation, hysteresis, and performance loss [109, 110]. PSCs deteriorate during long‐term operation because ions inside the perovskite begin to move more freely. The lower activation energy (*E*_a_) directly enables easier ionic motion. The lower *E*_a_ becomes, the faster the low‐frequency ionic relaxation time gets. This accelerates degradation under MPP, especially at low light levels. Ion migration in 3D perovskites is a thermally activated process controlled by activation energy barriers typically below 0.5 eV for iodide migration, as confirmed by impedance spectroscopy studies [111, 112]. In contrast, 2D perovskites incorporate bulky organic spacers (e.g., PEA, BA, and guanidinium) between inorganic layers, blocking ion pathways. These spacers elevate the activation energy required for halide ion migration, yielding superior MPP stability [109, 110]. The operational stability under illumination is systematically evaluated using ISOS‐L (light soaking) testing techniques, which isolate photoinduced degradation pathways from temperature and environmental stressors. ISOS‐L‐1 conditions (1‐sun illumination, 25 °C, dry atmosphere) with MPP tracking reveal that 2D‐interlayer PSCs incorporating organic structures with functional units exhibit excellent stability, retaining >85% initial efficiency after 1200 h. This is attributed to suppressed ion migration, facilitated by an increase in activation energy [113].

**FIGURE 8:**
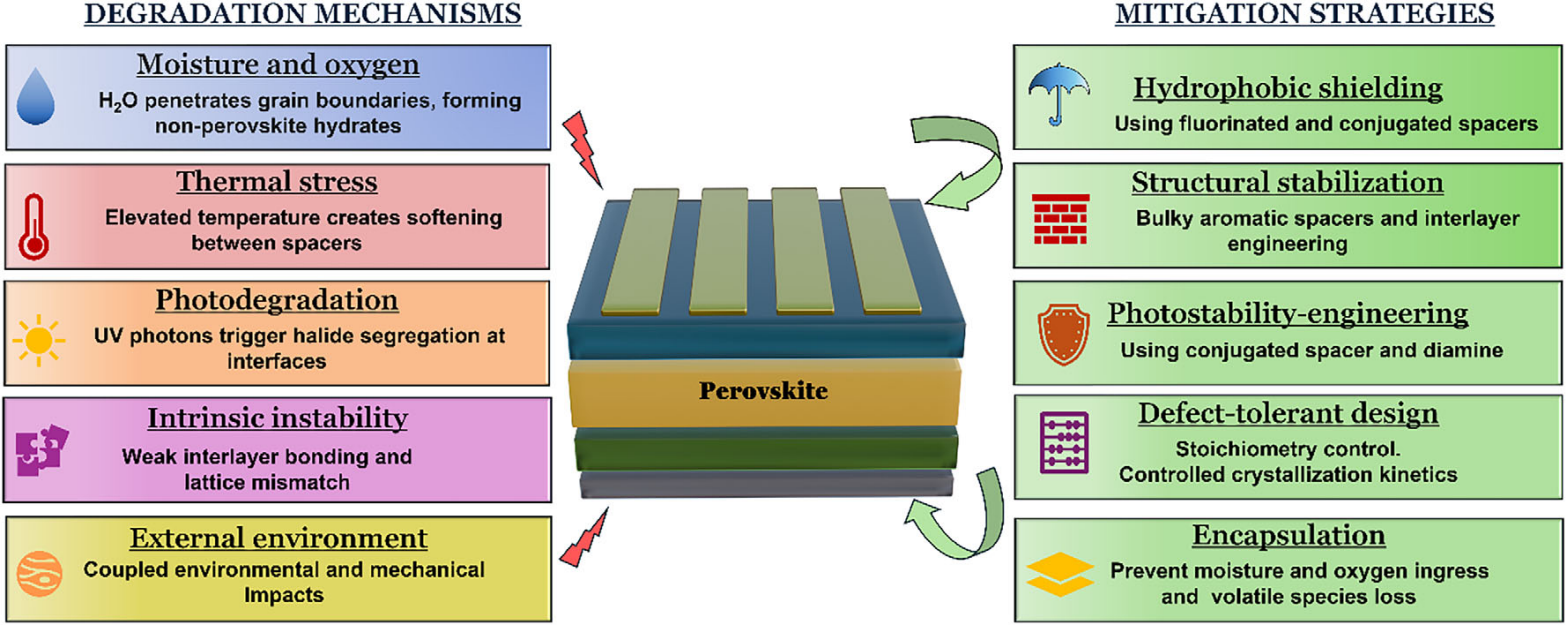
Degradation mechanisms and mitigation strategies of 2D PSCs.

Another promising approach is to integrate conjugated organic cations into 2D/3D perovskite heterostructures [45]. This can also enable large‐area modules fabrication with outstanding MPP stability, retaining over 90% of initial efficiency after 3000 h of continuous operation. The primary degradation phenomena, their underlying causes, and effective mitigation techniques are schematically presented in Figure 8.

As the perovskite absorber governs both photocurrent generation and voltage, its intrinsic stability, which is controlled by composition, dimensionality, crystallization dynamics, and defects imposes a fundamental limit on the operational device performance and lifetime [114, 115]. As organic spacers are an integral part of the perovskite structure, they play a major role behind the intrinsic perovskite stability and consequently influence overall device stability as generally reported by many literatures and is summarized in Table 1.

**TABLE 1:** Summary of the photovoltaic performance of 2D PSCs.

Device structure	*J*_sc_, mA cm^−2^	*V*_oc_, V	FF, %	PCE, %	Operational stability	References
ITO/NiO_*X*_/Me‐4PACz/(ThCH)_2_(FA_0.95_MA_0.05_)_3_Pb_4_I_13_/BCP/Ag	23.48	1.181	80.80	22.41	88% after 1500 h, RH 45 ± 5%	[26]
FTO/c‐TiO_2_/(GA)_2_MA_4_PbI_16_/Spiro‐OMeTAD/Au	22.99	1.19	81.09	22.26	94% after 1200 h, light soaking	[28]
ITO/NiO_*X*_/SA/(TTMA)_2_(MA_0.4_FA_0.6_)_3_Pb_4_I_13_/BCP/Ag	23.78	1.14	81.26	22.03	90% after 990 h, LED (100 mW cm^−2^), N2	[25]
ITO/PTAA/(FPEA)_2_FA_4_Pb_5_I_16_/PC_61_BM/BCP/Ag	22.45	1.18	79.53	21.07	97% after 1500 h, 85 °C.	[116]
ITO/SnO_2_/(GA)MA_5_PbI_16_/Spiro‐OMeTAD/Ag	22.4	1.17	80.30	21	93% after 4080 h, air.	[117]
ITO/NiO_*X*_/(GA)_2_MA_4_PbI_16_/Spiro‐OMeTAD/Ag	21.64	1.19	79.67	20.44	90% after 2000h, N2	[34]
ITO/2PACz/(PEA)_2_MA_4_Pb_5_I_16_/PC_61_BM/PEI/Ag	20.71	1.22	79.36	20.05	88% after 1000 h, 1‐sun	[108]
ITO/SAM/(PEA)_2_MA_4_Pb_5_I_16_/PC_61_BM/Ag	20.71	1.22	79.36	19.93	88% after 1000 h, 1‐sun	[108]
ITO/PTAA/(3,3‐DFAz)_2_(MA_0.95_FA_0.05_)_3_Pb_4_(I_13−*x*_Cl_*x*_)/PC_61_BM/BCP/Ag	22.28	1.11	80.26	19.85	90% after 888 h, ambient air, RH 35 ± 5%	[118]
ITO/SnO_2_/(GA)_2_MA_4_PbI_16_/Spiro‐OMeTAD/Au	21.9	1.17	75	19.30	94% after 3000 h, ambient storage	[119]
ITO/PEDOT/(DF‐BZA)_2_FA_3_Pb_4_I_13_/PC_61_BM/BCP/Ag	23.19	1.05	79.03	19.24	92% after 1100 h, white LED (100 mW cm^−2^)	[75]
ITO/PTAA/(CDMA)MA_4_Pb_5_I_16_/BCP/Ag	20.41	1.16	80.56	19.11	92% after 4000 h, RH 90%.	[14]
ITO/PTAA/(β‐FPEA)_2_FA_4_Pb_5_I_16_/ PC_61_BM/BCP/Ag	22.13	1.13	76.71	19.11	86% after 780 h, RH 35 ± 5%	[120]
FTO/TiO_2_/(DMePDA)FA_3_Pb_4_I_13_/Spiro‐OMeTAD/Au	19.09	1.19	83	18.86	85% after 1008 h, RH 85%	[121]
ITO/PTAA/(AA)_2_MA_4_Pb_5_I_16_/C_60_/BCP/Ag	20.35	1.17	78.03	18.72	100% after 1180 h, 55 °C	[122]
FTO/PEDOT:PSS/(PEA)_2_MA_3_PbI_13_/PCBM/BCP/Ag	18.52	1.20	83.39	18.48	90% after 1200 h, RH 40%	[35]
ITO/PTAA/(AA)_2_MA_4_Pb_5_I_16_/C_60_/BCP/Ag	18.57	1.25	80.03	18.42	99.9% after 750 h, RH 65%	[123]
ITO/PTAA/(AA)_2_MA_4_Pb_5_I_16_/C_60_/BCP/Ag	18.69	1.24	79.13	18.34	Nearly zero‐degradation of PCE after 800 h.	[19]
ITO/PTAA/(3BBA)_2_MA_2_Pb_3_I_10_/PC_61_BM/Cr/Au	18.22	1.23	81.2	18.20	82% after 2400 h, RH 40%	[124]
ITO/PEDOT/(MTEA)_2_MA_4_Pb_5_I_16_/PC_61_BM/Cr/Au	21.77	1.09	76.30	18.06	85% after 1000 h, light soaking	[104]
ITO/PTAA/(PEA)_2_MA_4_Pb_5_I_16_/PC_61_BM/PEI/Ag	17.91	1.22	82.40	18.04	96.1% after 8‐month storage	[125]
ITO/PEDOT/(p‐FPhFA)_2_MA_4_Pb_5_I_16_/PC_61_BM/BCP/Ag	20.54	1.13	74.55	17.37	99% after 3000 h, N_2_ filled glovebox	[126]
ITO/PTAA/(4FPEA)_2_MA_4_Pb_5_I_16_/PC_61_BM/PEI/Ag	19	1.16	79.00	17.30	93% after 500 h, RH 55%–65%	[127]
ITO/PTAA/(BA)_2_MA_3_Pb_4_I_13_/C_60_/BCP/Ag	19.86	1.24	70.44	17.26	95.5% after 2000h	[128]
ITO/PEDOT/(NpMA)_2_MA_3_Pb_4_I_13_/PC_61_BM/BCP/Ag	20.89	1.24	66.4	17.25	91% after 4100 h	[129]
ITO/PEDOT/(BEA)_2_MA_3_Pb_4_I_13_/PC_61_BM/LiF/Al	20.63	1.01	78	16.1	83.1% after 3500 h	[130]
ITO/PEDOT/(ThDMA)MA_4_Pb_5_I_16_/PC_61_BM/BCP/Ag	19.55	1.07	75.46	15.75	95% after 1655h, N_2_	[32]
ITO/PEDOT/(F‐PEA)_2_MA_4_Pb_5_I_16_/PC_61_BM/BCP/Ag	18	1.06	76	14.50	90% after 40 days	[103]
ITO/PEDOT/(PDA)MA_3_Pb_4_I_13_/PC_61_BM/BCP/Ag	18	0.97	74.3	13	100% after 100 h, 70 °C	[131]
FTO/PEDOT:PSS/(BA)_2_MA_3_Pb_4_I_13_/PC_61_BM/Al	16.76	1.01	74.13	12.52	60% 2250 h	[73]

### 3.4 Encapsulation

Encapsulation strategy functions as an external stabilization strategy applied to preformed perovskite absorbers, distinct from structural approaches that define the layered nature of the material [132]. Encapsulation with impermeable 2D materials such as hexagonal boron nitride (hBN) is one of the effective approaches in protecting air‐sensitive perovskites. hBN, a chemically inert and atomically flat 2D material, has proven particularly effective [133]. Double‐sided hBN encapsulation of phenethylammonium lead iodide (hBN/PEA_2_PbI_4_/hBN) flakes prevented degradation for at least 3 months under ambient conditions, whereas unprotected flakes degraded rapidly. Stabilization can also be achieved by directly engineering the grain boundary chemistry. Residual PbI_2_ at grain boundaries is a double‐edged sword: while small amounts can aid in passivation, excess PbI_2_ is photoactive and prone to decomposition under illumination, generating Pb^0^ and I_2_ that destabilize perovskite films. For instance, the introduction of 4‐pyridinecarboximidamidehydrochloride (4‐FAPyCl_2_) into PbI2 precursor solutions promotes the formation of quasi‐2D DJ phases that encapsulate grain boundaries [134]. This encapsulation enlarges grain size, eliminates pinholes, improves energy‐level alignment, and facilitates smoother charge extraction. Devices fabricated with this method have achieved a champion PCE of 25.32% with less than 3% loss under humid air conditions.

Strengthening of the durability has been demonstrated by in‐situ manufacturing of 2D/3D/2D double heterojunctions at both buried and top interfaces [135]. Flexible PSCs employing Ruddlesden–Popper (RP) layers interfaced with 3D perovskite films show suppressed ion migration under electrical bias, lower energy losses at both electron and hole extraction contacts, and enhanced bending durability. Moreover, the 2D perovskites at the buried interface may serve as seeds to guide the 3D perovskites crystallization process and efficiently relieve residual stress. This encapsulation method reduces light‐activated halide movement by confining migrating ions with 2D corridors. 2D layer migration controlled by mixed 2D cation passivation employing both n‐butylammonium and n‐octylammonium iodides: prolonged extended annealing promotes spacer diffusion into 3D absorber, gradually reducing the passivation and *V*_oc_. Thus, careful chain length adjustment and spacer selection strike a balance between long‐term interfacial integrity under heat and initial hydrophobic protection [136]. Moreover, 2D spacer cations like butylammonium iodide can migrate into 3D perovskite under photonic and thermal stress, overcoming interlayer energy barriers and dynamically altering the composition of the interface. Intriguingly, this migration may confer enhanced structural integrity, as the embedded cations contribute to defect passivation and inhibit nonradiative recombination under operational conditions, whereas the layered 2D phase maintains its structural integrity in dark environments.

## 4 Photovoltaic Performance Improvements

To evaluate the suitability of 2D perovskites for effective photovoltaic utilization, critical performance indicators must be evaluated in conjunction, including voltage–current properties (*V*_oc_, *J*_sc_, and FF), and operational reliability. In the context of 2D and mixed 2D/3D perovskite architectures, recent developments delineate both the progress in device performance and the inherent trade‐offs central to material and device optimization. Early 2D perovskite devices based on phenylethylammonium lead iodide delivered certified PCE <10% because of wide band gaps and poorly connected inorganic slabs that restricted photocurrent and fill factor [33].

One of the fundamental constraints in 2D perovskites is imposed by their significantly increased exciton binding energy (*E*_B_) relative to their 3D counterparts. In 2D systems, a large exciton binding energy means that at room temperature (*K*_B_ *T* ≈ 25 meV), most photo‐generated carriers remain bound as excitons rather than dissociating into free carriers [91, 137, 138]. The efficiency of charge extraction is severely restricted, since excitons must overcome substantial energy barriers to contribute to photocurrent. For pure *n* = 1 2D perovskites, electron binding energy values can reach 380 meV, making thermal dissociation highly unlikely at operating temperatures [91]. The efficiency of 2D layered perovskites is further hampered by charge transport anisotropy. As aforementioned, perovskites in 2D show a significant difference in how electric charge moves in different directions, with in‐plane carriers moving much faster than those moving out of that plane. This discrepancy, as aforementioned, mainly arises from the incorporation of insulating organic spacer layers, which impose tunneling or hopping barriers for vertical charge transport. Numerically, in‐plane resistance is approximately 10^6^ Ω, whereas out‐of‐plane resistance can surpass 5 × 10^7^ Ω in a pure 2D system [139]. Charge transport in 2D systems generally occurs through hopping between localized energy states, which results in relatively lower effective mobilities and heightened vulnerability to recombination losses, especially via transport‐assisted or nonradiative pathways [140]. Mixed‐dimensional 2D/3D architectures are often used to overcome the limitations of pure 2D classes, but they also introduce their own complexities at the interface, which can negatively affect PCE. These systems display complex interfacial behaviors that can either boost or hinder performance based on band alignment. Photophysical models have shown that 2D/3D interfaces can create p–n heterojunctions with internal potentials that are unusually high when photoexcited [43, 48]. These misalignments at the heterojunctions result in carrier blocking, consequently impeding charge transport. Effective charge extraction requires a precise match between the energy levels of 2D and 3D components. Poorly aligned bands can lead to obstacles for hole or electron movement, resulting in accumulation and higher recombination rates.

*V*_oc_ measures the highest voltage a device can produce and is directly affected by bandgap energy and the rate of electron–hole recombination. It reflects the photovoltage extractable above recombination losses. In perovskite photovoltaics, the *V*_oc_ serves as a direct electrochemical probe of quasi‐Fermi level splitting when illuminated and fundamentally reflects recombination kinetics within the absorber and its interfaces, or in other words, how quickly charges recombine, both inside the perovskite material and at its boundaries with other layers in the device [141, 142]. The Shockley–Queisser limit constrains the maximum achievable *V*_oc_ by radiative recombination, which is lower than the bandgap under AM 1.5G illumination. In theory, if radiative recombination is the only type of recombination that occurs—that is, every recombination event generates light instead of heat—then *V*_oc_ can approach very closely to the materials bandgap energy (*E*_g_). In practical devices, especially WBG layered and quasi‐2D perovskites, the *V*_oc_ often falls substantially below this threshold due to defect‐mediated nonradiative recombination and energy‐level mismatches at the transport interfaces. Still, these values remain relatively high, indicating low energy losses, suggesting most of the recombination is radiative and minimal energy and minimal energy lost as heat [143]. Recent research by Wei et al. showed *V*_oc_ values of 1.37 V for perovskites for perovskites with an energy level of 1.79 eV, exceeding 90% of the Shockley–Queisser limit, by using bromine‐substituted self‐assembled monolayer [144]. The photovoltaic performances of the 2D PSCs are presented in Table 1.

While *V*_oc_ measures the voltage potential of a solar cell under open‐circuit conditions, the complementary metric of the photocurrent output, expressed as short‐circuit current density (*J*_sc_) or short‐circuit current (*I*_sc_), quantifies the device's ability to harvest and extract photogenerated carriers under illumination. In 2D PSCs, quantum‐well structures and dielectric confinement reduce absorption at longer wavelengths, often leading to lower *J*_sc_ values compared to 3D analogs. However, advances in the optimization of phase purity, crystal alignment, and organic spacer design have facilitated *J*_sc_ values exceeding 22 mA cm^−2^ in mixed‐dimensional (2D/3D) and high‐*n* (*n* = 4,5) 2D perovskite devices [45]. Even in devices exhibiting high *J*_sc_, the curve that determines output power is calibrated by an important parameter, the fill factor (FF). The FF of a solar cell is influenced by recombination and series resistance (Rs) at the MPP, in the absence of shunts [145]. Recombination at MPP, while still occurring, becomes less significant when optimized passivation and molecular engineering are implemented. Consequently, Fill factor can be improved significantly by the suppression of resistive losses achieved through π‐conjugated cations and well‐managed interfaces, which supports a square current–voltage characteristic [146]. This correlation between *J*_sc_ and FF reveals that films that maintain strong current extraction and display long carrier diffusion lengths usually achieve higher FF due to reduced carrier density gradients and lower trap densities [147]. Herein, we outline some of the strategies to increase overall performance of the 2D PSCs.

### 4.1 Compositional Modulation

#### 4.1.1 Spacer, Cation, and Anion Design

**FIGURE 9:**
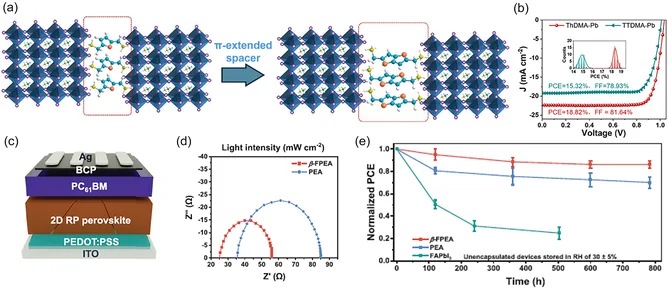
(a) Schematic illustration of 2D perovskites featuring extended π‐conjugation and (b) corresponding photovoltaic data. Reproduced with permission [76]. Copyright 2021, Wiley‐VCH. (c) Schematic illustration of 2D RP device structure, (d) electrochemical impedance spectra under dark conditions and (e) corresponding Normalized PCEs versus time. Reproduced with permission [120]. Copyright 2023, Wiley‐VCH.

As discussed in the context of the organic spacer itself, the selection of organic spacers directly controls the width and the height of the interlayer quantum‐well barriers and the dielectric screening. Large aliphatic spacers introduce thick, low‐dielectric organic layers that enhance confinement effects, which strongly restricts out‐of‐plane charge transport. Bulky aromatic spacers can improve the ordering of the structure and the orientation of the film, but they do not reduce dielectric confinement much. Because they are insulating and still limit charge transport. Spacers with reduced steric bulk or tailored π‐conjugated backbones can increase electronic coupling between layers and help carriers spread across the inorganic slabs, which partly reduces transport anisotropy (Figure 9a,b) [76, 148]. When combined with favorable crystal orientation and controlled phase distribution, these molecular design choices improve charge extraction, increasing *J*_sc_ and FF. For example, Selenophene‐based spacers delivered *J*_sc_ of 23 mA cm^−2^, and a high FF of 76.52% by combining π‐conjugation, high polarizability, and dense packing [138]. One of the effective ways to increase the perovskite PCE is to lessen the binding energy (E_B_) through strategic spacer design enhancements. For instance, ethanolamine, a high‐dielectric spacer with a relative permittivity (*ε*_r_) of 37, can decrease E_B_ by up twenty‐fold times compared to conventional standard spacers [137]. This significant decrease enables efficient thermal dissociation at room temperature, thereby fundamentally improving charge generation efficiency. Aromatic spacers with extended π‐conjugation, achieve binding energy reductions through improved electronic coupling between organic and inorganic layers [138]. The extensive π‐electron cloud associated with the selenophene ring enhances electronic delocalization at the organic–inorganic interface, thus enabling a stronger electronic interaction between the two components. Modulating the dipole moment of spacer is another pathway to decrease E_B_ and enhance charge dissociation [118]. Fluorinated spacers with significant dipole moments increase interaction between organic and inorganic layers, thereby lowering the E_B_. Moreover, fluorinated spacers are shown to promote the growth of larger crystal grains in quasi‐2D films, which minimizes recombination and extends carrier lifetimes. Additionally, the electronegative fluorine atoms contribute to surface defect passivation and enhanced film stability. Therefore, fluorination‐assisted spacer design (Figure 9c–e) improves charge transport, operational stability and improved phase purity.

Since the dielectric constant of organic spacers is considerably lower than that of the inorganic Pb–I layer, high quantum confinement effect is expected in 2D perovskites as described in Section 2.2. The substantial exciton binding energy inherent to these natural QW structures can be fine‐tuned by altering dielectric constant of the organic spacers, in particular by dielectric screening [149]. Among these, thiophene‐based perovskites possess the highest dielectric constant. In the monolayer limit, the optical dielectric constants of furan methylamine hydro (FUMA), phenyl methyl ammonium (PMA), and thiophene methyl ammonium (THMA) are 2.07, 2.14, and 2.20, respectively, increasing to 2.47, 2.60, and 2.65 for bilayer structures. Correspondingly, THMA‐based perovskites demonstrate the lowest exciton binding energy of 739 meV (monolayer) and 499 meV (bilayer), compared to PMA and FUMA. This indicates that proper incorporation of spacers with sizable dipole moments can modulate dielectric confinement and reduce the detrimental effects of exciton binding in quantum‐confined structures.

Moreover, extending π‐conjugation along the spacer, both the dipole moment and relative permittivity of the organic layer. This action lowers the binding energy while simultaneously enhancing π–π stacking rigidity, thereby improving structural and thermal stability. However, the same conjugation that enhances rigidity also adds steric bulk and can disrupt octahedral tilting patterns, which essentially reflects the molecular packing geometry that affects in‐plane transport. Comparative work on pyrene‐alkylammonium spacers with varying lengths found that only those geometries allowing favorable, near‐parallel π–π stacking with the PbI_4_^2−^ sheet result in measurable interlayer organic–inorganic orbital hybridization and exciton delocalization across multiple octahedral layers. In contrast, a chemically similar spacer with its π‐core oriented orthogonally to the inorganic layer exhibited no such hybridization and functioned as a conventional insulating quantum‐well barrier [150]. Therefore, reductions in E_B_ do not inherently translate to improvements in *V*_oc_ or FF, and these should be reported together and should be treated collectively rather than as independent results. More importantly, the effectiveness of a π‐conjugated spacer in increasing transport depends on its orientation relative to the inorganic slab, not merely its presence.

The recent study on cation‐π/π–π interactions support this interpretation by demonstrating that aromatic spacers can self‐assemble into ordered columnar stacks through cooperative cation–π and π–π interactions [151]. This self‐assembly process subsequently regulates nucleation, increases vertical crystallization, reduces grain‐boundary density, and establishes critical insight is that the device's advantage arises from self‐assembly into an ordered mesoscale structure, rather than aromaticity alone. In practice, a spacer with a suitably designed π‐system can maintain hydrophobicity and structural integrity while enhancing carrier transport, provided that its molecular packing and orientation facilitate favorable interlayer coupling with the inorganic framework. Together, these effects lead to improved charge transport and higher PCE.

The B‐site metal and the X‐site halide together determine electronic structure, carrier effective mass, and defect energetics of the 2D perovskite film. Lead (Pb^2+^)‐based 2D systems provide the highest performance, owing to their optimal band structure, strong optical absorption, and remarkable defect tolerance. However, their toxicity creates commercialization problems, prompting the search for lead‐free alternatives like copper (Cu^2+^), tin (Sn^2+^), and germanium (Ge^2+^) based systems. Copper‐based 2D perovskites offer lead‐free compositions with some favorable optoelectronic properties, such as wide band gaps suitable for tandem applications and resistance to moisture degradation. Nevertheless, their electronic structure is dominated by localized d‐orbitals, resulting in poor charge transport and severe nonradiative recombination. For example, Li et al. fabricated (C_6_H_5_CH_2_NH_3_)_2_CuBr_4_ 2D perovskite and only achieved the PCE of 0.2% [152].

Germanium‐based perovskites have potential optoelectronic properties and increased elemental abundance. Additionally, Germanium shares chemical similarities with lead and tin, with a small ionic radius of 0.73 Å. Despite this, they encounter major challenges that have impeded their experimental realization, including strong oxidation tendencies, poor material stability, and high defect densities, which directly impact device performance and longevity. The primary issue stems from the oxidation of Ge^2+^ to Ge^4+^ under ambient conditions, which causes rapid material degradation and the formation of deep‐level trap states, which severely impair charge transport, lower carrier mobilities, and hinder *V*_oc_ in solar cell devices [153]. Theoretical research has examined approaches to address these challenges in 2D Ge perovskites. For example, studies on (C_6_H_5_C_2_H_4_NH_3_)_2_GeI_4_ reveal its layered structure, adjustable bandgap, and photovoltaic promise through DFT calculations, indicating potential optoelectronic properties despite stability problems [154]. However, the practical development of high‐efficiency 2D Ge perovskites remains largely unexplored. Among the investigated alternatives, tin (Sn^2+^) has yielded the highest device performance, with 3D tin perovskites that achieve up to 14.6% PCE. In 2D tin perovskites like quasi‐2D Sn_3_I_10_ and Sn_4_I_13_, the RP structure enhances water‐oxygen stability compared to 3D MASnI_3_, though efficiencies remain below 3% due to Sn^2+^ oxidation to Sn^4+^, self‐doping effects, and nonradiative recombination [155].

Further, Halide composition also plays a multifaceted role in crystallization kinetics, defect formation, and film orientation in 2D perovskites. Partial substitution with Br^−^ or Cl^−^ not only tunes the band structure but can significantly increase the photocarrier lifetime and stability of the perovskite films. For instance, it has been shown that bromide inclusion reduces recombination in the device and improves carrier transport [156].

#### 4.1.2 Molecular and Additive Engineering

**FIGURE 10:**
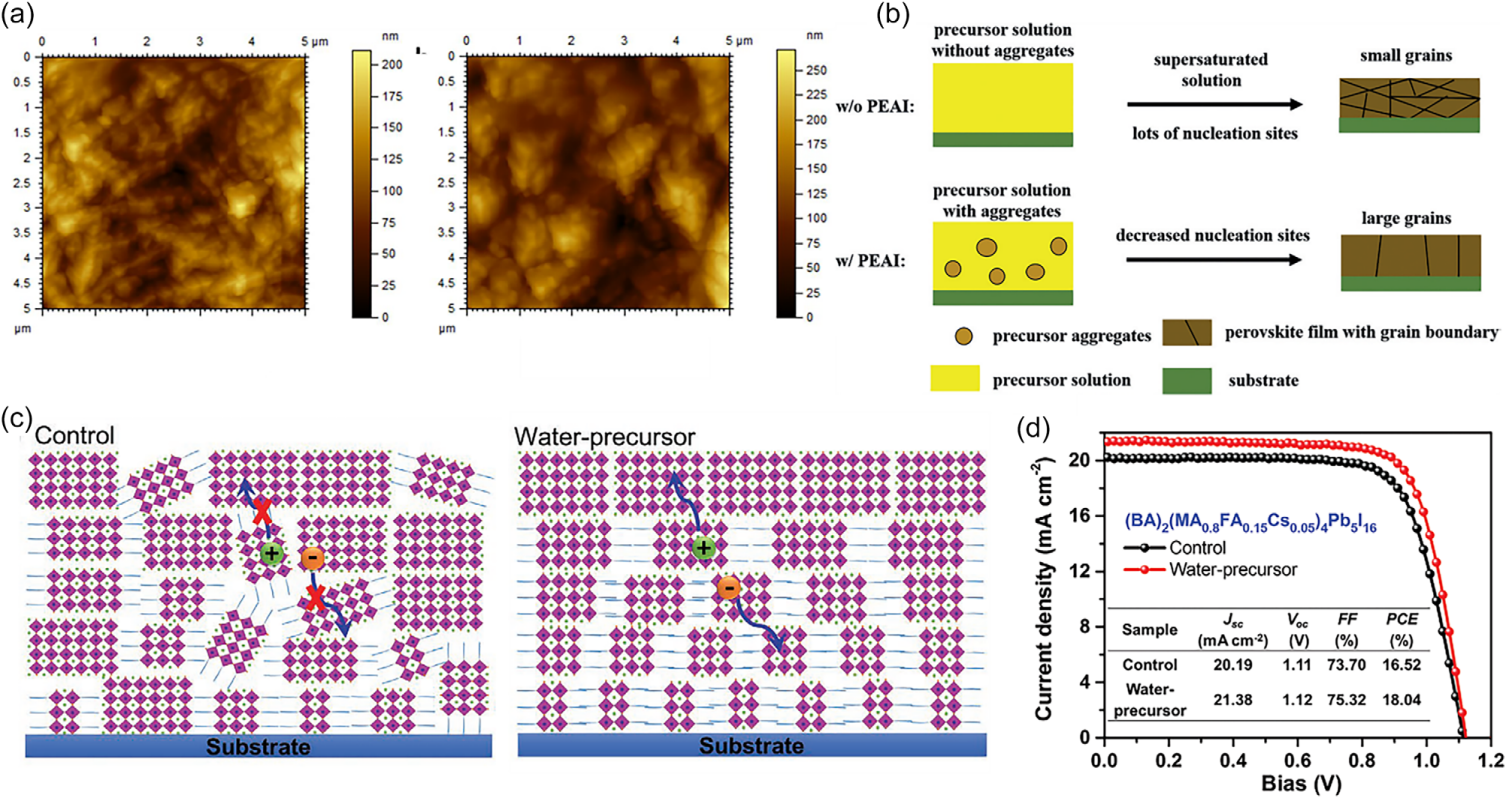
(a) Morphology evolution of 2D perovskite films with additive engineering. Reproduced with permission [159]. Copyright 2021, American Chemical Society. (b) Additive‐assisted formation of 2D perovskite films. Reproduced with permission [160]. Copyright 2019, Wiley‐VCH. (c) Phase distribution and (d) device performance in quasi‐2D perovskites. Reproduced with permission [161]. Copyright 2020, Wiley‐VCH.

Additives play a significant role in inducing vertical orientation, enhancing charge transport and thereby improving perovskite stability and efficiency of PSCs. MACl is the most widely used additive in 2D PSCs to regulate crystal orientation and phase distribution [157, 158]. MACl has been shown to promote vertical alignment along with increased grain size and reduce grain boundaries, thereby facilitating efficient charge transport and collection. The proper amount of MACl benefits 2D grain growth, whereas excessive MACl deteriorates the film quality. In contrast, the high volatile nature of MACl might lead to pinholes and defects during thermal annealing. To address this, Mao et al. found that the double additive MACl and urea synergistically decreased the nucleation rate of (GA)MA_5_PbI_16_ perovskite resulting in optimized QWs distribution and supressed recombination. The resulting PCE of the corresponding device improved from 19.01% to 20.86% corresponding to with MACl only and with combined MACl and urea additive incorporation respectively [157]. Thiocyanate salts, mostly NH_4_SCN have also been used as an additives in 2D RP perovskites, usually in combination with chlorides. The use of NH_4_SCN and HCl as coadditive led to improvement of optoelectronic features (BA)_2_(FAMA)_3_Pb_4_I_13_. With increase in coadditive concentration in films from 2.5% to 5% for NH_4_SCN and 10–20 µL of HCl, the film topology becomes more homogeneous (Figure 10a). This synergy resulted increment in PCE of 16.45% [159].

The incorporation of multiple organic spacers is also an effective additive strategy to obtain favorable optoelectronic properties of 2D perovskites by mixing different spacer cations. Lian et al. introduced the adoption of second spacer in an RP based solar cell by mixing two organic spacer cations BA and PEA [160]. They found that a stronger aggregation of the precursor solution, quantifies by dynamic light scattering (DLS) is essential for forming high‐quality films with longer crystal grains (Figure 10b). This aggregation requirement was satisfied when two spacer molecules were mixed, regardless of their size or shape, yet efficiencies above 14% were achieved only with spacers BA and PEA. Further increment to the same spacer combination was also achieved wherein sulfobetaine zwitterionic additives were incorporated to fabricate solar cells with PCE about 17.04%. Such S=O containing additive promoted uniform phase distribution and reduced competition between MA and BA during the process of crystallization due to its interaction with perovskite precursors [162].

One interesting study adopted the usually undesired water also as an additive in increasing PCE of 2D PSCs. 2 Vol% of H_2_O addition benefited both MA‐based and CsFAMA‐based perovskite with BA as spacer (*n* = 5), which reached 16.52% and 18.04% PCEs, respectively [161]. The performance gain was mainly attributed to the formation of MAI⋅H_2_O hydrate, which reduces competition between the 3D and 2D crystal‐growth pathways during hot casting and promotes vertically aligned phase components (Figure 10c,d). In summary, additive engineering in 2D PSCs has emerged as a powerful strategy to passivate defects, promote vertical crystal orientation, regulate nonradiative recombination, and increase operational stability, routinely boosting PCEs beyond 18% while enabling >1000 h retention under ambient conditions.

### 4.2 Dimensional Engineering

#### 4.2.1 Tuning the n‐Value

Increasing the *n*‐values narrows the bandgap by changing the energy of the VBM and CBM, offering a direct pathway to improve optical properties in 2D PSCs. In BA_2_MA_*n*−1_Pb_*n*_I_3*n*+1_ perovskite films, the optical bandgap drops from 2.24 to 1.52 eV when the *n* value increased from 1 to ∞, resulting in promising redshift absorption [163]. However, beyond a specific threshold effectively converts the system into a 3D perovskite, compromising the low‐dimensional structure's inherent stability advantage and necessitating careful target *n* value optimization.

The bandgap also impacted by thickness‐dependent behavior due to spin–orbit coupling (SOC). The SOC effects induce Rashba band splitting, which becomes higher with reduced film thickness [164]. Thinner, lower‐n slabs exhibit larger SOC‐induced splitting and, as a result, a higher bandgap. This means that the *n*‐value controls both relativistic band structure and quantum confinement. As *n* is increased toward intermediate values (*n *≈ 3–5), exciton binding energy decreases, the bandgap narrows, and carrier delocalization throughout the inorganic framework is promoted, which collectively improves *J*_sc_ and vertical transport. In practice, solution‐processed films rarely have a single well‐defined *n* phase, and instead they show a distribution of coexisting phases that range from low‐*n* (*n* = 1) to 3D (*n *= ∞) regimes. The effective phase distribution and its spatial organization are important in determining device performance than any single discrete *n* value. Even when a precursor solution targets a specific *n* value such as *n* = 5, the resulting film contains a wide phase dispersion, including lower *n* = 2,3,4 phases and higher *n* phases (*n* >10), introducing significant uncertainty in device. To address this issue, strategic narrowing QW distribution into high‐*n* phases while avoiding undesirable graded configurations must carried out. For instance, high‐*n* phases with minimal *n*‐value dispersion create a homogeneous energy landscape in the device made with 1,4‐cyclohexanedimethanamine (CDMA) as a crystallization‐directing spacer, greatly lowering the interphase charge transfer barriers and suppressing voltage losses with *V*_oc_ of 1.15 V and a *J*_sc_ of 23.55 mA cm^−2^. Therefore, the efficiency‐stability trade‐off in quasi‐2D perovskites is a result of uncontrolled phase polydispersity rather than an inherent material constraint [165].

#### 4.2.2 Mixing Strategy

##### 4.2.2.1 2D–3D Mixing

Integrating 2D perovskites with 3D perovskites either as a capping layer, a bulk‐in‐grain treatment, or as a small fraction of the primary absorber has emerged as a key route to simultaneously increase efficiency and stability in hybrid 2D–3D PSCs. In these heterostructures, the 2D phase typically forms at or near the grain boundaries and surfaces of 3D, where it passivates deep level defects, boost charge collection, granting high performance PSCs. Table 2 lists the device performance for highly efficient 2D/3D PSCs.

**TABLE 2:** Photovoltaic performance of 2D–3D PSCs.

Device structure	*E*_g_	*J*_sc_	*V*_oc_	FF	PCE	Stability	References
FTO/2PACz, Me‐4PACz/FAPbI_3_/MEADI_2_/C_60_/BCP/Ag	2.28	26.15	1.180	85.65	26.43	94% after 1200 h, light soaking, N_2_ glovebox	[166]
ITO/SnO_2_/FAMAPbI_3_/PyPAI/Spiro‐OMeTAD/Au	Not reported	26.25	1.186	84.79	26.41	91% after 2000 h, 45% RH	[151]
FTO/TiO_2_/SnO_2_/FAPbI_3_/4‐MeO‐PEAI/Spiro‐OMeTAD/Au	1.559	25.51	1.203	85.13	26.10	88% after 1400 h, 25 °C, 30% RH	[167]
FTO/SnO_2_/Cs_0.05_MA_0.05_FA_0.9_PbI_3_/ (A6BfP)_8_Pb_7_I_22_/Spiro‐OMeTAD/Au	1.53	26.1	1.17	85.40	26.10	85% after 1250 h, 85 °C	[168]
ITO/NiO*_x_*/Me‐4PCACz/Cs_0_._05_FA_0.85_MA_0.1_PbI_3_/(DFP)_2_PbI_4_/PCBM/BCP/Ag	1.80	25.16	1.192	86.79	26.03	92% after 2500 h, air, 45% RH	[169]
FTO/MeO‐2PACz/FA_0.9_Cs_0.1_PbI_3_/(R/S‐MBA)_2_PbI_4_/C_60_/BCP/Ag	Not reported	25.90	1.18	85.40	26	92% after 600 h, 85% RH, 85 °C	[170]
FTO/TiO_2_/SnO_2_/FAPbI_3_/PEAI/Spiro‐OMeTAD/MoO_*X*_/Cu	Not reported	25.87	1.16	86.16	25.86	90% after 500 h, 65 °C	[171]
ITO/2PACz/FA_0.85_MA_0.1_Cs_0.05_Pb (I_0.98_Br_0.02_)_3_/MAPI/C_60_/BCP/Ag	1.53	25.18	1.196	84.48	25.44	82% after 1000 h	[172]
ITO/SnO_2_/PyBA/(FAPbI_3_)_0.98_ (PyBA_2_PbI_4_)_0.02_/Spiro‐OMeTAD/Au	Not reported	25.8	1.18	83	25.30	90% after 200 h, 85 RH, 25 °C	[45]
FTO/SnO_2_/(FAPbI_3_)_0.95_(MAPbBr_3_)_0.05_/PEA_2_PbI_4_/Spiro‐OMeTAD/Au	Not reported	25.88	1.177	82.82	25.18	98.8% after 1000 h, 1‐sun	[173]

**FIGURE 11:**
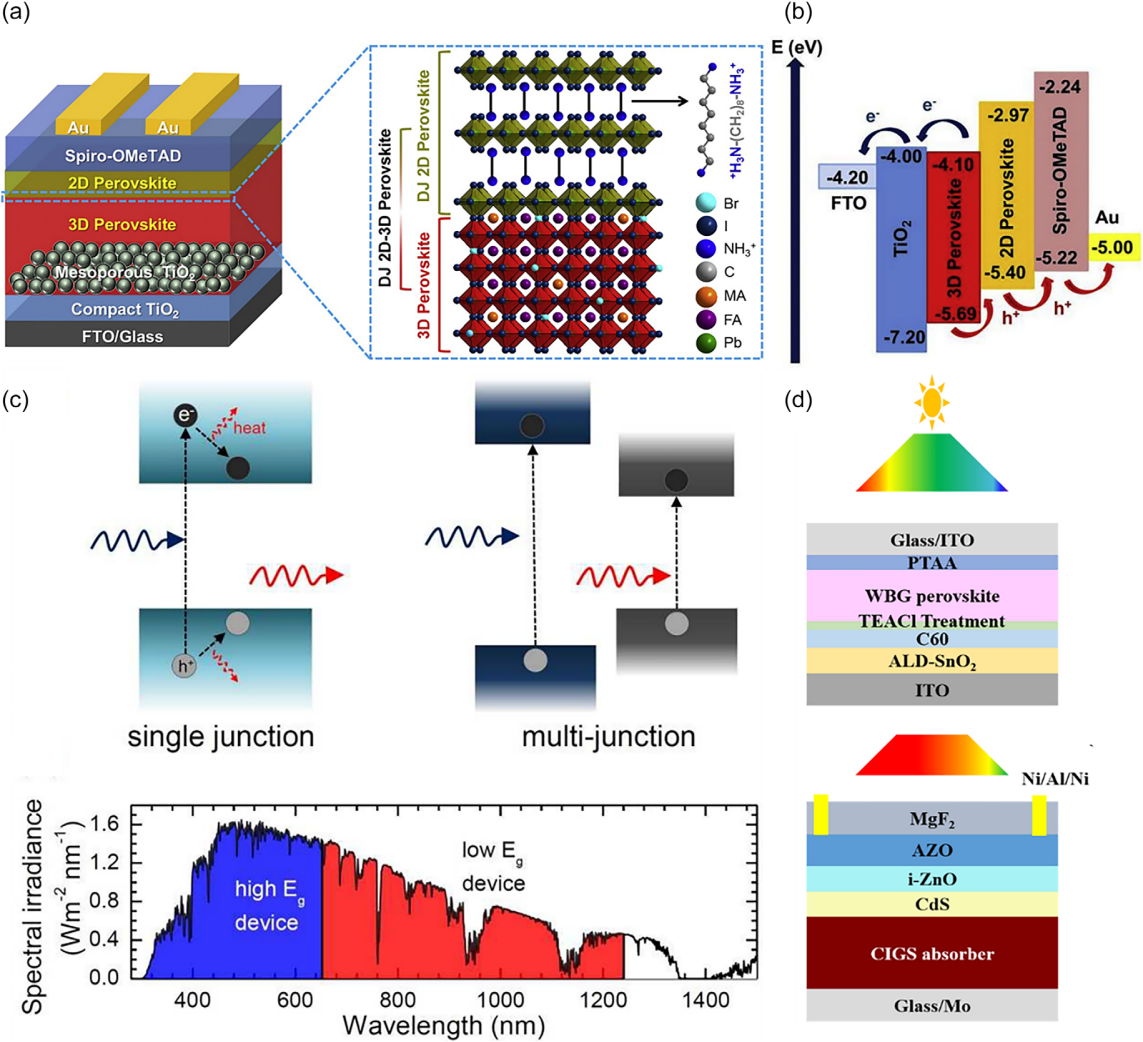
(a) Schematic illustration of the DJ 2D–3D perovskite device architecture and (b) corresponding band energy level diagram. Reproduced with permission [177]. Copyright 2020, Elsevier. (c) Schematic illustration of light absorption and energy losses in single‐junction and multiple‐junction solar cells. Reproduced with permission [178]. Copyright 2017, American Chemical Society. (d) Schematic device configuration of a 4‐terminal (4‐T) perovskite/CIGS tandem solar cell. Reproduced with permission [179]. Copyright 2021, Elsevier.

Ammonium halide is commonly used to passivate 3D perovskite surfaces, creating surface dipoles that passivate defects and reduce contact recombination, resulting in remarkable efficiency along with stability in PSCs [174]. These ammonium cations have two different interfacial behaviors, creating either an adsorbed molecular monolayer on perovskite surfaces or initiating the formation of a low‐dimensional perovskite capping layer over the 3D perovskite film [175]. Wang et al. showed that a bilayer interface‐engineering strategy can simultaneously suppress ion migration and reduce recombination by utilizing 2D capping layer phenethylammonium iodide (PEAI) [176]. PEAI when added to this framework to create 2D/3D heterojunction, it efficiently passivates surface and grain‐boundary defects while maintaining effective charge transport in the 3D material. As a result of this synergistic design, the optimized devices achieved PCE of 25.2% (1 cm^2^) with *V*_oc_ of 1.188 V and fill factor of 79.69%. Additionally, mini module (5 × 5 cm^2^) of 23.19%, with excellent operational stability, retaining over 93% of their initial PCE after MPP tracking at 45 °C for 1280 h. These enhancements can be ascribed to lower nonradiative recombination caused by improved defect passivation and more efficient carrier extraction. Adding a 2D capping layer to 3D perovskites (Figure 11a) creates a hydrophobic spacer layer that protects the absorber from moisture. A 2D octyldiammonium (ODA)‐based ODAPbI_4_ and a polypropylene glycol bis (2‐aminopropyl ether) based 2D perovskite with exceptional hydrophobicity employed as capping layers (Figure 11a) in high and low humidity environments, respectively [177]. The resultant PSCs delivered PCEs up to 21.6% while maintaining excellent stability.

The random distribution and thickness of MQWs in 2D/3D perovskite heterojunctions limit charge carrier mobility and structural stability [129]. Adding p—π conjugated, phase‐pure DJ‐type 2D perovskite interfacial layers to 3D perovskites can improve charge collection and solar cell stability. Xu et al. employed 1,1‐dimethylbiguanide hydrochloride (DMe‐bi‐GuaCl) to produce a vertically oriented pure‐phase 2D DJ (DMe‐bi‐Gua)PbI4 layer on the surface of the 3D perovskites [180]. It is found that introducing such conjugated structure with p—π bonds that overlap with the electron clouds of nearby inorganic octahedral layers leads to more effective electron arrangement. It promotes the creation of continuous vertical charge transport channels, thereby considerably improving the carrier transport.

In heterojunction devices, the value of a 2D phase depends on band alignment, interfacial recombination, and charge extraction. Theoretical work suggests that RP–PbI can be more favorable than DJ‐FAI in certain 2D/3D heterojunction configurations because of lower interfacial recombination and improved carrier separation. Considering the 3D formamidinium lead iodide (FAPbI_3_) surface and the different interfacial charge states of RP, DJ, and ACI 2D phases, first‐principles calculations examined five FAPbI_3_/2D heterojunctions (RP–FAI, RP–PbI, DJ–FAI, ACI–FAI, ACI–PbI) and analyzed their band structures and carrier kinetics before and after interface formation [56]. The simulations show that RP–PbI, DJ–FAI, and ACI–FAI all support effective separation and transport of photogenerated carriers, but RP–PbI and ACI–FAI exhibit noticeably lower interfacial recombination under the modeled conditions and thus offer greater theoretical promise for photovoltaic applications. Importantly, when carrier‐transport direction is considered, RP–PbI is predicted to be the preferred choice for conventional n‐i‐p 2D/3D devices, whereas ACI–FAI is more suitable for inverted p‐i‐n architectures.

##### 4.2.2.2 Tandem Cell Engineering

The intrinsically wide bandgap of 2D perovskites, which is a key cause of restricted light harvesting in single‐junction devices, makes them potential candidates for integration into tandem photovoltaic designs. Perovskites paired with silicon in a tandem arrangement can exploit bandgaps in the 1.6–1.75 eV range to yield theoretical limiting efficiencies approaching ~44%, a threshold far beyond what silicone alone can achieve [181].

Tandem solar cells were designed to minimize the spectral losses in single‐junction devices (see Figure 11b) [178]. In a tandem configuration, it is crucial to use a WBG subcell that not only has a large bandgap but also minimizes *V*_oc_ loss while delivering enough current to align with the bottom cell. This subcell must also remain stable when subjected to extended high‐energy photon flux and bias stress. Standard designs typically feature a WBG top subcell, often utilizing perovskite materials, paired with a narrow‐bandgap bottom subcell. The latter can employ well‐established technologies such as silicon (Si) or copper indium gallium selenide (CIGS) to boost light absorption and exceed the Shockley–Quiesser efficiency limit of single‐junction solar cells [182]. In this scenario, 2D perovskites can function as WBG top cells or, more feasibly, as passivating or stabilizing layers within quasi‐2D and 2D/3D architectures.

Pure 2D perovskites exhibit significant confinement effects, as previously discussed in Section 2.2 which inhibit ion migration and moisture ingress while simultaneously inducing excitonic effects and diminishing charge transfer in the out‐of‐plane direction. These features tend to improve photostability and environmental tolerance but comes at the expense of larger *V*_oc_ losses and limited current output when used as the sole WBG absorber in a tandem setup because nonradiative recombination at multiple organic–inorganic interfaces remain difficult to fully eliminate. Quasi‐2D perovskites with intermediate *n* values (typically *n* = 3–5) balance 2D‐induced stability with 3D‐like transport, making them more suitable as WBG absorbers in tandems [183]. Here, the alternating 2D/3D domains reduce halide segregation, while the partial 3D connectivity decreases binding energy of the exciton and increases out‐of‐plane conductivity, so the *V*_oc_ deficit and *J*_sc_ loss are generally smaller than in pure 2D absorbers, although energetic disorder at 2D/3D internal interfaces still restricts their performance in the WBG regime. In contrast, recent phase‐stable WBG 2D/3D heterostructures, where a thin 2D shell stabilizes and passivates a 3D structure, have retained the stability for more than 90% of their initial PCE after 500 h of AM 1.5 G illumination, with tandem solar cells have delivered PCE of 27% with high *V*_oc_ of 1.35 V [184]. The incorporation of large organic cation encourages the development of a 2D‐wrapped 3D heterostructure rather than a purely 3D phase. In this framework, the low‐dimensional layer functions as an interfacial barrier, increasing the energy required for ion migration and subsequently decelerating halide redistribution within the absorber. This inclination to suppress phase segregation induced by light helps in maintaining a more stable bandgap and minimizes variations in the recombination environment during the operation of the device. Tandem cells are categorized based on the connection of subcells namely two‐terminal (2T) tandems, which feature monolithic connections through a tunnel junction or recombination layer, and a four‐terminal (4T) tandems which consist of two electrically and optically independent devices that are mechanically stacked [185]. This monolithic configuration of 2T tandems imposes a critical constraint

(3) $${\text{J}}_{\mathrm{sc}}=\min\left(J_{\text{sc,top}},{\text{J}}_{\text{sc,bottom}}\right)$$

Therefore, overall tandem short‐circuit current is limited by the subcell generating the lowest photocurrent, while the total *V*_oc_ is simply sum of the individual subcell voltages which is given by

(4) $$V_{\mathrm{oc}}=V_{\mathrm{oc},\mathrm{top}}+V_{\mathrm{oc},\mathrm{bottom}}$$

This fundamental coupling generates an engineering challenge absent in 4T tandems, where subcells are electrically independent, which eliminates current matching requirements entirely. Due to the introduction of additional heterogeneity, such as surface‐versus‐bulk composition gradients and phase distribution, by quasi‐2D and 2D/3D WBG absorbers, ensuring reproducible current matching in tandems containing 2D materials is even more problematic than in single‐phase 3D WBG subcells, even when the nominal bandgap is accurately specified. One effective approach to achieve current matching involves careful selection of absorber bandgap and the thickness of the layers to maintain a balanced distribution of light among subcells. Achieving balanced light absorption is crucial for optimal current matching and is directly influential to the film thickness and bandgap of the perovskite film utilized. This strategy is essential for creating an efficient 2T tandem solar cells.

To address the constraints of standard posttreatment in methylammonium‐free WBG compositions, Wen et al. developed a 3D–to–2D perovskite conversion technique that achieved 28.1% stabilized tandem efficiencies with reduced light‐induced halide phase segregation [186]. The stability advantage of 2D perovskites is particularly essential in tandem applications, where heat and photo‐stress must be sustained over hundreds of hours. Quasi‐2D pure‐iodide perovskites particularly attractive as WBG top sub‐cells due to the inherent hydrophobicity and higher formation of energy and ion migration suppression all of which translate to synergistic long‐term stability and efficiency [187].

CIGS photovoltaic devices are efficient and stable solar cells with a narrow bandgap, comparable to crystal Si cells; they may be paired with PSCs with a 2D perovskite absorber to form tandem configurations. Incorporating 2D perovskites has improved the efficiency and stability of PSC/CIGS tandem cells (Figure 11c) by passivating electron and hole traps, inhibiting ion migration, and improving optoelectronic properties. This resulted in stable tandem cells with 24.0% PCE after 2‐thiopheneethylammonium chloride (TEACl) treatment [179]. Further, Mott–Schottky analysis indicates modified interfacial electronic properties upon incorporation of thiopene‐based 2D/3D perovskite heterojunction, validating improvement of junction quality and *V*_oc_. Collectively, the incorporation of 2D perovskites, whether as top absorbers, passivating overlayers, or crystallization‐directing intermediate phases, represents one of the impactful tandem photovoltaic techniques to maximize the efficiency.

### 4.3 Interface Optimization

Interface optimization in 2D PSCs primarily addresses nonradiative recombination and energy barriers at the perovskite/charge transport layer (CTL) junctions, which often hinder *V*_oc_ and FF. Strategic enhancements, such as passivation layers and dipole engineering have pushed PCEs beyond 20% in 2D PSCs. Buried interface engineering, particularly at the hole transport layer (HTL)/perovskite junction, has a significant impact on nucleation, crystallization, and film shape, dictating optoelectronic performance. PEDOT:PSS allows perovskite precursor fluid can spread easily on its surface due to its good hydrophilicity. However, the presence of sulfonate radicals on the surface may influence perovskite nucleation and growth. Zhu et al. used CuCl as a dopant in PEDOT:PSS, which reduced acidity and modulated its work function [188]. This method increased the wettability and inclusion of chloride in the PEDOT:PSS/CuCl composite, resulting in homogeneous morphology, vertically oriented crystal development, and better crystallinity in the coated 2D perovskite films. Xu et al. demonstrated doping Cu ions to NiO*_x_* HTL improves hole extraction and transport, leading to higher PCE in 2D PSCs [189]. The primary concern is not the overall superiority of a buried interface, but rather its ability to deliver the optimal mix of surface energy, energy‐level alignment, and defect tolerance tailored to a specific 2D phase and device geometry. Therefore, interface engineering should be considered as a strategy that integrates growth and passivation, rather than merely a surface treatment. Its effectiveness should be evaluated based on the equilibrium among crystal orientation, carrier extraction, phase purity and long‐term operational stability.

### 4.4 Emerging Techniques

#### 4.4.1 Strain Engineering

Strain engineering can be one of the powerful unifying strategies for simultaneous regulation of optoelectronic properties and operational stability of PSCs. Owing to the soft ionic lattice and relatively low elastic modulus of perovskites, internal lattice strain is readily generated during film formation, thermal annealing, phase transitions, and device operation. Residual tensile strain in perovskite thin films can lead to defect development, ion migration, and nonradiative recombination, reducing PCE and long‐term stability.

**FIGURE 12:**
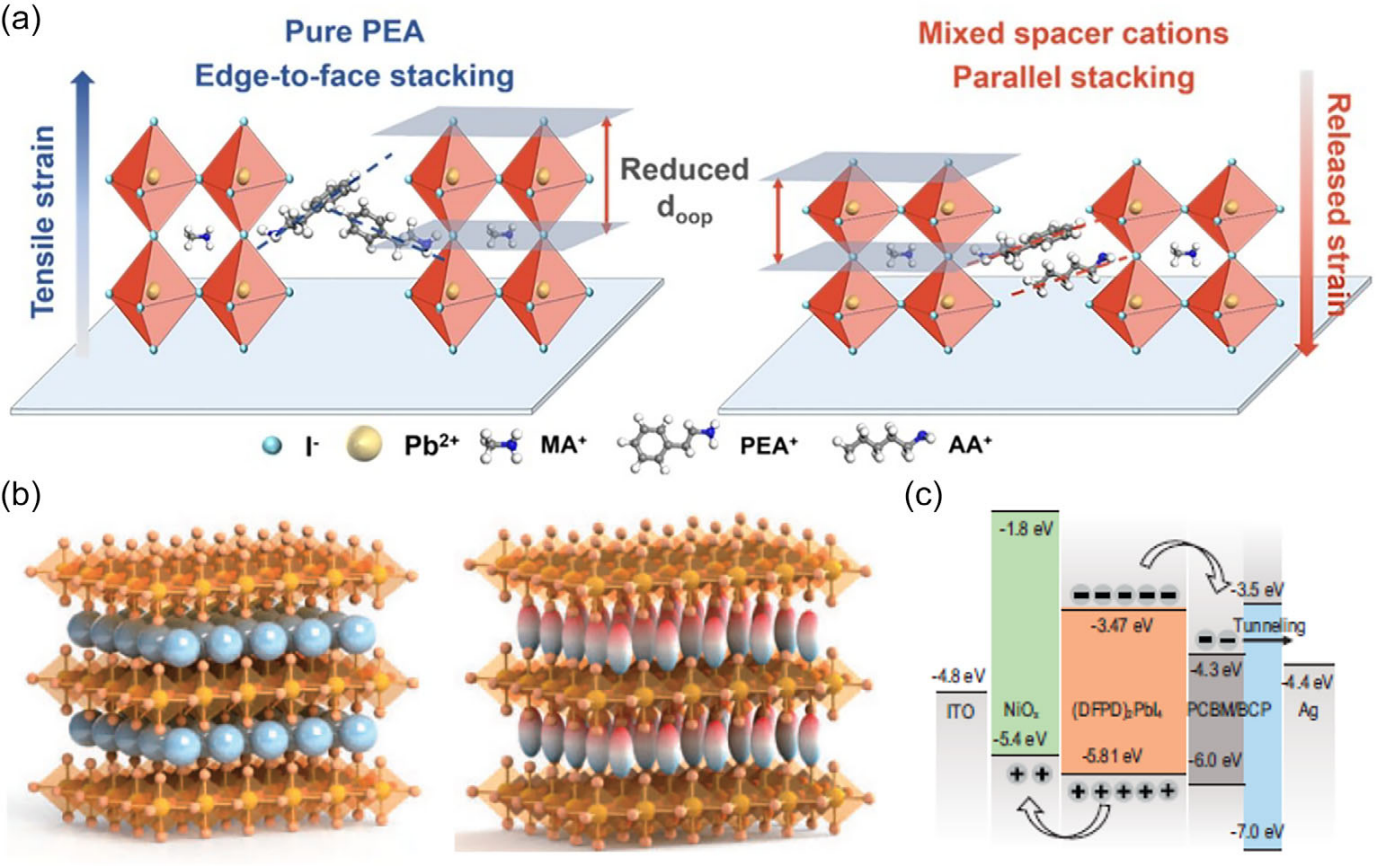
(a) Schematic illustration of strain relaxation by introduction of mixed organic spacers. Reproduced with permission [190]. Copyright 2022, Wiley‐VCH. (b) Schematic illustration comparing conventional 2D perovskite structure with ferroelectric‐modified systems and (c) schematic band structure of 2D ferroelectric solar cells. Reproduced from [191] under the terms of CC BY license.

Cheng et al. systematically investigated residual strain in 2D RPP thin films and found that organic spacers, although not part of the inorganic octahedral framework, substantially contribute to strain within the lattice [190]. Using X‐ray diffraction and peak force quantitative nanomechanical atomic force microscopy, they revealed that mismatched organic spacers may cause lattice distortion and mechanical stiffness, which often leads to increased nonradiative recombination and reduced out‐of‐plane charge transport. Importantly, strain relaxation was achieved via rational spacer‐cation modulation (Figure 12a) by partially substituting the rigid spacer with an aliphatic spacer in the spacer layer during perovskite precursor preparation. The strain‐released RPP films exhibited improved crystallinity, lower defect density, and decreased carrier recombination with a high *V*_oc_ of 1.228 V. Such structurally flexible 2D spacers help to suppress nonradiative decay caused by electron–phonon coupling by reducing mechanical stress from lattice vibrations. By replacing the rigid layer Phenylethylammonium (PEA^+^) with the flexible cyclohexyl methylammonium (CMA^+^) cation, the spacer layer acts as a mechanical damper, promoting lattice geometry relaxation and lowering carrier‐phonon scattering while maintaining chemical passivation strength. This dynamic strain‐relief mechanism resulted in suppressed nonradiative recombination with a high *V*_oc_ of 1.20 V, and PCE exceeding 25% [192].

Deliberate regulation of lattice strain, particularly the conversion of tensile strain to moderate compressive strain, can also improve efficiency and durability of PSCs. Wang et al. found that controlling lattice strain in perovskite films reduces defect production and stabilizes the crystal framework [193]. At the interface of the perovskite films, such strain regulation further manifests as a redistribution of residual stress within 2D/3D architectures, which has been shown to inhibit undesired phase transitions while promoting localized heteroepitaxial growth near the interface [194]. This behavior arises because the phase transitions in perovskites involve thermally induced bond breaking and recombination processes, which may be controlled by interaction between 3D perovskite grains and layered 2D perovskite sheets. Despite such rapid interest, strain engineering in 2D PSCs remains at a relatively early stage. Further advancements will likely depend on the cooptimization of strain regulation with dimensional control and interface engineering, enabling coordinated management of lattice distortion, defect formation, and charge‐transport pathways.

#### 4.4.2 Ferroelectricity

Ferroelectricity in 2D halide perovskites, when present, introduces an inherent spontaneous polarization, resulting in the creation of strong internal electric fields within the bulk crystals and thin films [191]. Particularly, a ferroelectric 2D perovskite layer in a device results in a photo‐ferroelectric interface, a specific area where the spontaneous polarization creates an internal electric field that combines with the intrinsic built‐in potential of the device [195]. This superimposed field effectively separates photogenerated electrons and holes, thereby decreasing their recombination probability via nonradiative routes (Figure 12b,c). This separation prolongs carrier lifetimes and increases charge collection efficiency, both factors that contributes to higher PCE. The integration of ferroelectric layers, be it within the perovskite absorber or as self‐assembled interfacial modifiers, exploits amplified electrostatic fields that enhance charge generation, separation, and collection. Mixed‐dimensional heterojunctions most potentially can benefit from interface engineering that solves the transport barriers without compromising stability through the development of high‐n‐phase 2D perovskite films that minimize detrimental interfacial effects.

Additionally, ferroelectric polarization can also improve interfacial charge extraction by modifying alignments at crucial interfaces, such as those between the CTLs and the perovskite absorber. Because of their strong chemical interactions with the perovskite film, which greatly enhance the interfacial energy level alignment, molecular ferroelectrics self‐assembled monolayers such as those based on 1‐adamantanamine hydroiodide (ADAI), can form ordered dipole layers at the perovskite/HTL interface [196]. The improved energy alignment between the perovskite and HTL mitigates the nonradiative charge recombination and enlarges the fill factor FF and PCEs. The ferroelectricity appears to manifest most effectively when favorable spacer chemistry and interlayer coupling are present. As such, they are likely to improve the overall device performance only in a supportive manner rather than as a standalone route to overcome the intrinsic problems of lower dimensionality.

## 5 Scalable Integration Opportunities

2D perovskite systems have much better operational stability compared to their 3D counterparts, with pronounced enhancements observed in stability and performance where the device function is strongly influenced by 2D phases. This link between increased 2D phase fractions and long‐term durability shows the potential of 2D perovskites to address one of the key hurdles in commercialization, even while 3D analogs continue to dominate PCE. In practice, scalable demonstrations in the current research have primarily focused on state‐of‐the‐art 3D configurations. Despite these considerations, the few but growing examples of scalable processing in 2D and 2D–3D perovskite systems offer crucial proof‐of‐concept paths, showing that their integration at a greater scale is compatible with large‐area processing conditions. In this context, this section discusses opportunities in scalable pathways in 2D perovskites‐based solar cells.

### 5.1 Hot‐Air‐Assisted Ambient Fabrication

Hot‐air‐assisted ambient manufacturing induces intermediate phase development at the air–liquid interface, allowing humidity‐resilient crystallization of 2D perovskite films. Early attempts to incorporate thermal modulation into ambient perovskite manufacturing investigated hybrid deposition systems that combined direct hot‐air flow and ultrasonic spray coating. In a study conducted by Su et al. demonstrated that post‐deposition hot‐air blowing promoted uniform nucleation and suppresses porosity, yielding pinhole‐free CH_3_NH_3_PbI_3__−__*X*_Cl_*x*_ films with controllable thickness and up to 13.5% PCE on 0.13 cm^2^ devices fabricated entirely in open‐air conditions [197]. In a subsequent independent report by Meng et al., a hot‐air assisted fabrication approach was mechanistically refined for hybrid perovskites, wherein thermal quenching induced an intermediate phase at the air–liquid interface, guiding the crystallization of vertically oriented (BA)_2_MA_4_Pb_5_I_16_ with increased structural coherence and phase purity providing 16.36% PCE [198]. Additionally, the HAA film fabrication approach is material‐efficient, humidity‐insensitive, large‐area applicable, and versatile for various 2D perovskites, making it suitable for the practical implementation of lower‐dimensional‐based optoelectronic devices.

### 5.2 Blade Coating

**FIGURE 13:**
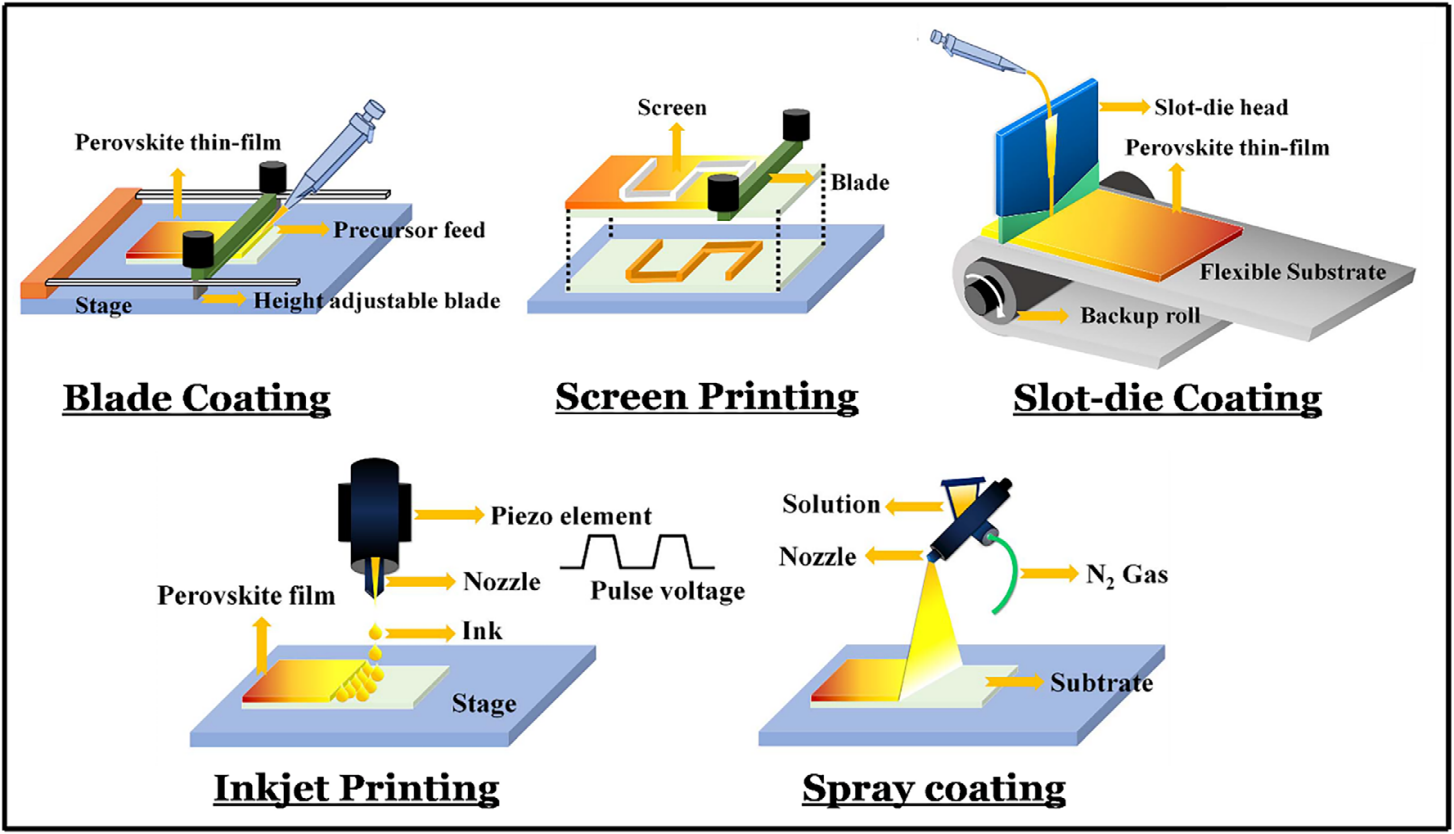
Different perovskite fabrication methods. Reproduced from [46] under the terms of CC‐BY‐NC 4.0.

Blade coating or doctor‐blade coating uses capillary and shear forces to spread the wet film by depositing a regulated amount of perovskite precursor ink between a fixed‐height blade and substrate, as schematically illustrated in Figure 13. The coating speed, ink viscosity, and blade gap govern film thickness. Solvent evaporation causes nucleation and spherulitic grain development under supersaturation conditions. This controls solvent extraction via substrate heating, gas‐knife quenching, or vacuum assistance that is necessary for preventing pinholes [199, 200].

Mixed cations (Cs/FA/MA) and halides can be used in optimized precursor formulations to adjust viscosity and crystallization kinetics while coordinating solvents create intermediate adducts that slow nucleation, yielding large grains and low defect density [201]. Blade coating, when coupled with vacuum‐assisted nucleation and interfacial engineering, can yield extremely crystalline, defect‐suppressed perovskite films even under ambient conditions. In particular, strategy proposed by Zhang et al. employ phenethylammonium iodide (PEAI) in an inverted p‐i‐n architecture to systematically apply bottom quasi‐2D passivation [202]. Their findings demonstrate that interface‐localized 2D layers can suppress nonradiative recombination and enable hole extraction without sacrificing bulk crystallinity or causing energy level mismatch. This method gets over the drawbacks of top‐surface 2D treatments in inverted structures, which frequently have *J*_sc_ losses and poor energy alignment. Low‐dimensional engineering also provides higher scalability. The quasi‐1D/2D phases generated via isomer‐specific grains by conformally bridging 3D absorber grains to the HTL reduces energetic mismatches and obstruct ionic defects. This method has been used to construct perovskite solar modules (PSMs), which have shown long‐term operational resilience and a high efficiency over 22.5% over a 6 × 6 cm^2^ area [39]. With the use of machine learning‐guided process optimization, sophisticated quenching methods, and ink formulation improvements will further reduce the lab to fab gap for blade‐coated perovskites.

### 5.3 Slot‐Die Coating

Slot‐die coating is a premetered, meniscus‐guided deposition technique where a perovskite precursor solution is fed through a narrow slit in a stationary die head and deposited as a uniform wet film on a moving or static substrate [203].

The film thickness *t* scales as *t *∝ *(γwv*/*nQ*)^−2/3^, *n* is the ink visocity, *Q* is the volumetric flow rate, *γ* is the surface tension, *w* is the slit width, and *v* is the substrate speed, following the Landau–Levich relation [204]. Through this, it is possible to achieve adjustable thickness ranging from hundreds of nanometers to microns spanning tens of centimeters [205].

For 2D perovskite inks, rheological tuning is essential. Mixed solvent systems like DMF/NMP augmented with DPSO raise the nucleation energy barrier, which amplifies wet‐film stability ≈8 min at ambient conditions [206]. This is further confirmed by dynamic light scattering measurements and TGA studies that show DPSO's coordination with Pb_2_^+^ and FA^+^ (−0.98 to −1.496 eV respectively), lowering colloid size and inhibiting premature crystallization. For ensuring continuous film deposition, the coating head formed between the upstream and downstream menisci must remain fixed to the die heads and substrate. By adjusting the slot‐die head shape (rounded corner reduction), meniscus stability is further improved [207]. Recent study shows that fully solution‐processed fabrication routes, using slot‐die coating, and blade coating can produce flexible PSCs under ambient, low‐temperature conditions [208].

### 5.4 Spray Coating

Spray coating offers a large‐scale production technique for PSMs, which uses nozzles to disperse micro‐sized tiny droplets onto a substrate. Spray‐coating has several benefits for thin‐film fabrication, including high film uniformity, controlled deposition, and compatibility with nonplanar surfaces [203]. The spray coating process for perovskite film fabrication typically involves several sequential steps. A precursor solution is first atomized to create tiny droplets, frequently with the aid of ultrasonic coaters provided with piezoelectric transducers. These droplets are then transported in the direction of a heated substrate using a carrier or shaping gas. The droplets combine and disperse to create a continuous wet film once they reach the substrate. Finally, heating the substrate and, if necessary, applying further postdeposition treatments encourage the crystallization of the perovskite material as the solvent evaporates [36].

Achieving defect‐free films depends on the droplet size and homogeneity, which are controlled by the ink viscosity, surface tension, nozzle type, flow rate, and gas pressure. Low‐surface‐tension solvents can be used to take advantage of the Marangoni effect, which increases wetting and decreases contact angle [209]. Additionally, substrate preheating encourages even spreading and evaporation. In quasi‐2D PSCs, the choice of deposition architecture, whether one‐step or sequential, further controls crystallization routes, phase purity, and interfacial control. Layered perovskite structures, for instance (PEA)_2_(MA)_4_Pb_5_I_16_ may now be deposited scalable in quasi‐2D systems via ultrasonic spray coating. Vertically oriented quasi‐2D films fabricated with such coating with PCEs more than 10% and repeatability on par with spin‐coated control could be produced by layer‐by‐layer spray deposition and nitrogen‐assisted drying [36]. Such fabricated devices exhibited long‐term stability even in large‐scale production maintaining T_77_ for 1000 h. Ultrasonic spray coating also enables the deposition of low‐dimensional perovskite capping layers to form 2D/3D heterostructures. Pneumatic spray deposition of bulky organic ammonium salts reported by Sahani et al. onto 3D FAPbI_3_ films results in uniform quasi‐2D perovskite layers (*n* = 1–2) that passivate surface and grain‐boundary defects [210]. Phase purity and spatial homogeneity throughout the coated region are confirmed by hyperspectral PL mapping, which shows that emission at 566–570 nm comes solely from 2D phase. Most importantly, this technique avoids the solvent incompatibility problems that are frequently encountered in sequential spin‐coating, allowing to accomplish full coverage and regulated thickness without causing any damage to the 3D layer.

### 5.5 Inkjet‐Printing

Inkjet‐printing (IJP) offers digitally controlled, additive, and mask‐less deposition of perovskite layers, with increasing relevance for ambient‐compatible device fabrication, simultaneously meeting the industry demands for cost‐effective strategy [211, 212, 213]. IJP relies on picolitre‐sized droplets that are released on demand from a piezo‐driven nozzle and coalesce into a wet film on the substrate surface, which ensures deterministic material placement and minimal precursor waste. This enables fine control over perovskite coverage area, which is necessary for semi‐transparent and patterned device layouts.

For a reliable inkjet deposition of perovskite layers, ink formulation, solvent engineering, and drying control need to be tailored. Optimized drying procedures after inkjet printing improve film crystallinity, shape, and eventually device performance, specifically for quasi‐2D PSCs [214]. A smoother film with fewer flaws and better absorbance comes from regulating solvent evaporation during drying process. In this context, Wilk et al. achieved vertical orientation of (4‐F‐PEA)_2_MA_4_Pb_5_I_16_ quasi‐2D perovskite films by adding 10 vol% dimethyl sulfoxide (DMSO) to the precursor ink along with a series of vacuum and nitrogen‐flushing treatments [37]. This combined solvent modulation and controlled atmosphere processing promoted superior crystallographic alignment advantageous for charge transport, yielding PCEs of 13% and 10.6% for rigid and flexible perovskite devices with an active area of 1 cm^2^, respectively.

Despite such progressive strategies in ink formulation and drying methods, reproducibility still remains a critical challenge issue in IJP PSCs, primarily because of batch‐to‐batch variations in stoichiometry control, crystallization kinetics, and film morphology. These aspects are mostly pronounced in one‐step inkjet processing, where the spatial and temporal decoupling of solvent evaporation and crystallization introduces variability in film formation, hampering phase purity [215, 216]. Hybrid two‐step procedures show that the process modularity and interfacial engineering enhance the device reproducibility. By separating precursor deposition steps and employing targeted treatments such as adding a vapor treatment in between deposition steps, these approaches reduce sensitivity to environmental fluctuations. Furthermore, to improve charge collection as well as device stability hybrid deposition techniques like spray‐assisted SnO_2_ bilayer formation have been integrated with inkjet‐printed absorber. This architecture supports ambient inkjet‐printed perovskites, providing improved recombination resistance.

Beyond architectural versatility, IJP also contributes to eco‐design for photovoltaic manufacturing. Life cycle assessment studies highlight that inkjet‐printed PSCs have a significantly lower cumulative energy demand than traditional spin‐coating, minimizing both environmental impact and the use of hazardous solvents [211]. By using green solvent‐based inks, IJP can further be made environmentally friendly. Going forward, the development of completely lead‐free, flexible, and durable 2D PSCs will depend on ongoing improvement of ink chemistries, solvent systems, and drying techniques.

### 5.6 Screen Printing

The screen printing for perovskite deposition, perovskite ink is squeezed through a patterned mesh screen using a squeegee blade [217]. The method operates on the principle of shear‐thinning behavior, wherein the perovskite becomes less viscous when shear stress is applied. This facilitates homogeneous deposition over wide substrate surfaces. The ink is forced onto the substrate surface by the squeegee pressure during the printing stroke, forming a wet film that later goes through controlled crystallization to create the final 2D perovskite layer. Because 2D perovskites have layered quantum‐well structures and bulky organic spacer cations, their crystallization dynamics are essentially different from those of 3D systems. These organic spacers affect ink rheology and solvent coordination, which calls for careful substrate temperature management and postdeposition annealing [218]. Nevertheless, the physical and chemical factors also play a significant role in determining the uniformity, resolution, and functional performance of the printed layers in both 2D and 3D PSCs. Four domains can be used to broadly classify these parameters: Printer parameters such as printing speed, pressure, squeegee shape, and angle regulate deposition efficiency, ink shear, and film thickness. Stencil characteristics including mesh count and aperture size define ink transfer behavior. Environmental conditions like humidity and temperature affect drying kinetics and defect formation. Ink properties including viscosity, solid content, thixotropic profile, drying rate, and rheology define the overall printability and wet film stability.

Reproducible perovskite films across various device architectures need careful optimization techniques for each mechanistic parameter. Cosolvent mixing can be used to control the rheology of ink. For instance, DMF: DMSO: NMP mixtures adjust wetting behavior during deposition, as demonstrated by Kulkarni et al [219]. The inclusion of an alkali metal cation, such as potassium iodide (KI), in conjunction with a polar aprotic solvent like DMSO allows for tailored intermediate stabilization and guided vertical phase segregation [218]. This approach not only regulates nucleation and slab growth during the printing process but also inhibits suboptimal phase formation and defect propagation within the bulk material. GIWAXS analysis has shown that the vertical slabs are preferentially aligned vertically and that lattice strain at buried interfaces is decreased. The results indicate that dual additive tuning is beneficial for obtaining vertically segregated perovskite phases with improved charge transport, reduced recombination pathways, and enhanced device‐level mechanical robustness. Strict control is necessary over ambient conditions, particularly humidity and oxygen levels, as low‐humidity inert atmospheres (<10% RH) increase crystallization and lower defect densities. Additionally, postdeposition annealing methods such as vacuum‐assisted crystallization and fast thermal annealing have shown promise in improving phase purity and grain connectivity [84]. Direct control of quantum well orientation has been demonstrated for DJ 2Dperovskite through the modulation of crystallization kinetics through ink chemistry and postdeposition procedures. The use of siloxane coupling agents at buried interfaces has demonstrated potential for enhancing screen‐printed film adherence and crystallinity [220].

To sum up, multiple designs must be included for screen printing to evolve as a scalable fabrication technique for PSCs. These include precision in paste formulation and rheology tuning, additive and temperature‐controlled crystallization to drive controlled crystallization and drive vertical slab orientation and facilitate charge transport, and the use of buried interfacial modifiers and adhesion enhancers to improve mechanical robustness and reproducibility.

### 5.7 Machine Learning (ML) Guided Fabrication

**FIGURE 14:**
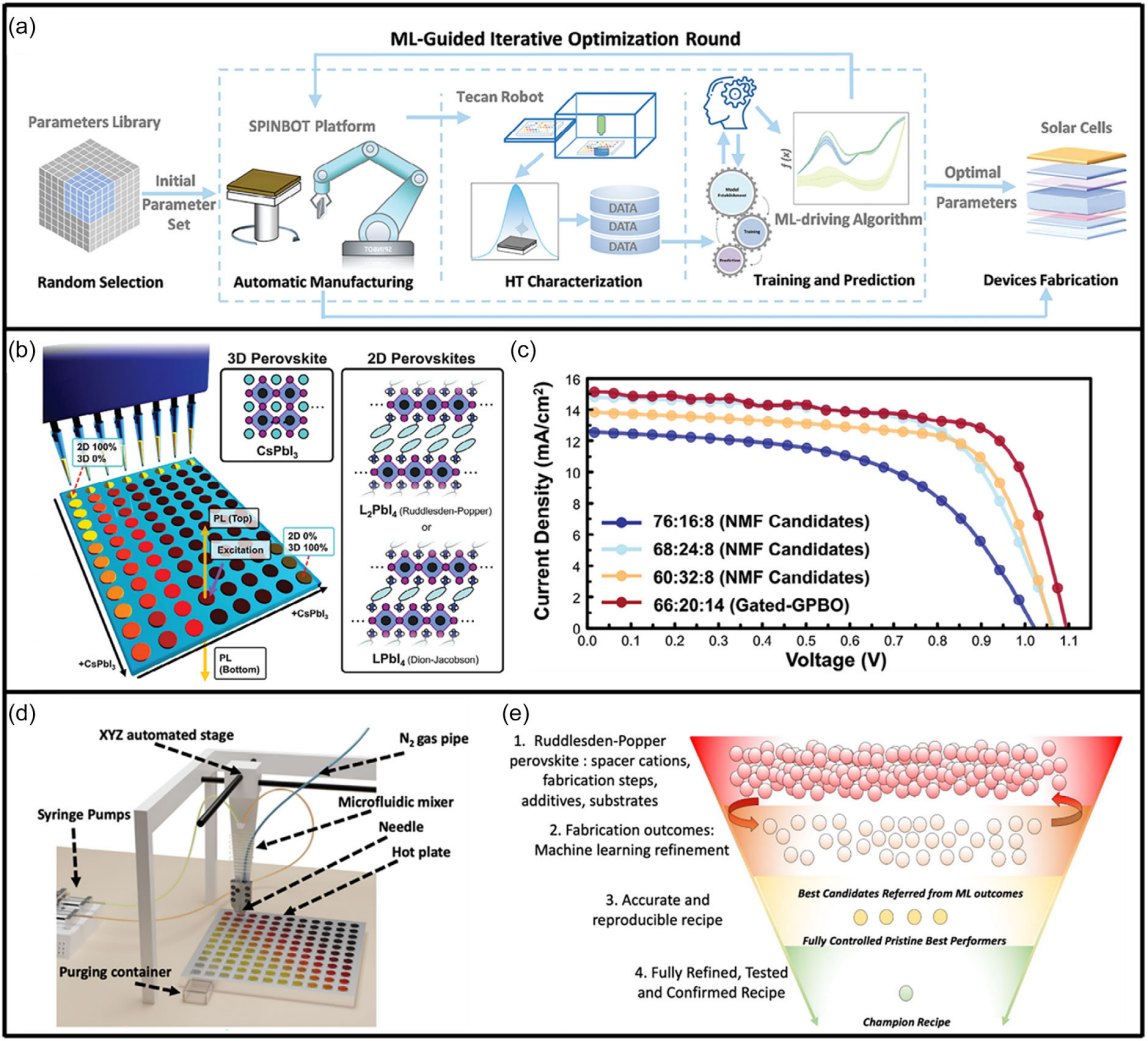
(a) Schematic diagram of a Bayesian optimization‐guided experiment workflow to achieve improved film homogeneity and reproducibility. Reproduced from [221] under the terms of CC BY 4.0 license. (b) Schematic of automated high‐throughput synthesis of quasi‐2D perovskites and (c) Performance evolution (J–V characteristics) of optimized compositions designed via machine learning. Reproduced with permission [222]. Copyright 2025, Wiley‐VCH. (d) Schematic view of key components of combinatorial high‐throughput spin‐coating free perovskite film deposition system and (e) An integrated workflow for accelerated RPP recipe optimization using machine learning with each circle signifies a batch of experiments. Reproduced from [223] under the terms of CC‐BY‐NC BY 4.0 license.

The integration of machine‐learning methods into the scalable fabrication of perovskites has transformed the field by enabling rapid materials discovery, device optimization, and process automation, all significant for the commercialization of PSCs. In contrast to conventional trial‐and‐error methods, ML uses high‐throughput data and sophisticated algorithms to speed up all stages of the production process (Figure 14a), from device creation to materials screening [221, 222, 223].

ML models trained on high‐throughput compositional libraries can anticipate the ideal spacer and halide ratios for quasi‐2D perovskites [223]. High‐throughput robotic synthesis combined with gated Gaussian‐process Bayesian optimization (Gated‐GPBO) as shown in Figure 14b allowed for the systematic tailoring of spacer chemistry in quasi‐2D perovskites. This data‐driven framework facilitated effective navigation of a discrete and highly coupled compositional landscape, allowing 480 distinct compositions to be assessed across five closed‐loop feedback iterations. Using Gated‐GPBO‐optimized quasi‐2D perovskite films obtained a PCE of 11.16% without any optimizations. The corresponding current–voltage is shown in Figure 14c. Automated combinatorial fabrication platforms combined with ML models (Figure 14d) may effectively screen large compositional and processing spaces. For example, a robotic drop‐casting framework combined with ML‐assisted design of experiments allows the investigation of thousands of quasi‐2D RPP composition (Figure 14e) by training models on a small subset of fabricated devices. Importantly, such methods eliminate reproducibility from human variability, as robotic microfluidic precursor preparation and deposition guarantee uniform film creation across big datasets.

Random forest and Gaussian process regression models have delineated critical compositional descriptors such as spacer dipole moment and ionic radius correlating strongly with phase purity of film and stability. Transfer learning techniques use DFT‐computed formation energies to further extend predictions to new cation combinations, allowing for the identification of promising compositions before synthesis [224, 225]. Embedding machine learning into diffuse‐reflectance and in situ PL of blade‐coated films enables real‐time quality evaluation. Dynamic PL video data can be fed into deep learning models to detect minute morphological changes, helping optimize parameters like gas‐knife quenching in real time [225, 226].

High‐throughput robotic fabrication platforms, when integrated with ML‐driven feedback, further accelerate material and process discovery. Liquid‐handling robotics executed thousands of drop‐cast or inverse temperature crystallization reactions, with machine learning models and algorithms autonomously guiding exploration towards previously untested compositional regions, reducing optimization cycles [227]. This closed‐loop approach significantly reduces the number of iterations required to identify stable perovskite phases compared to manual trial‐and‐error.

The automated fabrication method greatly minimized variations in the performance of the photovoltaic materials compared to manually produced materials. However, moving from lab demonstrations to scalable manufacturing, confirming cross‐compositional transferability, and conducting comprehensive cost–benefit analyses are essential unresolved challenges that future research needs to address. As the field advances, Data‐driven frameworks have the potential to provide deeper chemical insights and direct the logical design of stable, high efficiency devices.

### 5.8 Vapor Deposition Techniques

**FIGURE 15:**
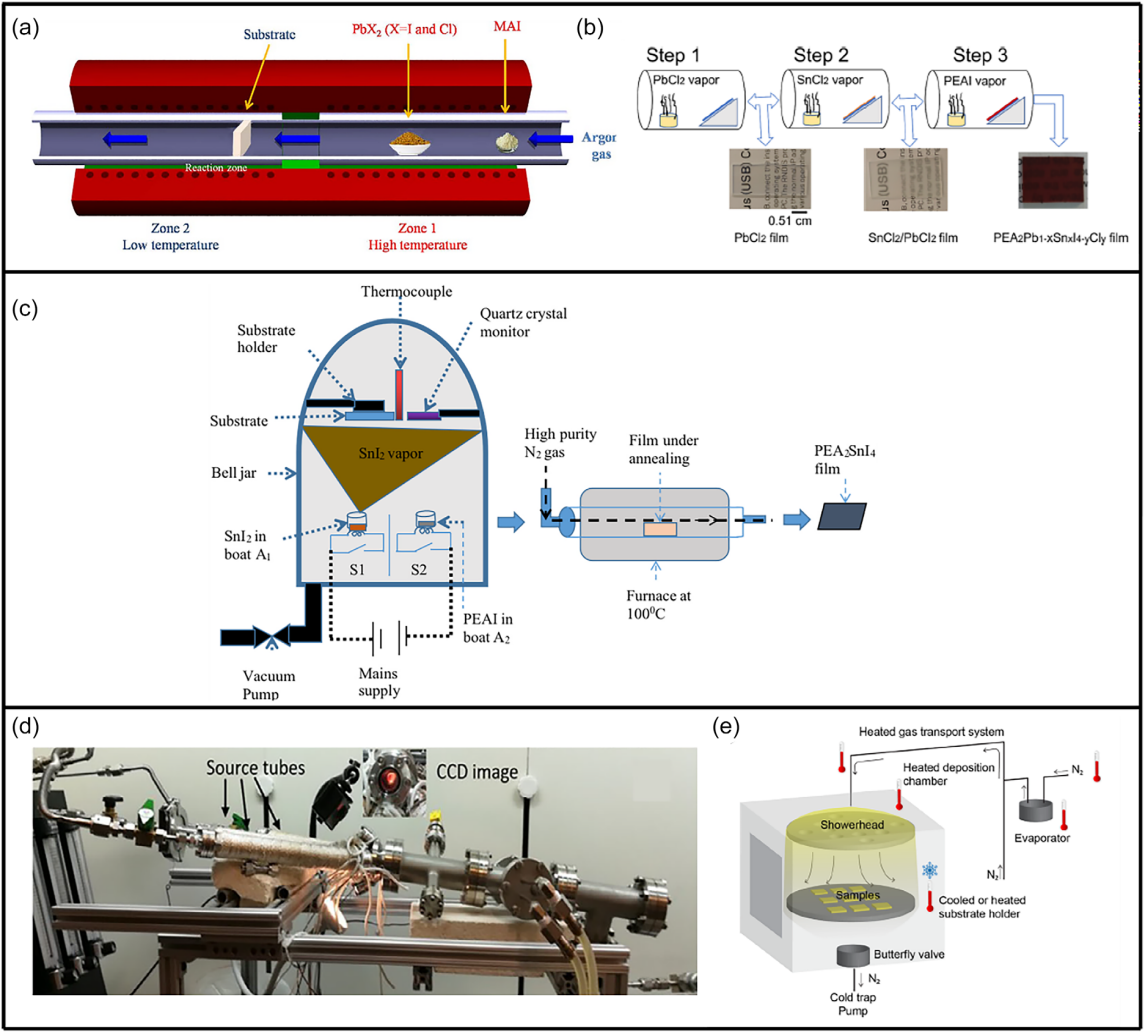
(a) Schematic illustration of one‐step HCVD process. Reproduced from [228], CC BY 4.0 license. (b) Schematic representation of sequential vapor deposition workflow. Reproduced from [229] under the terms of CC BY 4.0 license. (c) Vapor‐assisted growth of layered perovskite thin films. Reproduced from [38] under the terms of CC‐BY‐NC 4.0 license. (d) Photograph of a horizontal vapor transport deposition setup. Reproduced from [230], under the terms of CC‐BY‐NC‐ND 4.0 license. (e) Schematic of vapor transport deposition setup. Reproduced with permission [231]. Copyright 2021, American Chemical Society.

Chemical vapor deposition (CVD) is an industrially appealing method for producing high‐quality perovskite thin films on a large scale, including 2D perovskites for photovoltaic applications. CVD procedures for perovskite film fabrication usually take a one‐step or sequential approach (Figure 15a,b) [228]. In one‐step CVD, all precursors, organic or inorganic, are simultaneously introduced into the chamber either as a gas or vapor. Sequential CVD involves first depositing an inorganic precursor on a substrate, followed by exposing it to organic halide vapor like PEAI to change the metal halide layer into the perovskite phase at moderate temperature [229, 232, 233]. For layered perovskites, careful management of precursor sublimation rates and temperature gradients is critical due to differing volatilities and sublimation temperatures of the organic and inorganic components. Modified CVD processes have boosted the film uniformity and scalability. Hybrid CVD extends traditional CVD by allowing for simultaneous control over substrate conditions, interfacial engineering, and vapor‐phase precursor chemistry [234]. This integrated method achieves improved film quality, phase purity, and interface alignment. Wang et al. presented a hybrid method that incorporates a solvent‐assisted recrystallization stage with traditional vapor deposition. Many small grains become fewer but much larger grains due to the intermediate recrystallization phase that occurs only at the grain boundaries. This grain boundary elimination method substantially reduce the film defect density and consequently improved device longevity [235]. Notably, CVD can also be applied to lead‐free or lead‐reduced compositions, increasing the possibility of stable and environmentally friendly device topologies. Furthermore, because of industrial scalability, the technique shows compatibility with the manufacturing of tandem and large‐scale area perovskite modules. Despite these upsides, CVD‐based perovskite films can occasionally fall short of their solution‐processed counterparts in terms of optoelectronic quality, especially when it comes to carrier lifetimes and bulk crystal quality. To further reduce nonradiative recombination and defect states, recombination and defect states, research is still being carried out on enhancing interface engineering, postdeposition treatments, and heterostructure formation. Table 3 provides the summary of benefits and challenges associated with different fabrication methods for coating perovskite films.

**TABLE 3:** The advantages and limitations of various fabrication methods.

Fabrication technique	Advantages	Limitations
Hot air assisted	Humidity‐insensitive, reproducible in ambient air	Temperature optimization for uniformity at larger areas
Blade coating	High material utilization, low‐cost, widely used to build 2D/3D heterostructures	Meniscus‐driven convection and drying kinetics are harder to control for anisotropic 2D crystallites
Slot‐die coating	Roll‐to‐roll compatible in ambient condition, low solvent waste	Film uniformity
Spray coating	Enables layer‐by‐layer sequential deposition of different dimensionality, ambient/humid tolerant	Overspray/material loss
Inkjet printing	Contactless, maskless, low‐cost, and low‐material consumption	Potential nozzle clogging, slower throughput
Screen printing	Cost‐effective, pattern flexibility, reproducibility	Limited with low viscosity inks
Chemical vapor deposition	Large‐area scalable, uniform films	High‐material consumption
Physical vapor deposition	Uniform and dense films, solvent‐free	High‐material consumption
Vapor transport deposition	Uniform and dense film	Not yet broadly validated for 2D PSC absorber

Physical vapor deposition (PVD) is another scalable vapor deposition method which can be used for PSC fabrication. In PVD, precursor materials whether organic, inorganic, or a mixture, are physically evaporated or sputtered under vacuum and then condensed directly onto a substrate to produce dense, minimal‐pinhole perovskite layers. A major advantage of PVD is its solvent‐free, maskless, and contact‐free procedure [236].

2D PEA_2_SnI_4_ perovskite thin films fabricated by sequential PVD (Figure 15c) facilitate controlled formation of uniform films with tunable thickness and optoelectronic characteristics by regulating parameters like substrate temperature and precursor deposition rate [38]. FESEM micrographs of these deposited films demonstrate they have minimal pinholes and densely packed grain sizes essential for reducing recombination losses in devices. Despite these benefits, several challenges hinder large‐scale implementation of PVD for perovskite thin‐film fabrication. Coevaporation of volatile organic precursors like methylammonium or phenylethylammonium halides requires precise control due to their differing vapor pressures and thermal stability. Additionally, it is still crucial to maintain constant deposition rates and good precursor purity because even slight variation can impair device performance, stoichiometry, and film uniformity [237].

Alternatively, to overcome these issues, alternative vapor‐phase processes have gained attention. Among these, vapor transport deposition (VTD) presents a technically promising route to scalable, reproducible, and controllable thin‐film deposition method. In VTD processes, the evaporated precursors are transferred from the sources to the substrates (Figure 15d,e) using a carrier gas [230, 231]. Depending on the specific material system, optimal conditions vary. VTD has an operating pressure in the hundreds of Pa range, which is orders of magnitude higher than other deposition processes. As a result, a simpler pumping system is needed [238]. Several variables, including carrier gas flow rates, source‐substrate flow rates, chamber pressure, and precursor material temperatures, must be carefully controlled during the process [239]. To date, VTD has been primarily used for 3d perovskite film fabrication, and its application to 2D perovskites remains an open area for future investigation. The intrinsic environmental stability of 2D perovskites and absence of residual solvents in VTD potentially increase device durability making it compatible for long‐term photovoltaic operation. Future efforts should focus on reactor scale‐up, in situ diagnostics for real‐time process control, and exploration to lead‐free 2D perovskite compositions, leveraging VTD's versatility and high throughput.

## 6 Perspectives

2D perovskites although capable of combining high stability with good efficiency, yet there are few tightly linked bottlenecks that must be addressed before technologically meaningful deployment. In this perspective, we discuss selected challenges, emerging strategies, and future directions across the materials, device and application levels of 2D perovskite photovoltaics.

### 6.1 Fundamental Understanding and Material's Design

#### 6.1.1 Collective Control of Structure–Property Relationships

Despite undeniable stability gains, 2D perovskites confront multifaceted bottlenecks. Enhancing stability often comes at the cost of weakening the photovoltaic design primitives that 3D perovskites so effortlessly invoke like low exciton binding energies, isotropic transport, and well‐defined phase distributions. However, attempts to directly translate optimization strategies form 3D to 2D perovskites have yielded limited success, reflecting the fundamentally distinct quantum‐well thickness (*n*‐value) and crystallographic orientation collectively define a coupled. Progress in 2D perovskite in photovoltaics will therefore depend not on incremental gains in power conversion efficiency alone but treats dimensionality, spacer physics, and crystallization pathways as a unified design framework rather than a collection of properties.

#### 6.1.2 Ion Migration

While reduced dimensionality and stronger organic–inorganic interactions can often suppress ion mobility to some extent, several key issues still persist. Halide ions (I^−^, Br^−^) and smaller mobile cations (MA^+^, FA^+^) can still migrate under electric fields, illumination, or even thermal stress. Therefore, a deeper mechanistic understanding of ion migration in 2D perovskites is essential. Advanced characterization such as operando spectroscopy and time‐resolved microscopy combined with atomistic simulations will clarify migration pathways. The design of spacer cations with tailored dipole moments or steric hindrance may further suppress ion transport.

#### 6.1.3 Lead‐Free 2D Perovskites

The question of lead content remains an important consideration in the long‐term deployment of 2D perovskite technologies. While layered architectures and hydrophobic spacer cations can mitigate moisture ingress and reduce lead leakage under certain conditions, the total lead content per functional device is still comparable to that of 3D perovskites for similar absorber thickness. Hence employing encapsulation strategies to prevent lead leakage, exploration of mixed‐metal perovskites (Sn–Pb, Ge–Pb) that lower total lead content while maintaining stability are few of the alternatives that minimize the exposure.

### 6.2 Device Translation and Emerging Opportunities

#### 6.2.1 Indoor 2D Perovskite Photovoltaics

The rapid advancement of Internet of Things (IOT) ecosystem and the increasing use of smart home technologies have generated a major demand for small and maintenance‐free energy sources for electronic devices. Due to their short lifespans and high maintenance costs, traditional batteries are not preferred for many IOT nodes, such as wireless transmitters, environmental sensors, and processors which operate in the microwatt domain. Customized indoor photovoltaics (IPV) cells have emerged as an attractive option in this context. In contrast to outdoor photovoltaics, IPVs must work under artificial light sources like fluorescent lamps and light‐emitting diodes. These sources exhibit narrow spectral distributions primarily confined to the visible region. Extensive analyses indicate that the optimal absorber bandgap for IPV lies between 1.7 and 1.9 eV. Besides 3D perovskites, 2D perovskites have also attracted growing attention for indoor photovoltaic applications. The layered quantum‐well structure of 2D perovskites offers numerous inherent benefits, including suppressed ion migration, enhanced environmental stability, and highly adjustable characteristics. In particular, by varying the number of inorganic octahedral layers (*n*), the effective bandgap of 2D perovskites may be accurately controlled, allowing alignment with the ideal IPV bandgap range of 1.7–1.9 eV under visible light illumination. Despite these benefits, the application of 2D perovskites in the IPV also introduces new constraints related to charge transport, phase distribution, and scalable processing, that need to be carefully considered.

#### 6.2.2 Flexible 2D Perovskite Photovoltaics

Flexible 2D PSCs leverage the intrinsic mechanical compliance of layered structures to address the conventional perovskites to microcrack formation under bending or cyclic deformation. This is particularly relevant for wearable electronics, building‐integrated photovoltaics, and portable power systems. By facilitating stress dissipation through interlayer sliding and deformation, the van der Waals or hydrogen‐bonded organic spacer layers between inorganic slabs slow the spread of cracks and improve the absorber's mechanical robustness. However, this mechanical advantage sometimes is overestimated if considered in isolation, as flexible functioning ultimately depends on the weakest mechanical link in the device stacks, including interfaces, electrodes, and substrates. Despite their promising mechanical properties, pure 2D perovskites have lower carrier mobility with higher binding energy than 2D counterparts, which together limit device efficiency and their competitiveness in single‐junction photovoltaic applications. These trade‐offs suggest that the mechanical advantages of 2D materials come at the expense of a more complicated electrical environment, where improvements in flexibility and stability must be balanced by careful optimization of charge transport. The 2D/3D heterojunction framework attempts to reconcile these opposing demands by combining a high‐mobility 3D bulk with a thin 2D or quasi‐D surface layer that simultaneously passivates defects and improves flexible devices to withstand tensile and bending deformation. While this method has made it possible to boost efficiency and stability, it also creates new difficulties for phase control, interfacial homogeneity, and thickness optimization since thicker or nonuniform 2D capping layers can raise optical losses and series resistance. Even when the perovskite layer is mechanically strong, flexible devices are vulnerable to failure at interfaces and electrodes, where stress accumulates and brittle materials such as indium tin oxide are incompatible with repeated bending.

Future developments should therefore concentrate on system‐level approach where the electrical and structural characteristics of 2D and 2D/3D absorbers are explicitly cooptimized with mechanical design of substrates, transport layers, electrodes, and encapsulation. This includes the integration of flexible, conductive, and chemically stable interfaces and phase‐engineered quasi‐2D designs that spatially separate high‐n domains for effective transport from low‐n regions at surfaces and grain boundaries for passivation.

#### 6.2.3 Reversibility

An emerging opportunity in 2D perovskite is that controlled exposure can induce reversible structural transformations without compromising the integrity of the perovskite network. From a photovoltaic perspective, such reversible has great potential to offer a pathway toward devices that are not only resilient to environmental fluctuations but also can leverage to extend operational lifetimes. In this way, reversibility is not merely a safeguard against degradation but can be engineered for multifunctionality.

#### 6.2.4 Model‐Driven Design

ML represents a new frontier in perovskite photovoltaics, offering predictive design and optimization of devices. With the use of machine learning algorithms, hybrid Monte Carlo simulations are able to capture system sizes that are much above the capabilities of traditional first‐principles techniques. These approaches reveal universal trends such as the quick transformation of homogeneous single‐phase QW structures into desired QW structures into mixed‐phase distributions, which is further fueled by the thermodynamic preference for low‐n layers and the high affinity of spacer cations for the inorganic MX_4_ framework. Beyond fundamental design, machine learning is emerging as a powerful accelerator for device translation of 2D perovskite, as aforementioned in the previous section. The ML‐optimized 2D perovskite films are reproducible and involve an automated fabrication process with rapid optoelectronic characterization. This allows a scientifically controlled pathway to accelerate discovery, reduce trial and error, and connect materials discovery directly to device performance.

## 7 Conclusion

2D perovskites, which are now at the forefront of photovoltaic research, combine structural robustness with efficiency, making them strong candidates for integration in next‐generation solar cells. In this review, we first examined their layered structures and their different classes, followed by discussion on critical performance determinants, including electronic structure, crystal orientation and its optimization, and bandgap properties. Next, we summarize the stability improvement pathways for 2D perovskites. We then see diverse strategies to improve the performance of solar cells. These strategies include component modifications, dimensional engineering, interface optimization, and some emerging techniques such as strain engineering. Following, we outline and present scalable fabrication options for 2D PSCs. Finally, we discussed the perspectives around materials and devices and potential fields for exploration in 2D perovskites, offering insights into future trajectories in the photovoltaic research.

## Conflicts of Interest

The authors declare no conflicts of interest.

## Data Availability

Data sharing not applicable to this article as no datasets were generated or analysed during the current study.

## References

[1] A. Kojima , K. Teshima , Y. Shirai , and T. Miyasaka , “Organometal Halide Perovskites as Visible‐Light Sensitizers for Photovoltaic Cells,” Journal of the American Chemical Society 131 (2009): 6050, 10.1021/ja809598r. 19366264

[2] Y. Huang , K. Yan , X. Wang , et al., “High‐Efficiency Inverted Perovskite Solar Cells via In Situ Passivation Directed Crystallization,” Advanced Materials 36 (2024): 2408101, 10.1002/adma.v36.41. 39140642

[3] H. Li , C. Zhang , C. Gong , et al., “2D/3D Heterojunction Engineering at the Buried Interface towards High‐Performance Inverted Methylammonium‐Free Perovskite Solar Cells,” Nature Energy 8 (2023): 946, 10.1038/s41560-023-01295-8.

[4] J. Jeong , M. Kim , J. Seo , et al., “Pseudo‐Halide Anion Engineering for α‐FAPbI3 Perovskite Solar Cells,” Nature 592 (2021): 381, 10.1038/s41586-021-03406-5. 33820983

[5] K. O. Brinkmann , T. Becker , F. Zimmermann , et al., “Perovskite–organic Tandem Solar Cells with Indium Oxide Interconnect,” Nature 604 (2022): 280, 10.1038/s41586-022-04455-0. 35418631

[6] Y. Lin , Y. Bai , Y. Fang , et al., “Enhanced Thermal Stability in Perovskite Solar Cells by Assembling 2D/3D Stacking Structures,” Journal of Physical Chemistry Letters 9 (2018): 654, 10.1021/acs.jpclett.7b02679. 29350044

[7] R. Wang , J. Xue , L. Meng , et al., “Caffeine Improves the Performance and Thermal Stability of Perovskite Solar Cells,” Joule 3 (2019): 1464, 10.1016/j.joule.2019.04.005.

[8] S. Bian , Y. Wang , F. Zeng , et al., “Multifunctional Interfacial Molecular Bridge Enabled by an Aggregation‐Induced Emission Strategy for Enhancing Efficiency and UV Stability of Perovskite Solar Cells,” Journal of Energy Chemistry 95 (2024): 588, 10.1016/j.jechem.2024.03.059.

[9] K. Wang , Z. Jin , L. Liang , et al., “All‐Inorganic Cesium Lead Iodide Perovskite Solar Cells with Stabilized Efficiency beyond 15%,” Nature Communications 9 (2018): 4544, 10.1038/s41467-018-06915-6. PMC6208436 30382108

[10] R. Prasanna , T. Leijtens , S. P. Dunfield , et al., “Design of Low Bandgap Tin–lead Halide Perovskite Solar Cells to Achieve Thermal, Atmospheric and Operational Stability,” Nature Energy 4 (2019): 939, 10.1038/s41560-019-0471-6.

[11] Q. Zhang , H. Huang , Y. Yang , et al., “A Universal Ternary Solvent System of Surface Passivator Enables Perovskite Solar Cells with Efficiency Exceeding 26%,” Advanced Materials 36 (2024): 2410390, 10.1002/adma.v36.50. 39460404

[12] S. Sidhik , Y. Wang , M. De Siena , et al., “Deterministic Fabrication of 3D/2D Perovskite Bilayer Stacks for Durable and Efficient Solar Cells,” Science 377 (2022): 1425, 10.1126/science.abq7652. 36137050

[13] C. Lin , Y. Tang , Z. Nian , et al., “Intralayer Bidentate Diammoniums for Stable Two‐Dimensional Perovskites,” Nature Chemistry 18 (2026): 275, 10.1038/s41557-025-02038-w. 41491837

[14] W. Zhang , Z. Liu , L. Zhang , et al., “Ultrastable and Efficient Slight‐Interlayer‐Displacement 2D Dion‐Jacobson Perovskite Solar Cells,” Nature Communications 15 (2024): 5709, 10.1038/s41467-024-50018-4. PMC11231157 38977696

[15] G. Wu , R. Liang , M. Ge , G. Sun , Y. Zhang , and G. Xing , “Surface Passivation Using 2D Perovskites toward Efficient and Stable Perovskite Solar Cells,” Advanced Materials 34 (2022): 2105635, 10.1002/adma.v34.8. 34865245

[16] X. Li , S. Aftab , S. Hussain , et al., “Dimensional Diversity (0D, 1D, 2D, and 3D) in Perovskite Solar Cells: Exploring the Potential of Mixed‐Dimensional Integrations,” Journal of Materials Chemistry A 12 (2024): 4421, 10.1039/D3TA06953B.

[17] K. Sakshi , T. Wani , M. Sharma , et al., “Octylammonium Spacer‐Driven Enhanced Stability in Quasi‐Two‐Dimensional Perovskite Solar Cells Fabricated under Ambient Conditions,” ACS Applied Energy Materials 9 (2026): 1058, 10.1021/acsaem.5c03353.

[18] C. Liang , H. Gu , Y. Xia , et al., “Two‐Dimensional Ruddlesden–Popper Layered Perovskite Solar Cells Based on Phase‐Pure Thin Films,” Nature Energy 6 (2020): 38, 10.1038/s41560-020-00721-5.

[19] K. Li , S. Yue , X. Li , et al., “High Efficiency Perovskite Solar Cells Employing Quasi‐2D Ruddlesden‐Popper/Dion‐Jacobson Heterojunctions,” Advanced Functional Materials 32 (2022): 2200024, 10.1002/adfm.v32.21.

[20] X. Li , J. M. Hoffman , and M. G. Kanatzidis , “The 2D Halide Perovskite Rulebook: How the Spacer Influences Everything from the Structure to Optoelectronic Device Efficiency,” Chemical Reviews 121 (2021): 2230, 10.1021/acs.chemrev.0c01006. 33476131

[21] L. Mao , C. C. Stoumpos , and M. G. Kanatzidis , “Two‐Dimensional Hybrid Halide Perovskites: Principles and Promises,” Journal of the American Chemical Society 141 (2019): 1171, 10.1021/jacs.8b10851. 30399319

[22] A. O. El‐Ballouli , O. M. Bakr , and O. F. Mohammed , “Structurally Tunable Two‐Dimensional Layered Perovskites: From Confinement and Enhanced Charge Transport to Prolonged Hot Carrier Cooling Dynamics,” Journal of Physical Chemistry Letters 11 (2020): 5705, 10.1021/acs.jpclett.0c00359. 32574063 PMC7467744

[23] M. Wang , J. You , C. Xu , et al., “Phase Control of Quasi‐2D Perovskite Thin Films by Adding MAPbI_3_ in the Precursor Solution,” Dalton Transactions 51 (2022): 13919, 10.1039/D2DT00966H. 36040451

[24] X. Zhang , L. Einhaus , A. Huijser , and J. E. Ten Elshof , “Phase Distribution Regulation of Formamidinium‐Based Quasi‐2D Perovskites through Solution Engineering,” Journal of Materials Chemistry C 12 (2024): 15671, 10.1039/D4TC02231A. 39246737 PMC11372761

[25] R. Wang , X. Dong , Y. Ma , et al., “Molecular Interfaces Drive Vertical Crystallization in Dion‐Jacobson Perovskite Solar Cells,” Science AdvAnceS 12 (2026): eaea7043, 10.1126/sciadv.aea7043. 42172339 PMC13196774

[26] W. Feng , R. Wang , Y. Ye , et al., “Breaking Electronic Insulation of Monocyclic Aromatic Spacers via Hydrazide‐Induced Orbital Coupling in Ruddlesden‐Popper Perovskites,” Angewandte Chemie 138 (2026): e4293157, 10.1002/ange.v138.26. 42084011

[27] K. Xu , Z. Xing , D. Li , et al., “Boosting Efficiency to 22.73%: Unraveling the Role of Solvent Environment in Low‐Dimensional Perovskites Through Competitive Bonding Interactions,” Advanced Functional Materials 35 (2025): 2415429, 10.1002/adfm.v35.7.

[28] Y. Zhang and N.‐G. Park , “Quasi‐Two‐Dimensional Perovskite Solar Cells with Efficiency Exceeding 22%,” ACS Energy Letters 7 (2022): 757, 10.1021/acsenergylett.1c02645.

[29] A. Z. Chen , M. Shiu , J. H. Ma , et al., “Origin of Vertical Orientation in Two‐Dimensional Metal Halide Perovskites and Its Effect on Photovoltaic Performance,” Nature Communications 9 (2018): 1336, 10.1038/s41467-018-03757-0. PMC5889398 29626205

[30] L. Kuai , J. Li , Y. Li , et al., “Revealing Crystallization Dynamics and the Compositional Control Mechanism of 2D Perovskite Film Growth by In Situ Synchrotron‐Based GIXRD,” ACS Energy Letters 5 (2020): 8, 10.1021/acsenergylett.9b02366.

[31] A. Scardina , T. F. Loeff , N. Mrkyvkova Vikram , et al., “Spacer Cation Design: Promoting Vertical Orientation in Layered Perovskites,” EES Solar 2 (2026): 402, 10.1039/D6EL00006A. 41684509 PMC12893194

[32] D. Lu , G. Lv , Z. Xu , Y. Dong , X. Ji , and Y. Liu , “Thiophene‐Based Two‐Dimensional Dion–Jacobson Perovskite Solar Cells with over 15% Efficiency,” Journal of the American Chemical Society 142 (2020): 11114, 10.1021/jacs.0c03363. 32478512

[33] I. C. Smith , E. T. Hoke , D. Solis‐Ibarra , M. D. McGehee , and H. I. Karunadasa , “A Layered Hybrid Perovskite Solar‐Cell Absorber with Enhanced Moisture Stability,” Angewandte Chemie International Edition 53 (2014): 11232, 10.1002/anie.v53.42. 25196933

[34] C. Xu , L. Cheng , Z. Li , et al., “Fast Solidification and Slow Growth Strategy for High‐Performance Quasi‐2D Perovskite Solar Cells,” Advanced Energy Materials 13 (2023): 2300168, 10.1002/aenm.v13.17.

[35] Y. Yang , C. Liu , O. A. Syzgantseva , et al., “Defect Suppression in Oriented 2D Perovskite Solar Cells with Efficiency over 18% via Rerouting Crystallization Pathway,” Advanced Energy Materials 11 (2021): 2002966, 10.1002/aenm.v11.1.

[36] Z. Liu , G. Liu , C. Xu , and X. Xie , “Scalable Fabrication for Efficient Quasi Two‐Dimensional Perovskite Solar Cells via Ultrasonic Spray‐Coating Method,” Organic Electronics 102 (2022): 106440, 10.1016/j.orgel.2022.106440.

[37] B. Wilk , S. Sahayaraj , M. Ziółek , V. Babu , R. Kudrawiec , and K. Wojciechowski , “Inkjet Printing of Quasi‐2D Perovskite Layers with Optimized Drying Protocol for Efficient Solar Cells,” Advanced Materials Technologies 7 (2022): 2200606, 10.1002/admt.v7.12.

[38] A. Sembito , M. M. Ligavo , J. M. Mwabora , F. W. Nyongesa , and M. Diale , “Characterization of 2D‐PEA2SnI4 Perovskite Thin Films Grown by Sequential Physical Vapor Deposition,” Vacuum 233 (2025): 113954, 10.1016/j.vacuum.2024.113954.

[39] Y. Yun , Q. Chang , J. Yan , et al., “Dimensional Engineering of Interlayer for Efficient Large‐Area Perovskite Solar Cells with High Stability under ISOS‐L‐3 Aging Test,” Science Advances 11 (2025): eadp3112, 10.1126/sciadv.adp3112. 39813355 PMC11734737

[40] C. Zuo , A. D. Scully , D. Vak , et al., “Self‐Assembled 2D Perovskite Layers for Efficient Printable Solar Cells,” Advanced Energy Materials 9 (2019): 1803258, 10.1002/aenm.v9.4.

[41] H. Banerjee , M. K. Nazeeruddin , and S. Chakraborty , “Tuning Electronic and Optical Properties of 2D/3D Interfaces of Hybrid Perovskites through Interfacial Charge Transfer: Prediction of Higher‐Efficiency Interface Solar Cells Using Hybrid‐DFT Methods,” ACS Applied Materials & Interfaces 17 (2025): 19701, 10.1021/acsami.5c00201. 40129233 PMC11969436

[42] A. A. Sutanto , R. Szostak , N. Drigo , et al., “In Situ Analysis Reveals the Role of 2D Perovskite in Preventing Thermal‐Induced Degradation in 2D/3D Perovskite Interfaces,” Nano Letters 20 (2020): 3992, 10.1021/acs.nanolett.0c01271. 32352798 PMC7901643

[43] Z. Fang , M.‐H. Shang , Y. Zheng , et al., “Energy Level Matching and Band Edge Reconfiguration for Enhanced Charge Transport in Dion–Jacobson 3D/2D Perovskite Heterojunctions,” Journal of Physical Chemistry Letters 14 (2023): 6592, 10.1021/acs.jpclett.3c01303. 37459115

[44] G. Grancini , C. Roldán‐Carmona , I. Zimmermann , et al., “1‐Year Stable Perovskite Solar Cells by 2D/3D Interface Engineering,” Nature Communications 8 (2017): 15684, 10.1038/ncomms15684. PMC5461484 28569749

[45] J. Zhang , L. Chu , T. Liu , et al., “Engineering Spacer Conjugation for Efficient and Stable 2D/3D Perovskite Solar Cells and Modules,” Angewandte Chemie International Edition 64 (2025): e202413303, 10.1002/anie.v64.1. 39370407

[46] N.‐K. Pendyala , A. Kolay , Y. N. Ponnada , A. Guerrero , and L. Etgar , “Evolving Solar Cell Manufacturing: the Promising Outlook of Open‐Air Perovskite Printing,” Sustainable Energy Fuels 9 (2025): 1633, 10.1039/D5SE00002E.

[47] S. Lee , H. Cho , S. Kang , O. J. Oh , D. H. Kim , and J. H. Noh , “Deciphering 2D Perovskite's Role in Perovskite Solar Cells *via* Intact 3D/2D Junctions,” Energy & Environmental Science 17 (2024): 6234, 10.1039/D3EE04520J.

[48] D. Yu , F. Cao , C. Su , and G. Xing , “Exploring, Identifying, and Removing the Efficiency‐Limiting Factor of Mixed‐Dimensional 2D/3D Perovskite Solar Cells,” Accounts of Chemical Research 56 (2023): 959, 10.1021/acs.accounts.3c00015. 37013981

[49] X. Li , J. Hoffman , W. Ke , et al., “Two‐Dimensional Halide Perovskites Incorporating Straight Chain Symmetric Diammonium Ions, (NH_3_C_ *m* _H_2*m* _NH_3_)(CH_3_NH_3_)_ *n*−1_Pb_ *n* _I_3*n*+1_ (*m* = 4–9; *n* = 1–4),” Journal of the American Chemical Society 140 (2018): 12226, 10.1021/jacs.8b07712. 30169031

[50] L. Du , J. Liao , K. Li , et al., “Preparation of High‐Performance Quasi‐Two‐Dimensional (Q‐2D) Perovskite Solar Cells by Fluorinated Benzylamine Groups at Different Substitution Positions,” Physical Chemistry Chemical Physics 27 (2025): 4669, 10.1039/D4CP04357J. 39935363

[51] S. Ramos‐Terrón , A. D. Jodlowski , C. Verdugo‐Escamilla , L. Camacho , and G. De Miguel , “Relaxing the Goldschmidt Tolerance Factor: Sizable Incorporation of the Guanidinium Cation into a Two‐Dimensional Ruddlesden–Popper Perovskite,” Chemistry of Materials 32 (2020): 4024, 10.1021/acs.chemmater.0c00613.

[52] Y. Chen , Y. Sun , J. Peng , J. Tang , K. Zheng , and Z. Liang , “2D Ruddlesden–Popper Perovskites for Optoelectronics,” Advanced Materials 30 (2018): 1703487, 10.1002/adma.v30.2. 29028138

[53] D. Thrithamarassery Gangadharan and D. Ma , “Searching for Stability at Lower Dimensions: Current Trends and Future Prospects of Layered Perovskite Solar Cells,” Energy & Environmental Science 12 (2019): 2860, 10.1039/C9EE01591D.

[54] P. Huang , S. Kazim , M. Wang , and S. Ahmad , “Toward Phase Stability: Dion–Jacobson Layered Perovskite for Solar Cells,” ACS Energy Letters 4 (2019): 2960, 10.1021/acsenergylett.9b02063.

[55] E. S. Vasileiadou , B. Wang , I. Spanopoulos , I. Hadar , A. Navrotsky , and M. G. Kanatzidis , “Insight on the Stability of Thick Layers in 2D Ruddlesden–Popper and Dion–Jacobson Lead Iodide Perovskites,” Journal of the American Chemical Society 143 (2021): 2523, 10.1021/jacs.0c11328. 33534580

[56] J. Cao , C. Duan , Z. Li , et al., “Theoretical Selection of 2D Perovskite for Constructing Efficient Heterojunction Solar Cells,” ACS Materials Letters 5 (2023): 970, 10.1021/acsmaterialslett.3c00027.

[57] S. Zhao , C. Lan , H. Li , C. Zhang , and T. Ma , “Aurivillius Halide Perovskite: A New Family of Two‐Dimensional Materials for Optoelectronic Applications,” Journal of Physical Chemistry C 124 (2020): 1788, 10.1021/acs.jpcc.9b08450.

[58] W. Li , S. Sidhik , B. Traore , et al., “Light‐Activated Interlayer Contraction in Two‐Dimensional Perovskites for High‐Efficiency Solar Cells,” Nature Nanotechnology 17 (2022): 45, 10.1038/s41565-021-01010-2. 34811551

[59] M. Baranowski and P. Plochocka , “Excitons in Metal‐Halide Perovskites,” Advanced Energy Materials 10 (2020): 1903659, 10.1002/aenm.v10.26.

[60] V. D’Innocenzo , G. Grancini , M. J. P. Alcocer , et al., “Excitons versus Free Charges in Organo‐Lead Tri‐Halide Perovskites,” Nature Communications 5 (2014): 3586, 10.1038/ncomms4586. 24710005

[61] J. Kang and L.‐W. Wang , “High Defect Tolerance in Lead Halide Perovskite CsPbBr_3_ ,” Journal of Physical Chemistry Letters 8 (2017): 489, 10.1021/acs.jpclett.6b02800. 28071911

[62] G. Xing , N. Mathews , S. Sun , et al., “Long‐Range Balanced Electron‐ and Hole‐Transport Lengths in Organic‐Inorganic CH_3_NH_3_PbI_3_ ,” Science 342 (2013): 344, 10.1126/science.1243167. 24136965

[63] J.‐C. Blancon , A. V. Stier , H. Tsai , et al., “Scaling Law for Excitons in 2D Perovskite Quantum Wells,” Nature Communications 9 (2018): 2254, 10.1038/s41467-018-04659-x. PMC5993799 29884900

[64] K. R. Hansen , C. Y. Wong , C. E. McClure , et al., “Mechanistic Origins of Excitonic Properties in 2D Perovskites: Implications for Exciton Engineering,” Matter 6 (2023): 3463, 10.1016/j.matt.2023.07.004.

[65] M. Seitz , A. J. Magdaleno , N. Alcázar‐Cano , et al., “Exciton Diffusion in Two‐Dimensional Metal‐Halide Perovskites,” Nature Communications 11 (2020): 2035, 10.1038/s41467-020-15882-w. PMC7184754 32341361

[66] A. J. Magdaleno , M. Seitz , M. Frising , A. Herranz De La Cruz , A. I. Fernández‐Domínguez , and F. Prins , “Efficient Interlayer Exciton Transport in Two‐Dimensional Metal‐Halide Perovskites,” Materials Horizons 8 (2021): 639, 10.1039/D0MH01723J. 34821281

[67] V. Adinolfi , W. Peng , G. Walters , O. M. Bakr , and E. H. Sargent , “The Electrical and Optical Properties of Organometal Halide Perovskites Relevant to Optoelectronic Performance,” Advanced Materials 30 (2018): 1700764, 10.1002/adma.v30.1. 29024039

[68] T. J. Sheehan , S. Saris , and W. A. Tisdale , “Exciton Transport in Perovskite Materials,” Advanced Materials 37 (2025): 2415757, 10.1002/adma.v37.25. 39737764

[69] E. Penzo , A. Loiudice , E. S. Barnard , et al., “Long‐Range Exciton Diffusion in Two‐Dimensional Assemblies of Cesium Lead Bromide Perovskite Nanocrystals,” ACS Nano 14 (2020): 6999, 10.1021/acsnano.0c01536. 32459460

[70] Y. Song , Y. Zhou , C. Chen , et al., “Structure–Emission Property Relationship of Bilayer 2D Hybrid Perovskites,” Journal of the American Chemical Society 147 (2025): 19902, 10.1021/jacs.5c04417. 40304134 PMC12164342

[71] D. Ghosh , D. Acharya , L. Pedesseau , et al., “Charge Carrier Dynamics in Two‐Dimensional Hybrid Perovskites: Dion–Jacobson *vs*. Ruddlesden–Popper Phases,” Journal of Materials Chemistry A 8 (2020): 22009, 10.1039/D0TA07205B.

[72] M. Li , Y. Xu , S. Han , et al., “Giant and Broadband Multiphoton Absorption Nonlinearities of a 2D Organometallic Perovskite Ferroelectric,” Advanced Materials 32 (2020): 2002972, 10.1002/adma.v32.36. 32705717

[73] H. Tsai , W. Nie , J.‐C. Blancon , et al., “High‐Efficiency Two‐Dimensional Ruddlesden–Popper Perovskite Solar Cells,” Nature 536 (2016): 312, 10.1038/nature18306. 27383783

[74] L. Gao , F. Zhang , C. Xiao , et al., “Improving Charge Transport via Intermediate‐Controlled Crystal Growth in 2D Perovskite Solar Cells,” Advanced Functional Materials 29 (2019): 1901652, 10.1002/adfm.v29.47.

[75] T. Zhou , Q. Li , and L. Zhou , “Fluorinated Quasi 2D Perovskite Solar Cells with Improved Stability and Over 19% Efficiency,” Advanced Energy Materials 14 (2024): 2400050, 10.1002/aenm.v14.22.

[76] Z. Xu , D. Lu , X. Dong , M. Chen , Q. Fu , and Y. Liu , “Highly Efficient and Stable Dion−Jacobson Perovskite Solar Cells Enabled by Extended π‐Conjugation of Organic Spacer,” Advanced Materials 33 (2021): 2105083, 10.1002/adma.v33.51. 34655111

[77] Q. Shang , Y. Wang , Y. Zhong , et al., “Unveiling Structurally Engineered Carrier Dynamics in Hybrid Quasi‐Two‐Dimensional Perovskite Thin Films toward Controllable Emission,” Journal of Physical Chemistry Letters 8 (2017): 4431, 10.1021/acs.jpclett.7b01857. 28845670

[78] R. L. Milot , R. J. Sutton , G. E. Eperon , et al., “Charge‐Carrier Dynamics in 2D Hybrid Metal–Halide Perovskites,” Nano Letters 16 (2016): 7001, 10.1021/acs.nanolett.6b03114. 27689536

[79] Y. Zheng , T. Niu , J. Qiu , et al., “Oriented and Uniform Distribution of Dion–Jacobson Phase Perovskites Controlled by Quantum Well Barrier Thickness,” Solar RRL 3 (2019): 1900090, 10.1002/solr.v3.9.

[80] X. Zhao , T. Liu , and Y. Loo , “Advancing 2D Perovskites for Efficient and Stable Solar Cells: Challenges and Opportunities,” Advanced Materials 34 (2022): 2105849, 10.1002/adma.v34.3. 34668250

[81] T. He , S. Li , Y. Jiang , et al., “Reduced‐Dimensional Perovskite Photovoltaics with Homogeneous Energy Landscape,” Nature Communications 11 (2020): 1672, 10.1038/s41467-020-15451-1. PMC7125147 32246083

[82] J. Qiu , Y. Zheng , Y. Xia , L. Chao , Y. Chen , and W. Huang , “Rapid Crystallization for Efficient 2D Ruddlesden–Popper (2DRP) Perovskite Solar Cells,” Advanced Functional Materials 29 (2019): 1806831, 10.1002/adfm.v29.47.

[83] X. Zhang , R. Munir , Z. Xu , et al., “Phase Transition Control for High Performance Ruddlesden–Popper Perovskite Solar Cells,” Advanced Materials 30 (2018): 1707166, 10.1002/adma.v30.21. 29611240

[84] Y. Chen , J. Hu , Z. Xu , et al., “Managing Phase Orientation and Crystallinity of Printed Dion–Jacobson 2D Perovskite Layers via Controlling Crystallization Kinetics,” Advanced Functional Materials 32 (2022): 2112146, 10.1002/adfm.v32.19.

[85] C. M. M. Soe , G. P. Nagabhushana , R. Shivaramaiah , et al., “Structural and Thermodynamic Limits of Layer Thickness in 2D Halide Perovskites,” Proceedings of the National Academy of Sciences 116 (2019): 58, 10.1073/pnas.1811006115. PMC6320524 30563858

[86] D. B. Straus and C. R. Kagan , “Electrons, Excitons, and Phonons in Two‐Dimensional Hybrid Perovskites: Connecting Structural, Optical, and Electronic Properties,” Journal of Physical Chemistry Letters 9 (2018): 1434, 10.1021/acs.jpclett.8b00201. 29481089

[87] N. Liu , P. Liu , H. Ren , et al., “Probing Phase Distribution in 2D Perovskites for Efficient Device Design,” ACS Applied Materials & Interfaces 12 (2020): 3127, 10.1021/acsami.9b17047. 31833753

[88] C. Qin , L. Xu , Z. Zhou , et al., “Carrier Dynamics in Two‐Dimensional Perovskites: Dion–Jacobson *vs*. Ruddlesden–Popper Thin Films,” Journal of Materials Chemistry A 10 (2022): 3069, 10.1039/D1TA09549H.

[89] G. Wu , R. Liang , Z. Zhang , M. Ge , G. Xing , and G. Sun , “2D Hybrid Halide Perovskites: Structure, Properties, and Applications in Solar Cells,” Small 17 (2021): 2103514, 10.1002/smll.v17.43. 34590421

[90] R. M. Ansari , A. D. Salunke , M. Rahil , and S. Ahmad , “Strong Photocurrent from Solution‐Processed Ruddlesden–Popper 2D Perovskite–MoS_2_ Hybrid Heterojunctions,” Advanced Materials Interfaces 10 (2023): 2202170, 10.1002/admi.v10.10.

[91] J.‐C. Blancon , H. Tsai , W. Nie , et al., “Extremely Efficient Internal Exciton Dissociation through Edge States in Layered 2D Perovskites,” Science 355 (2017): 1288, 10.1126/science.aal4211. 28280250

[92] S. Yang , W. Niu , A. Wang , et al., “Ultrathin Two‐Dimensional Organic–Inorganic Hybrid Perovskite Nanosheets with Bright, Tunable Photoluminescence and High Stability,” Angewandte Chemie International Edition 56 (2017): 4252, 10.1002/anie.v56.15. 28295961

[93] C. Ma , F. T. Eickemeyer , S.‐H. Lee , et al., “Unveiling Facet‐Dependent Degradation and Facet Engineering for Stable Perovskite Solar Cells,” Science 379 (2023): 173, 10.1126/science.adf3349. 36634188

[94] J. Tang , W. Tian , C. Zhao , et al., “Imaging the Moisture‐Induced Degradation Process of 2D Organolead Halide Perovskites,” ACS Omega 7 (2022): 10365, 10.1021/acsomega.1c06989. 35382338 PMC8973051

[95] E. Mahal , S. S. Manna , S. Das , and B. Pathak , “Understanding Moisture Stability and Degradation Mechanisms of 2D Hybrid Perovskites: Insights from *Ab Initio* Molecular Dynamics Simulations,” Energy Advances 3 (2024): 1992, 10.1039/D4YA00235K.

[96] J. Hidalgo , W. Kaiser , Y. An , et al., “Synergistic Role of Water and Oxygen Leads to Degradation in Formamidinium‐Based Halide Perovskites,” Journal of the American Chemical Society 145 (2023): 24549–24557, 10.1021/jacs.3c05657. 37917967 PMC10655111

[97] Y. W. Woo , Y.‐K. Jung , G. Y. Kim , S. Kim , and A. Walsh , “Factors Influencing Halide Vacancy Transport in Perovskite Solar Cells,” Discover Materials 2 (2022): 8, 10.1007/s43939-022-00029-z.

[98] Y. Wang , D. Li , Z. Xing , et al., “Quantum Well Growth Management to Smooth the Energy Transfer Pathway for Quasi‐2D Perovskite Solar Cells,” Advanced Functional Materials 34 (2024): 2401203, 10.1002/adfm.v34.36.

[99] W. Li , X. Gu , C. Shan , X. Lai , X. W. Sun , and A. K. K. Kyaw , “Efficient and Stable Mesoscopic Perovskite Solar Cell in High Humidity by Localized Dion‐Jacobson 2D‐3D Heterostructures,” Nano Energy 91 (2022): 106666, 10.1016/j.nanoen.2021.106666.

[100] Z. Peng , A. Vincze , F. Streller , et al., “Revealing Degradation Mechanisms in 3D/2D Perovskite Solar Cells under Photothermal Accelerated Ageing,” Energy & Environmental Science 17 (2024): 8313, 10.1039/D4EE03869J.

[101] M. Y. Choi , S. J. Lee , H. Ju , and A. R. Lim , “Phase Transition, Thermal Stability, and Molecular Dynamics of Organic–inorganic Hybrid Perovskite [NH_3_(CH_2_)_6_NH_3_]CuCl_4_ Crystals,” RSC Advances 12 (2022): 20679, 10.1039/D2RA02975H. 35919167 PMC9297128

[102] A. Maiti , K. Agarwal , S. Gonzalez‐Munoz , and O. V. Kolosov , “Quantifying Anisotropic Thermal Transport in Two‐Dimensional Perovskite (PEA 2 PbI 4) through Cross‐Sectional Scanning Thermal Microscopy,” Physical Review Materials 7 (2023): 023801, 10.1103/PhysRevMaterials.7.023801.

[103] W. Fu , H. Liu , X. Shi , L. Zuo , X. Li , and A. K.‐Y. Jen , “Tailoring the Functionality of Organic Spacer Cations for Efficient and Stable Quasi‐2D Perovskite Solar Cells,” Advanced Functional Materials 29 (2019): 1900221, 10.1002/adfm.v29.25.

[104] H. Ren , S. Yu , L. Chao , et al., “Efficient and Stable Ruddlesden–Popper Perovskite Solar Cell with Tailored Interlayer Molecular Interaction,” Nature Photonics 14 (2020): 154, 10.1038/s41566-019-0572-6.

[105] G. Wu , T. Liu , M. Hu , et al., “Crystallinity and Phase Control in Formamidinium‐Based Dion–Jacobson 2D Perovskite via Seed‐Induced Growth for Efficient Photovoltaics,” Advanced Materials 35 (2023): 2303061, 10.1002/adma.v35.36. 37235878

[106] G. Grimaldi , I. Schuringa , J. J. Geuchies , et al., “Atmospheric Exposure Triggers Light‐Induced Degradation in 2D Lead‐Halide Perovskites,” ACS Energy Letters 9 (2024): 5771, 10.1021/acsenergylett.4c02300. 39698336 PMC11650760

[107] D. J. Slotcavage , H. I. Karunadasa , and M. D. McGehee , “Light‐Induced Phase Segregation in Halide‐Perovskite Absorbers,” ACS Energy Letters 1 (2016): 1199, 10.1021/acsenergylett.6b00495.

[108] J. Kim , C. Oh , I. Hwang , et al., “Efficient and Stable Quasi‐2D Ruddlesden–Popper Perovskite Solar Cells by Tailoring Crystal Orientation and Passivating Surface Defects,” Advanced Materials 35 (2023): 2302143, 10.1002/adma.v35.31. 37099626

[109] Z. Huang , A. H. Proppe , H. Tan , et al., “Suppressed Ion Migration in Reduced‐Dimensional Perovskites Improves Operating Stability,” ACS Energy Letters 4 (2019): 1521, 10.1021/acsenergylett.9b00892.

[110] P. Mathew , J. Cho , and P. V. Kamat , “Ramifications of Ion Migration in 2D Lead Halide Perovskites,” ACS Energy Letters 9 (2024): 1103, 10.1021/acsenergylett.4c00093.

[111] K. T. Tanko , S. R. Raga , N. Vahedigharehchopogh , et al., “The Roles of Ion Migration on Perovskite Solar Cell Operational Stability at Various Illumination Intensities,” Solar RRL 9 (2025): 2500162, 10.1002/solr.v9.12.

[112] Y. Zhao , W. Zhou , Z. Han , D. Yu , and Q. Zhao , “Effects of Ion Migration and Improvement Strategies for the Operational Stability of Perovskite Solar Cells,” Physical Chemistry Chemical Physics 23 (2021): 94, 10.1039/D0CP04418K. 33325463

[113] Y. Wang , J. Chen , Y. Zhang , et al., “Ordered Perovskite Structure with Functional Units for High Performance and Stable Solar Cells,” Advanced Materials 36 (2024): 2401416, 10.1002/adma.v36.25. 38571375

[114] L. Zhang , X. Ren , F. Gao , et al., “The Intrinsic Instability of Lead Halide Perovskite Solar Cells,” Materials Today 94 (2026): 103251, 10.1016/j.mattod.2026.103251.

[115] H. Zhu , S. Teale , M. N. Lintangpradipto , et al., “Long‐Term Operating Stability in Perovskite Photovoltaics,” Nature Reviews. Materials 8 (2023): 569, 10.1038/s41578-023-00582-w.

[116] M. Shao , T. Bie , L. Yang , et al., “Over 21% Efficiency Stable 2D Perovskite Solar Cells,” Advanced Materials 34 (2022): 2107211, 10.1002/adma.v34.1. 34648207

[117] Z. Xing , B. Fan , X. Meng , et al., “Repairing Humidity‐Induced Interfacial Degradation in Quasi‐2D Perovskite Solar Cells Printed in Ambient Air,” Energy & Environmental Science 17 (2024): 3660, 10.1039/D4EE00912F.

[118] H. Zhang , R. Wang , L. Yang , Z. Hu , H. Liu , and Y. Liu , “Modulating the Dipole Moment of Secondary Ammonium Spacers for Efficient 2D Ruddlesden‐Popper Perovskite Solar Cells,” Angewandte Chemie International Edition 63 (2024): e202318206, 10.1002/anie.v63.7. 38165142

[119] Y. Huang , Y. Li , E. L. Lim , et al., “Stable Layered 2D Perovskite Solar Cells with an Efficiency of over 19% via Multifunctional Interfacial Engineering,” Journal of the American Chemical Society 143 (2021): 3911, 10.1021/jacs.0c13087. 33660986

[120] Y. Zhang , M. Chen , T. He , et al., “Highly Efficient and Stable FA‐Based Quasi‐2D Ruddlesden–Popper Perovskite Solar Cells by the Incorporation of β‐Fluorophenylethanamine Cations,” Advanced Materials 35 (2023): 2210836, 10.1002/adma.v35.17. 36744546

[121] J. Xiang , X. Li , S. Gong , S. Wang , X. Chen , and F. Zhang , “Green‐Antisolvent‐Induced Homogeneous Phase Distribution for Efficient and Stable MA‐Free 2D Perovskite Solar Cells,” Chemical Engineering Journal 460 (2023): 141758, 10.1016/j.cej.2023.141758.

[122] W. Zhang , X. Wu , J. Zhou , et al., “Pseudohalide‐Assisted Growth of Oriented Large Grains for High‐Performance and Stable 2D Perovskite Solar Cells,” ACS Energy Letters 7 (2022): 1842, 10.1021/acsenergylett.2c00485.

[123] G. Wu , T. Yang , X. Li , et al., “Molecular Engineering for Two‐Dimensional Perovskites with Photovoltaic Efficiency Exceeding 18%,” Matter 4 (2021): 582, 10.1016/j.matt.2020.11.011.

[124] R. Yang , R. Li , Y. Cao , et al., “Oriented Quasi‐2D Perovskites for High Performance Optoelectronic Devices,” Advanced Materials 30 (2018): 1804771, 10.1002/adma.v30.51. 30345566

[125] J. Zhang , J. Qin , M. Wang , et al., “Uniform Permutation of Quasi‐2D Perovskites by Vacuum Poling for Efficient, High‐Fill‐Factor Solar Cells,” Joule 3 (2019): 3061, 10.1016/j.joule.2019.09.020.

[126] Q. Li , Y. Dong , G. Lv , et al., “Fluorinated Aromatic Formamidinium Spacers Boost Efficiency of Layered Ruddlesden–Popper Perovskite Solar Cells,” ACS Energy Letters 6 (2021): 2072, 10.1021/acsenergylett.1c00620.

[127] J. Shi , Y. Gao , X. Gao , et al., “Fluorinated Low‐Dimensional Ruddlesden–Popper Perovskite Solar Cells with over 17% Power Conversion Efficiency and Improved Stability,” Advanced Materials 31 (2019): 1901673, 10.1002/adma.v31.37. 31379023

[128] G. Wu , X. Li , J. Zhou , et al., “Fine Multi‐Phase Alignments in 2D Perovskite Solar Cells with Efficiency over 17% via Slow Post‐Annealing,” Advanced Materials 31 (2019): 1903889, 10.1002/adma.v31.42. 31475406

[129] Z. Xu , D. Lu , F. Liu , et al., “Phase Distribution and Carrier Dynamics in Multiple‐Ring Aromatic Spacer‐Based Two‐Dimensional Ruddlesden–Popper Perovskite Solar Cells,” ACS Nano 14 (2020): 4871, 10.1021/acsnano.0c00875. 32243131

[130] L. Chao , T. Niu , Y. Xia , X. Ran , Y. Chen , and W. Huang , “Efficient and Stable Low‐Dimensional Ruddlesden–Popper Perovskite Solar Cells Enabled by Reducing Tunnel Barrier,” Journal of Physical Chemistry Letters 10 (2019): 1173, 10.1021/acs.jpclett.9b00276. 30807176

[131] C. Ma , D. Shen , T. Ng , M. Lo , and C. Lee , “2D Perovskites with Short Interlayer Distance for High‐Performance Solar Cell Application,” Advanced Materials 30 (2018): 1800710, 10.1002/adma.v30.22. 29665101

[132] Y. Wang , I. Ahmad , T. Leung , et al., “Encapsulation and Stability Testing of Perovskite Solar Cells for Real Life Applications,” ACS Materials Au 2 (2022): 215, 10.1021/acsmaterialsau.1c00045. 36855381 PMC9888620

[133] M. Seitz , P. Gant , A. Castellanos‐Gomez , and F. Prins , “Long‐Term Stabilization of Two‐Dimensional Perovskites by Encapsulation with Hexagonal Boron Nitride,” Nanomaterials 9 (2019): 1120, 10.3390/nano9081120. 31382621 PMC6724044

[134] G. Li , J. Wu , J. Song , et al., “Grain Boundary Encapsulation for Efficient Perovskite Solar Cells via PbI_2_ ‐Derived Low‐Dimensional Structure,” Advanced Functional Materials 36 (2026): e31852, 10.1002/adfm.v36.35.

[135] H. Zhang , Y. Bi , Y. Wang , et al., “Dual‐Interface Passivation to Improve the Efficiency and Stability of Inverted Flexible Perovskite Solar Cells by In‐Situ Constructing 2D/3D/2D Perovskite Double Heterojunctions,” Journal of Power Sources 629 (2025): 235973, 10.1016/j.jpowsour.2024.235973.

[136] S. Mondal , N. Eguchi , N. Nishimura , et al., “Mixed 2D‐Cation Passivation towards Improved Durability of Perovskite Solar Cells and Dynamics of 2D‐Perovskites under Light Irradiation and at High Temperature,” Sustainable Energy Fuels 9 (2024): 247, 10.1039/D4SE01227E.

[137] B. Cheng , T.‐Y. Li , P. Maity , et al., “Extremely Reduced Dielectric Confinement in Two‐Dimensional Hybrid Perovskites with Large Polar Organics,” Communications Physics 1 (2018): 80, 10.1038/s42005-018-0082-8.

[138] Q. Fu , M. Chen , Q. Li , H. Liu , R. Wang , and Y. Liu , “Selenophene‐Based 2D Ruddlesden‐Popper Perovskite Solar Cells with an Efficiency Exceeding 19%,” Journal of the American Chemical Society 145 (2023): 21687, 10.1021/jacs.3c08604. 37750835

[139] H.‐J. Bae , W. Lee , S. Yun , et al., “Tailoring RP‐Structured Two‐Dimensional Perovskite with Controlled Interlayer Distance,” APL Energy 3 (2025): 026107, 10.1063/5.0273430.

[140] H. Tsai , R. Asadpour , J.‐C. Blancon , et al., “Design Principles for Electronic Charge Transport in Solution‐Processed Vertically Stacked 2D Perovskite Quantum Wells,” Nature Communications 9 (2018): 2130, 10.1038/s41467-018-04430-2. PMC5976721 29849026

[141] W. Tress , “Perovskite Solar Cells on the Way to Their Radiative Efficiency Limit – Insights Into a Success Story of High Open‐Circuit Voltage and Low Recombination,” Advanced Energy Materials 7 (2017): 1602358, 10.1002/aenm.v7.14.

[142] U. Rau and T. Kirchartz , “Charge Carrier Collection and Contact Selectivity in Solar Cells,” Advanced Materials Interfaces 6 (2019): 1900252, 10.1002/admi.v6.20.

[143] M. Stolterfoht , C. M. Wolff , Y. Amir , et al., “Approaching the Fill Factor Shockley–Queisser Limit in Stable, Dopant‐Free Triple Cation Perovskite Solar Cells,” Energy & Environmental Science 10 (2017): 1530, 10.1039/C7EE00899F.

[144] Z. Wei , Q. Zhou , X. Niu , et al., “Surpassing 90% Shockley–Queisser *V* _C_ Limit in 1.79 eV Wide‐Bandgap Perovskite Solar Cells Using Bromine‐Substituted Self‐Assembled Monolayers,” Energy & Environmental Science 18 (2025): 1847, 10.1039/D4EE04029E.

[145] N. Mundhaas , Z. J. Yu , K. A. Bush , et al., “Series Resistance Measurements of Perovskite Solar Cells Using *J_Sc_ * – *V_Oc_ * Measurements,” Solar RRL 3 (2019): 1800378, 10.1002/solr.v3.4.

[146] S. Zhang , F. Ye , X. Wang , et al., “Minimizing Buried Interfacial Defects for Efficient Inverted Perovskite Solar Cells,” Science 380 (2023): 404, 10.1126/science.adg3755. 37104579

[147] J. Wu , J. Lee , Y.‐C. Chin , et al., “Exceptionally Low Charge Trapping Enables Highly Efficient Organic Bulk Heterojunction Solar Cells,” Energy & Environmental Science 13 (2020): 2422, 10.1039/D0EE01338B.

[148] J. Gu and Y. Fu , “Is There an Optimal Spacer Cation for Two‐Dimensional Lead Iodide Perovskites?,” ACS Materials Au 5 (2025): 24, 10.1021/acsmaterialsau.4c00101. 39802148 PMC11718535

[149] X. Liu , H. Yan , Z. Shu , X. Cui , and Y. Cai , “Theoretical Insights into Spacer Molecule Design to Tune Stability, Dielectric, and Exciton Properties in 2D Perovskites,” Nanoscale 17 (2025): 2658, 10.1039/D4NR04406A. 39820272

[150] Y. Boeije , F. Lie , M. Dubajić , et al., “Molecular Engineering of Interlayer Exciton Delocalization in 2D Perovskites,” Journal of the American Chemical Society 147 (2025): 31541, 10.1021/jacs.5c05621. 40841881 PMC12412180

[151] Z. Li , H. Wu , Z. Zhang , et al., “Cation‐π/π–π Synergy Induced Self‐Assembly of Semiconductor Spacers for High Efficiency and Stable 2D/3D Perovskite Solar Cells,” Advanced Materials 37 (2025): e11235, 10.1002/adma.v37.44. 40838450

[152] X. Li , B. Li , J. Chang , et al., “(C_6_H_5_CH_2_NH_3_)_2_CuBr_4_: A Lead‐Free, Highly Stable Two‐Dimensional Perovskite for Solar Cell Applications,” ACS Applied Energy Materials 1 (2018): 2709, 10.1021/acsaem.8b00372.

[153] N. Bisht , I. Sharma , P. Patil , et al., “Comparative Performance Simulation Study of Germanium‐Based Perovskite Solar Cells Using Scaps‐1d,” 345 (2025): 131241, 10.1016/j.matchemphys.2025.131241.

[154] P. Cheng , T. Wu , J. Zhang , et al., “(C_6_H_5_C_2_H_4_NH_3_)_2_GeI_4_: A Layered Two‐Dimensional Perovskite with Potential for Photovoltaic Applications,” Journal of Physical Chemistry Letters 8 (2017): 4402, 10.1021/acs.jpclett.7b01985. 28856895

[155] X. Jiang , H. Li , Q. Zhou , et al., “One‐Step Synthesis of SnI_2_ ·(DMSO)_ *x* _ Adducts for High‐Performance Tin Perovskite Solar Cells,” Journal of the American Chemical Society 143 (2021): 10970, 10.1021/jacs.1c03032. 34196528

[156] Y. Wei , Y. Sun , Y. Liu , et al., “Impact of Bromide Incorporation on Strain Modulation in 2D Ruddlesden‐Popper Perovskite Solar Cells,” Cell Reports Physical Science 4 (2023): 101739, 10.1016/j.xcrp.2023.101739.

[157] W. Ma , Z. Zhang , Y. Liu , H. Gao , and Y. Mao , “Enhanced Efficiency and Stability of Quasi Two‐Dimensional Perovskite Solar Cells via Dual Additives,” Journal of Alloys and Compounds 972 (2024): 172841, 10.1016/j.jallcom.2023.172841.

[158] M. Liu and T. Pauporté , “Additive Engineering for Stable and Efficient Dion–Jacobson Phase Perovskite Solar Cells,” Nano‐Micro Lett 15 (2023): 134, 10.1007/s40820-023-01110-9. PMC10205963 37221320

[159] M. D. Malouangou , J. Tsiba Matondo , L. Bai , M. Mbumba , Y. F. Yang , M. W. Akram , and M. Guli , “A Synergy Effect of Coadditives for Vertical Orientation of Two‐Dimensional Perovskite Solar Cells Based on Butylammonium Iodide with Improved Efficiency,” ACS Applied Energy Materials 4 (2021): 13216, 10.1021/acsaem.1c02812.

[160] X. Lian , J. Chen , M. Qin , et al., “The Second Spacer Cation Assisted Growth of a 2D Perovskite Film with Oriented Large Grain for Highly Efficient and Stable Solar Cells,” Angewandte Chemie 131 (2019): 9509, 10.1002/ange.v131.28. 31066152

[161] X. Li , G. Wu , M. Wang , et al., “Water‐Assisted Crystal Growth in Quasi‐2D Perovskites with Enhanced Charge Transport and Photovoltaic Performance,” Advanced Energy Materials 10 (2020): 2001832, 10.1002/aenm.v10.37.

[162] H. Zheng , T. Zhang , Y. Wang , et al., “Zwitterion‐Assisted Crystal Growth of 2D Perovskites with Unfavorable Phase Suppression for High‐Performance Solar Cells,” ACS Applied Materials & Interfaces 14 (2022): 814, 10.1021/acsami.1c19263. 34963289

[163] D. H. Cao , C. C. Stoumpos , O. K. Farha , J. T. Hupp , and M. G. Kanatzidis , “2D Homologous Perovskites as Light‐Absorbing Materials for Solar Cell Applications,” Journal of the American Chemical Society 137 (2015): 7843, 10.1021/jacs.5b03796. 26020457

[164] F. Zhang , H. Lu , J. Tong , J. J. Berry , M. C. Beard , and K. Zhu , “Advances in Two‐Dimensional Organic–inorganic Hybrid Perovskites,” Energy & Environmental Science 13 (2020): 1154, 10.1039/C9EE03757H.

[165] J. Mao , Y. Wei , Y. Ding , et al., “Quantum Well Engineering in Quasi‐2D Perovskites Unlocks Efficiency‐Stability Synergy for Photovoltaics,” Journal of Energy Chemistry 111 (2025): 1042, 10.1016/j.jechem.2025.07.085.

[166] B. Chang , Y. Wu , X. Tong , et al., “Edge/Corner‐Sharing 2D Perovskite Functional Layer for Efficient and Stable Inverted Perovskite Solar Cells,” Angewandte Chemie International Edition 64 (2025): e202509328, 10.1002/anie.v64.39. 40810636

[167] R. Wang , J. Zhang , Y. Cao , et al., “Reconstruction of the Surface for Efficient 3D/Quasi‐2D Heterostructured Inverted Perovskite Solar Cells,” Advanced Functional Materials 36 (2026): e17633, 10.1002/adfm.v36.15.

[168] C. Liu , Y. Yang , H. Chen , et al., “Two‐Dimensional Perovskitoids Enhance Stability in Perovskite Solar Cells,” Nature 633 (2024): 359, 10.1038/s41586-024-07764-8. 38977018

[169] Z. Song , Y. Zou , Y. Gao , et al., “Buried and Bulk Synergistic Engineering Enables High‐Performance Inverted 2D/3D Perovskite Solar Cells,” Energy & Environmental Science 18 (2025): 3740, 10.1039/D5EE00156K.

[170] T. Duan , S. You , M. Chen , et al., “Chiral‐Structured Heterointerfaces Enable Durable Perovskite Solar Cells,” Science 384 (2024): 878, 10.1126/science.ado5172. 38781395

[171] H. Wang , S. Su , Y. Chen , et al., “Impurity‐Healing Interface Engineering for Efficient Perovskite Submodules,” Nature 634 (2024): 1091, 10.1038/s41586-024-08073-w. 39326517

[172] X. Chang , R. Azmi , T. Yang , et al., “Solvent‐Dripping Modulated 3D/2D Heterostructures for High‐Performance Perovskite Solar Cells,” Nature Communications 16 (2025): 1042, 10.1038/s41467-025-56409-5. PMC11763036 39863604

[173] J. Lim , S. Lee , H. Shim , et al., “Efficient Charge Separation at Localized 2D Ferroelectric Domains in Perovskite Solar Cells,” Energy & Environmental Science 18 (2025): 5287, 10.1039/D5EE00640F.

[174] F. Yang , P. Tockhorn , A. Musiienko , et al., “Minimizing Interfacial Recombination in 1.8 eV Triple‐Halide Perovskites for 27.5% Efficient All‐Perovskite Tandems,” Advanced Materials 36 (2024): 2307743, 10.1002/adma.v36.6. 37988595

[175] Y.‐F. Wang , Y.‐F. Liu , H.‐W. Zhang , C. Liu , C.‐M. Jin , and J. Feng , “Enhanced Efficiency and Stability of Perovskite Solar Cells through Nanoimprinting Dual‐Functional Nanostructured PEAI/2D/3D Perovskite Interface,” Chemical Engineering Journal 497 (2024): 155686, 10.1016/j.cej.2024.155686.

[176] J. Wang , L. Bi , X. Huang , et al., “Bilayer Interface Engineering through 2D/3D Perovskite and Surface Dipole for Inverted Perovskite Solar Modules,” eScience 4 (2024): 100308, 10.1016/j.esci.2024.100308.

[177] X. Jiang , J. Zhang , S. Ahmad , et al., “Dion‐Jacobson 2D‐3D Perovskite Solar Cells with Improved Efficiency and Stability,” Nano Energy 75 (2020): 104892, 10.1016/j.nanoen.2020.104892.

[178] J.‐W. Lee , Y.‐T. Hsieh , N. De Marco , S.‐H. Bae , Q. Han , and Y. Yang , “Halide Perovskites for Tandem Solar Cells,” Journal of Physical Chemistry Letters 8 (2017): 1999, 10.1021/acs.jpclett.7b00374. 28422510

[179] C. Chen , J. Liang , J. Zhang , et al., “Interfacial Engineering of a Thiophene‐Based 2D/3D Perovskite Heterojunction for Efficient and Stable Inverted Wide‐Bandgap Perovskite Solar Cells,” Nano Energy 90 (2021): 106608, 10.1016/j.nanoen.2021.106608.

[180] Y. Xu , K. Chen , S. Gu , et al., “Constructing a p‐π Conjugated, Phase‐Pure DJ‐Type 2D Perovskite Modification Layer to Advance the Operation Stability to 15,160 hours for Efficient Perovskite Solar Cells,” Nano Energy 135 (2025): 110635, 10.1016/j.nanoen.2024.110635.

[181] T. Leijtens , K. A. Bush , R. Prasanna , and M. D. McGehee , “Opportunities and Challenges for Tandem Solar Cells Using Metal Halide Perovskite Semiconductors,” Nature Energy 3 (2018): 828, 10.1038/s41560-018-0190-4.

[182] R. Wang , T. Huang , J. Xue , J. Tong , K. Zhu , and Y. Yang , “Prospects for Metal Halide Perovskite‐Based Tandem Solar Cells,” Nature Photonics 15 (2021): 411, 10.1038/s41566-021-00809-8.

[183] Q. Liu , J. Jiang , and X. C. Zeng , “Quasi‐2D Ruddlesden–Popper Perovskites with Tunable Wide Bandgaps and Phosphonium as Additives for Interface Defect Passivation in Tandem Solar Cell Design,” Journal of Materials Chemistry A 13 (2025): 22604, 10.1039/D5TA02175H.

[184] J. Zhou , T. Wen , J. Sun , et al., “Phase‐Stable Wide‐Bandgap Perovskites with 2D/3D Structure for All‐Perovskite Tandem Solar Cells,” ACS Energy Letters 9 (2024): 1984, 10.1021/acsenergylett.4c00285.

[185] Z. Irshad , W. Lee , M. Adnan , Y. Choi , T. Park , and J. Lim , “Elucidating Charge Carrier Dynamics in Perovskite‐Based Tandem Solar Cells,” Small Methods 8 (2024): 2300238, 10.1002/smtd.v8.2. 37322273

[186] J. Wen , Y. Zhao , P. Wu , et al., “Heterojunction Formed via 3D‐to‐2D Perovskite Conversion for Photostable Wide‐Bandgap Perovskite Solar Cells,” Nature Communications 14 (2023): 7118, 10.1038/s41467-023-43016-5. PMC10628126 37932289

[187] F. Pei , Q. Chen , and Y. Jiang , “Stability of Wide‐Bandgap Perovskites for Tandem Applications: A Review,” Energy Material Advances 6 (2025): 0172, 10.34133/energymatadv.0172.

[188] X. Zhu , R. Zhang , M. Li , et al., “PEDOT:PSS/CuCl Composite Hole Transporting Layer for Enhancing the Performance of 2D Ruddlesden–Popper Perovskite Solar Cells,” Journal of Physical Chemistry Letters 13 (2022): 6101, 10.1021/acs.jpclett.2c01399. 35759218

[189] Q. Xu , K. Meng , Z. Liu , et al., “Synergistic Improvements in Efficiency and Stability of 2D Perovskite Solar Cells with Metal Ion Doping,” Advanced Materials Interfaces 6 (2019): 1901259, 10.1002/admi.v6.23.

[190] Q. Cheng , B. Wang , G. Huang , et al., “Impact of Strain Relaxation on 2D Ruddlesden–Popper Perovskite Solar Cells,” Angewandte Chemie International Edition 61 (2022): e202208264, 10.1002/anie.v61.36. 35789174

[191] C. Wang , J. Gu , J. Li , et al., “Two‐Dimensional (*n* = 1) Ferroelectric Film Solar Cells,” National Science Review 10 (2023): nwad061, 10.1093/nsr/nwad061. 37600562 PMC10434298

[192] R. Cao , K. Sun , C. Liu , et al., “Structurally Flexible 2D Spacer for Suppressing the Electron–Phonon Coupling Induced Non‐Radiative Decay in Perovskite Solar Cells,” Nano‐Micro Letters 16 (2024): 178, 10.1007/s40820-024-01401-9. 38656466 PMC11043286

[193] Q. Wang , X. Jiang , C. Peng , et al., “Regulating the Lattice Strain in Perovskite Films to Obtain Efficient and Stable Perovskite Solar Cells,” Chemical Engineering Journal 481 (2024): 148464, 10.1016/j.cej.2023.148464.

[194] D. Li , Z. Xing , X. Meng , X. Hu , T. Hu , and Y. Chen , “Selection of Functional Spacer Cations for Efficient 2D/3D Perovskite Solar Cells,” CCS Chemistry 5 (2023): 781, 10.31635/ccschem.023.202202409.

[195] G. Pica , L. Pancini , C. E. Petoukhoff , et al., “Photo‐Ferroelectric Perovskite Interfaces for Boosting VOC in Efficient Perovskite Solar Cells,” Nature Communications 15 (2024): 8753, 10.1038/s41467-024-53121-8. PMC11464595 39384782

[196] C. Xu , P. Hang , C. Kan , et al., “Molecular Ferroelectric Self‐Assembled Interlayer for Efficient Perovskite Solar Cells,” Nature Communications 16 (2025): 835, 10.1038/s41467-025-56182-5. PMC11743605 39828761

[197] J. Su , H. Cai , X. Ye , et al., “Efficient Perovskite Solar Cells Prepared by Hot Air Blowing to Ultrasonic Spraying in Ambient Air,” ACS Applied Materials & Interfaces 11 (2019): 10689, 10.1021/acsami.9b01843. 30799609

[198] K. Meng , B. Chen , M. Xiao , et al., “Humidity‐Insensitive, Large‐Area‐Applicable, Hot‐Air‐Assisted Ambient Fabrication of 2D Perovskite Solar Cells,” Advanced Materials 35 (2023): 2209712, 10.1002/adma.v35.11. 36579894

[199] Z. Bi , X. Rodríguez‐Martínez , C. Aranda , et al., “Defect Tolerant Perovskite Solar Cells from Blade Coated Non‐Toxic Solvents,” Journal of Materials Chemistry A 6 (2018): 19085, 10.1039/C8TA06771F.

[200] G. Wu , N. Ahmad , and Y. Zhang , “High‐Efficiency of 15.47% for Two‐Dimensional Perovskite Solar Cells Processed by Blade Coating with Non‐Thermal Assistance,” Journal of Materials Chemistry C 9 (2021): 9851, 10.1039/D1TC01926K.

[201] A. S. Subbiah , L. V. Torres Merino , A. R. Pininti , et al., “Enhancing the Performance of Blade‐Coated Perovskite/Silicon Tandems via Molecular Doping and Interfacial Energy Alignment,” ACS Energy Letters 9 (2024): 727, 10.1021/acsenergylett.4c00070.

[202] X. Zhang , P. Baral , N. Chakraborty , et al., “Blade Coating Inverted Perovskite Solar Cells with Vacuum‐Assisted Nucleation Based on Bottom Quasi‐2D Passivation,” Solar RRL 7 (2023): 2200900, 10.1002/solr.v7.5.

[203] Y. Wang , C. Duan , P. Lv , et al., “Printing Strategies for Scaling‐up Perovskite Solar Cells,” National Science Review 8 (2021): nwab075, 10.1093/nsr/nwab075. 34691715 PMC8363337

[204] B. Kang and F. Yan , “Emerging Strategies for the Large‐Scale Fabrication of Perovskite Solar Modules: from Design to Process,” Energy & Environmental Science 18 (2025): 3917, 10.1039/D4EE05613B.

[205] H. Eggers , F. Schackmar , T. Abzieher , et al., “Inkjet‐Printed Micrometer‐Thick Perovskite Solar Cells with Large Columnar Grains,” Advanced Energy Materials 10 (2020): 1903184, 10.1002/aenm.v10.6.

[206] Z. Yang , W. Zhang , S. Wu , et al., “Slot‐Die Coating Large‐Area Formamidinium‐Cesium Perovskite Film for Efficient and Stable Parallel Solar Module,” Science Advances 7 (2021): eabg3749, 10.1126/sciadv.abg3749. 33931458 PMC8087413

[207] O. J. Romero , L. E. Scriven , and M. D. S. Carvalho , “Effect of Curvature of Coating Die Edges on the Pinning of Contact Line,” AIChE Journal 52 (2006): 447, 10.1002/aic.v52:2.

[208] C. Teixeira , R. Fuentes‐Pineda , L. Andrade , A. Mendes , and D. Forgács , “Fabrication of Low‐Cost and Flexible Perovskite Solar Cells by Slot‐Die Coating for Indoor Applications,” Materials Advances 4 (2023): 3863, 10.1039/D3MA00285C.

[209] J. E. Bishop , J. A. Smith , and D. G. Lidzey , “Development of Spray‐Coated Perovskite Solar Cells,” ACS Applied Materials & Interfaces 12 (2020): 48237, 10.1021/acsami.0c14540. 32960040 PMC7596755

[210] R. Sahani , N. Kumar , C. A. R. Perini , et al., “2D/3D Heterostructure Halide Perovskite Thin Films through an Innovative Spray‐Deposition of Bulky Organic Cation‐Containing Ammonium Salts,” Advanced Materials Interfaces 12 (2025): 2400823, 10.1002/admi.v12.9.

[211] T. Okoroafor , A. Maalouf , S. Oez , V. Babu , B. Wilk , and S. Resalati , “Life Cycle Assessment of Inkjet Printed Perovskite Solar Cells,” Journal of Cleaner Production 373 (2022): 133665, 10.1016/j.jclepro.2022.133665.

[212] D. Lu , M. Jamshidi , J. M. Gardner , and L. Belova , “Scalable Fabrication of Perovskite Solar Cells with Inkjet‐Printed Perovskite Absorbers Processed under Ambient Conditions,” ACS Applied Materials & Interfaces 17 (2025): 28055, 10.1021/acsami.4c20567. 40299421 PMC12086759

[213] N. K. Pendyala , S. Magdassi , and L. Etgar , “Fabrication of Perovskite Solar Cells with Digital Control of Transparency by Inkjet Printing,” ACS Applied Materials & Interfaces 13 (2021): 30524, 10.1021/acsami.1c04407. 34160194

[214] V. S. Chirvony , G. Muñoz‐Matutano , I. Suárez , et al., “Achieving Inkjet‐Printed 2D Tin Iodide Perovskites: Excitonic and Electro‐Optical Properties,” Advanced Functional Materials 34 (2024): 2405154, 10.1002/adfm.v34.39.

[215] R. Pesch , A. Diercks , J. Petry , et al., “Hybrid Two‐Step Inkjet‐Printed Perovskite Solar Cells,” Solar RRL 8 (2024): 2400165, 10.1002/solr.v8.13.

[216] I. A. Howard , T. Abzieher , I. M. Hossain , et al., “Coated and Printed Perovskites for Photovoltaic Applications,” Advanced Materials 31 (2019): 1806702, 10.1002/adma.v31.26. 30932255

[217] C. Chen , C. Ran , Q. Yao , et al., “Screen‐Printing Technology for Scale Manufacturing of Perovskite Solar Cells,” Advanced Science 10 (2023): 2303992, 10.1002/advs.v10.28. 37541313 PMC10558701

[218] J. Song , G. Zhou , W. Chen , et al., “Unraveling the Crystallization Kinetics of 2D Perovskites with Sandwich‐Type Structure for High‐Performance Photovoltaics,” Advanced Materials 32 (2020): 2002784, 10.1002/adma.v32.36. 32697407

[219] A. Kulkarni , R. Sarkar , S. Akel , et al., “A Universal Strategy of Perovskite Ink ‐ Substrate Interaction to Overcome the Poor Wettability of a Self‐Assembled Monolayer for Reproducible Perovskite Solar Cells,” Advanced Functional Materials 33 (2023): 2305812, 10.1002/adfm.v33.47.

[220] M. Duan , J. Yang , T. Li , et al., “Mechanically Stable Screen‐Printed Flexible Perovskite Solar Cells via Selective Self‐Assembled Siloxane Coupling Agents,” Npj Flexible Electronics 9 (2025): 30, 10.1038/s41528-025-00407-6.

[221] J. Zhang , B. Liu , Z. Liu , et al., “Optimizing Perovskite Thin‐Film Parameter Spaces with Machine Learning‐Guided Robotic Platform for High‐Performance Perovskite Solar Cells,” Advanced Energy Materials 13 (2023): 2302594, 10.1002/aenm.v13.48.

[222] M. Um , S. L. Sanchez , H. Song , et al., “Tailoring Molecular Space to Navigate Phase Complexity in Cs‐Based Quasi‐2D Perovskites via Gated‐Gaussian‐Driven High‐Throughput Discovery,” Advanced Energy Materials 15 (2025): 2404655, 10.1002/aenm.v15.16.

[223] N. Meftahi , M. A. Surmiak , S. O. Fürer , et al., “Machine Learning Enhanced High‐Throughput Fabrication and Optimization of Quasi‐2D Ruddlesden–Popper Perovskite Solar Cells,” Advanced Energy Materials 13 (2023): 2203859, 10.1002/aenm.v13.38.

[224] W. Hussain , S. Sawar , and M. Sultan , “Leveraging Machine Learning to Consolidate the Diversity in Experimental Results of Perovskite Solar Cells,” RSC Advances 13 (2023): 22529, 10.1039/D3RA02305B. 37497089 PMC10367956

[225] Q. Tao , P. Xu , M. Li , and W. Lu , “Machine Learning for Perovskite Materials Design and Discovery,” NPJ Computational Materials 7 (2021): 23, 10.1038/s41524-021-00495-8.

[226] L. Klein , S. Ziegler , F. Laufer , et al., “Discovering Process Dynamics for Scalable Perovskite Solar Cell Manufacturing with Explainable AI,” Advanced Materials 36 (2024): 2307160, 10.1002/adma.v36.7. 37904613

[227] Y. Zhao , J. Zhang , Z. Xu , et al., “Discovery of Temperature‐Induced Stability Reversal in Perovskites Using High‐Throughput Robotic Learning,” Nature Communications 12 (2021): 2191, 10.1038/s41467-021-22472-x. PMC8044090 33850155

[228] M. M. Tavakoli , L. Gu , Y. Gao , et al., “Fabrication of Efficient Planar Perovskite Solar Cells Using a One‐Step Chemical Vapor Deposition Method,” Scientific Reports 5 (2015): 14083, 10.1038/srep14083. 26392200 PMC4585726

[229] S. S. Magubane , R. Burns , S. Ngqoloda , C. J. Oliphant , P. F. Miceli , and C. J. Arendse , “Sequential Chemical Vapor Deposition of Two‐Dimensional Sn–Pb Compound Perovskite Thin Films and Its Exciton Transport,” ACS Applied Electronic Materials 5 (2023): 5352, 10.1021/acsaelm.3c00266.

[230] M. T. Hoerantner , E. L. Wassweiler , H. Zhang , et al., “High‐Speed Vapor Transport Deposition of Perovskite Thin Films,” ACS Applied Materials & Interfaces 11 (2019): 32928, 10.1021/acsami.9b07651. 31416312 PMC6748557

[231] F. Sahli , N. Miaz , N. Salsi , et al., “Vapor Transport Deposition of Methylammonium Iodide for Perovskite Solar Cells,” ACS Applied Energy Materials 4 (2021): 4333, 10.1021/acsaem.0c02999.

[232] J. A. Raiford , S. T. Oyakhire , and S. F. Bent , “Applications of Atomic Layer Deposition and Chemical Vapor Deposition for Perovskite Solar Cells,” Energy & Environmental Science 13 (2020): 1997, 10.1039/D0EE00385A.

[233] L. Qiu , S. He , Y. Jiang , and Y. Qi , “Metal Halide Perovskite Solar Cells by Modified Chemical Vapor Deposition,” Journal of Materials Chemistry A 9 (2021): 22759, 10.1039/D1TA06459B.

[234] G. Tong , J. Zhang , T. Bu , et al., “Holistic Strategies Lead to Enhanced Efficiency and Stability of Hybrid Chemical Vapor Deposition Based Perovskite Solar Cells and Modules,” Advanced Energy Materials 13 (2023): 2300153, 10.1002/aenm.v13.21.

[235] Y. Wang , P. Lv , J. Pan , et al., “Grain Boundary Elimination via Recrystallization‐Assisted Vapor Deposition for Efficient and Stable Perovskite Solar Cells and Modules,” Advanced Materials 35 (2023): 2304625, 10.1002/adma.v35.44. 37466632

[236] A. Khorasani , F. Mohamadkhani , M. Marandi , H. Luo , and M. Abdi‐Jalebi , “Opportunities, Challenges, and Strategies for Scalable Deposition of Metal Halide Perovskite Solar Cells and Modules,” Advanced Energy and Sustainability Research 5 (2024): 2300275, 10.1002/aesr.v5.7.

[237] J. Borchert , I. Levchuk , L. C. Snoek , et al., “Impurity Tracking Enables Enhanced Control and Reproducibility of Hybrid Perovskite Vapor Deposition,” ACS Applied Materials & Interfaces 11 (2019): 28851, 10.1021/acsami.9b07619. 31314481 PMC7007011

[238] Q. Guesnay , F. Sahli , C. Ballif , and Q. Jeangros , “Vapor Deposition of Metal Halide Perovskite Thin Films: Process Control Strategies to Shape Layer Properties,” APL Materials 9 (2021): 100703, 10.1063/5.0060642.

[239] D. Liu , “Vapor Transport Deposition Technology for Perovskite Films,” Advanced Materials Interfaces 12 (2025): 2500064, 10.1002/admi.v12.12.

